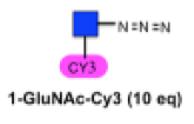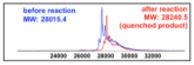# Site-Specific Glycoconjugation of Protein via Bioorthogonal Tetrazine Cycloaddition with a Genetically Encoded *trans*-Cyclooctene or Bicyclononyne

**DOI:** 10.1021/acs.bioconjchem.5b00101

**Published:** 2015-04-24

**Authors:** Takuya Machida, Kathrin Lang, Lin Xue, Jason W. Chin, Nicolas Winssinger

**Affiliations:** † Department of Organic Chemistry, NCCR Chemical Biology, University of Geneva, 30 quai Ernest Ansermet, 1211 Geneva, Switzerland; ‡ Technical University Munich, Institute for Advanced Study, Department of Chemistry, 4 Lichtenbergstraße, 85748 Garching, Germany; § Ecole Polytechnique Fédérale de Lausanne (EPFL), Institut des sciences et ingénierie chimiques, NCCR Chemical Biology, 1015 Lausanne, Switzerland; ⊥ Medical Research Council Laboratory of Molecular Biology, Francis Crick Avenue, Cambridge CB2 0GH, United Kingdom

## Abstract

Efficient access to proteins modified site-specifically with glycans is important in glycobiology and for therapeutic applications. Herein, we report a biocompatible protein glycoconjugation by inverse demand Diels–Alder reaction between tetrazine and *trans*-cyclooctene. Tetrazine functionalized glycans were obtained in one step by CuAAC (Cu-catalyzed alkyne azide cycloaddition) between glycosyl azide and an alkyne-tetrazine adduct. Site-specific glycoconjugation was performed chemoselectively on a target protein in which a *trans*-cyclooctene derivatized lysine was genetically encoded. Glycoconjugation proceeded to completion on purified protein and was shown to be selective for the target protein in *E. coli*.

## Graphical Abstract

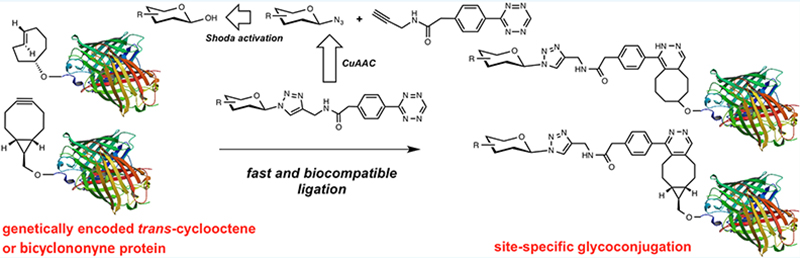

Protein glycosylation constitutes an important post-translational modification and the glycans of glycoproteins have been associated with numerous biological processes.^1^ For instance, cell surface glycoproteins are important in cellular recognition and have been implicated in embryonic development, lymphocyte trafficking, and cancer metastasis,^2–4^ and there is resurging interest in glycoconjugates as therapeutics.^5^ Accordingly, there is a strong interest in technologies to access homogeneous glycoconjugates and several methods have been reported to derivatize protein with functionalized glycan (Figure 1).^6–8^ Davis and co-workers pioneered the use of glycosylselenylsufide^9^ and glycosyl thiols^10^ for glycoconjugation to cysteine residues on a target protein. A free radical hydrothiolation using glycosyl thiol and a target protein containing an alkene (such as homoallyl glycine) was also reported.^11^ More recently, the CuAAC was harnessed to conjugate glycodendrons to a capsid protein genetically engineered to contain a homopropargyl glycine.^12^ Alternatively, a tyrosine-selective conjugation was used to introduce an alkyne into a target protein that was subsequently coupled to an azide-derivatized glycan via CuAAC.^13^ In parallel, a chemoenzymatic method has also been employed to convert a cysteine residue within a defined protein sequence to obtain an aldehyde that was subsequently conjugated to an aminooxy glycan.^14^ However, current technologies have been restricted to purified proteins. Performing a selective glycoconjugation in more complex systems (*lysates* or *in cellulo*) would require a bioorthogonal conjugation without toxic reagent that proceeds at a rate compatible with low or sub *μ*M concentration of target protein. The recent development of the inverse demand cycloaddition of a tetrazine with strained alkenes and alkynes fits these requirements.^15–19^ Furthermore, progress in genetic encoding of unnatural amino acid through genetic code expansion^20–22^ has now yielded an aminoacyl-tRNA synthetase/tRNA pair to introduce strained alkenes and alkynes.^23–25^ The fast kinetics and bioorthogonality of the tetrazine-strained alkene reaction make it ideal for live cell experiments. Herein we report a straightforward method to access tetrazine glycans and demonstrate their conjugation proteins containing genetically encoded unnatural amino acids.

To be broadly applicable, the synthesis of tetrazine glycan conjugates would ideally be achieved in a few steps directly from native (oligo)sacharides. Recently, Shoda reported a remarkable reaction to selectively activate the anomeric position of unprotected oligosaccharide providing simple and practical access to glycans with an azide at the anomeric position.^26^ Leveraging on this powerful reaction, we sought to conjugate a tetrazine via a CuAAC reaction to the glycans of interest. Surprisingly, there are no precedents for the combined use of these two powerful conjugation technologies (CuAAC/tetrazine cycloaddition).^27^ To evaluate the potential of the reaction we used alkyne **2**, obtained by coupling of propargyl amine with commercially available tetrazinylphenylacetic acid,^28^ and glucosyl azide **1** (Figure 2). Based on the potential reduction of tetrazine under the action of sodium ascorbate and copper, we started our investigation using a source of Cu(I), performing the reaction under oxygen-free conditions. As shown in Figure 2, the reaction proceeded smoothly with glucosyl azide affording the desired product in 81% isolated yield (Entry 1). However, applying the same conditions to oligosaccharides proved more problematic with significant formation of side products arising from oxidative degradation (entry 2, iododerivative **3b** and homodimer **3c**). We thus turned to the reductive conditions using CuSO_4_/sodium ascorbate. Controlling the amounts of copper and sodium ascorbate was critical to suppress tetrazine reduction (entry 3 vs 4). Conditions C (Figure 2) were found to yield the desired cycloaddition without detectable tetrazine degradation, affording the disaccharide–tetrazine adduct in 76% isolated yield.

The practicality of these conditions coupled to the expediency led us to explore these conditions with a broader set of substrates (Table 1). Glycosyl azides **1** were prepared from native carbohydrates using 2-chloro-1,3-dimethylimidazolium chloride (DMC) and sodium azide as previously reported.^26^ Next, the CuAAC reaction according to the optimized procedure afforded the cycloaddition adduct in 64–80% isolated yield. Gratifyingly, performing the reaction at lower glycan concentration (10–30 mM, entries 10–12 vs 200 mM, entries 1–9) still afforded useful yield of the desired product after HPLC purification (required based on the polarity of the product formed).

We next assessed the reactivity of the glycan–tetrazine conjugate in reaction with *trans*-cyclooctene (TCO). It has been shown that subtle change in the steric and electronic nature of the tetrazine can have notable impact on the reaction rate.^18^ Using Cy-3 labeled glucosamine-tetrazine conjugate (**3-GluNAc-Cy3**, see SI for full experimental details), we calculated the kinetics of cycloaddition using second-order and pseudo-first-order conditions, measuring the change in fluorescence over time (1 *μ*M of the glycan and 1 or 10 equiv of TCO, respectively). A second-order rate constant of 8649 M^−1^ s^−1^ was calculated which is consistent with previous analysis of related structures.^18^

## Site-Specific Protein Glycoconjugation

**Figure 3:**
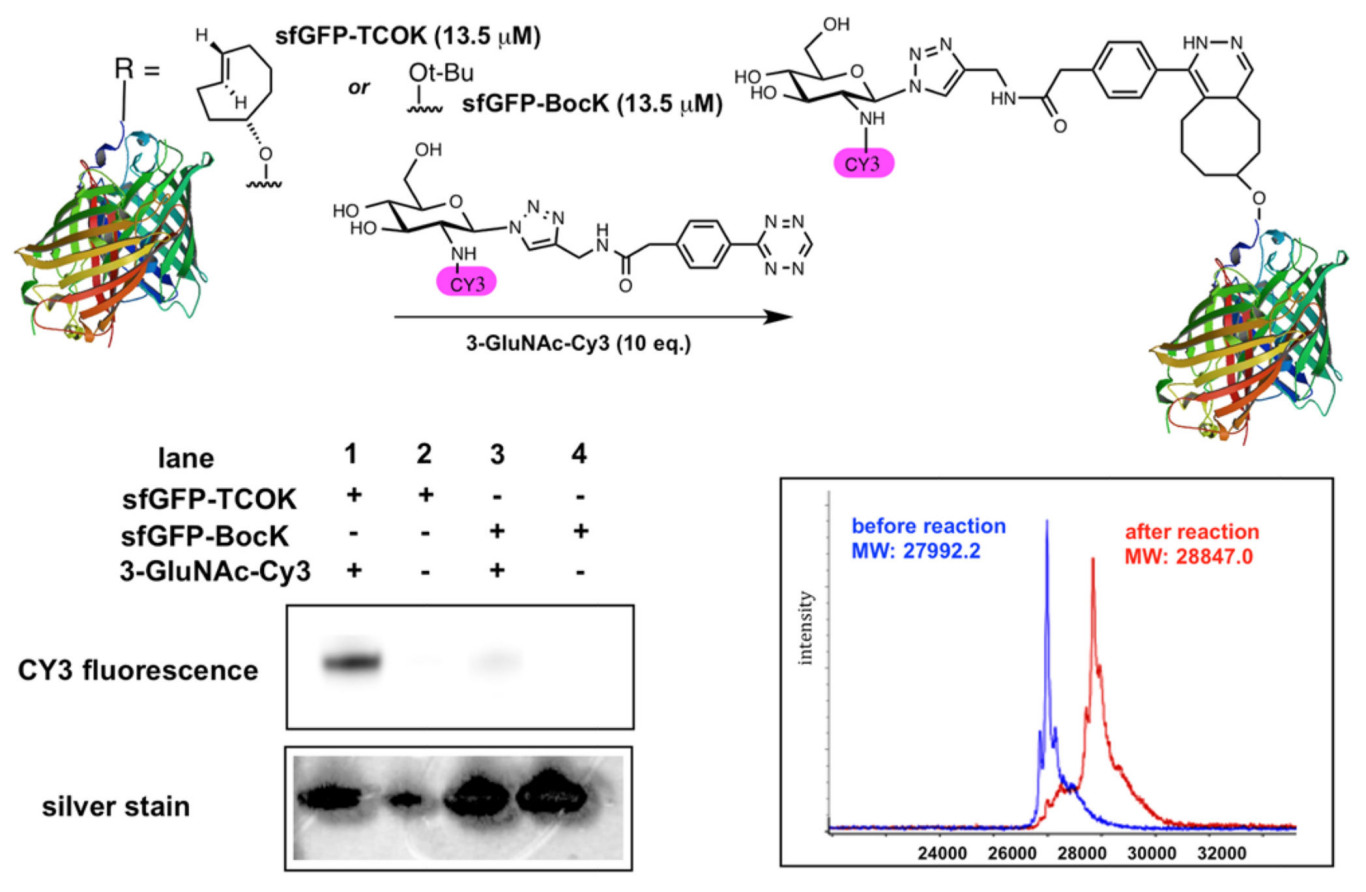
Glycoconjugation of **3-GluNAc-Cy3** to purified sfGFPTCOK or purified sfGFP-BocK. Bottom left panel: SDS-PAGE analysis of reactions; lower right panel: MALDI analysis of the reaction from lane 1.

We then evaluated the suitability of this chemistry to achieve site-selective glycan conjugation. First, purified sfGFP-TCOK and sfGFP-BocK (both proteins were prepared by incorporation of unnatural amino acid bearing TCO and Boc by *Mb*PylRS/tRNA_CUA_ pair into sfGFP overexpressed in *E. coli*)^24^ at 13.5 *μ*M were incubated with **3-GluNAc-Cy3** (10 equiv) for 12 h in Tris buffer at 37 °C. As controls, the same reaction was performed with sfGFP-BocK and in the absence of tetrazine **3-GluNAc-Cy3**. As shown in Figure 3, SDS-PAGE analysis of the conjugation reaction showed a strong fluorescent band corresponding to the conjugation of sfGFP-TCOK with tetrazine–glycan adduct (lane 1) but not in the controls (lanes 2–4). Analysis of the crude reaction mixture by MALDI-TOF showed a complete conversion with a mass gain corresponding the cycloaddition product and N_2_ extrusion. Considering the rate constant of the TCO–tetrazine conjugation, these conditions are very forceful; however, they illustrate the high chemoselectivity of the reaction and stability of the product (no degradation of the product is observed after 12 h).

We next investigated the kinetics of the reaction with genetically encoded bicyclononyne (sfGFP-BCNK, prepared according to the same procedure as sfGFP-TCOK).^24^ Tetrazine conjugation with bicyclononyne^29^ was reported to be 10–15 times slower than with TCO^24^ and should provide a more stringent test for the reactivity of tetrazine conjugates **3**. As shown in Table 2, conjugation of **3-GluNAc-Cy3** with sfGFP-BCNK at the same concentrations as used in sfGFP-TCOK afforded the desired glycoconjugation after 10 min (entry 1, reactions were quenched with 100 equiv of TCO). Reducing protein concentration to 1 *μ*M or 100 nM and glycan equivalence to 5 equiv still afforded the desired glycoconjugates (entry 2–4). At 100 nM concentration of sfGFP-BCNK (entry 4), traces of starting material are still present after 10 min indicating that these conditions approach the limit of reactivity. BCN is known to also undergo clycoaddition with azide-functionalized substrates, albeit with slower rates than with tetrazine. To compare the reactivity of the two conjugation methods, sfGFP-BCNK was reacted with glucosyl azide **1-GluNAc-Cy3** under the same forceful conditions as used in entry 1, namely, 13.5 *μ*M of protein with 10 equiv of glycan. After 10 min, the reaction was quenched with tetrazine **2** (Entry 5) yielding the quenched product without notable glycoconjugation. Extending the reaction to 3 h afforded ca. 30% of the conjugation product. Thus, while glycoconjugation with glycosyl azide **1** with genetically BCN proteins is possible over extended time, the data in Table 2 clearly demonstrates the superiority of glycosyl terazine **3** over glycosyl azide **1** in glycoconjugations.

**Figure 4:**
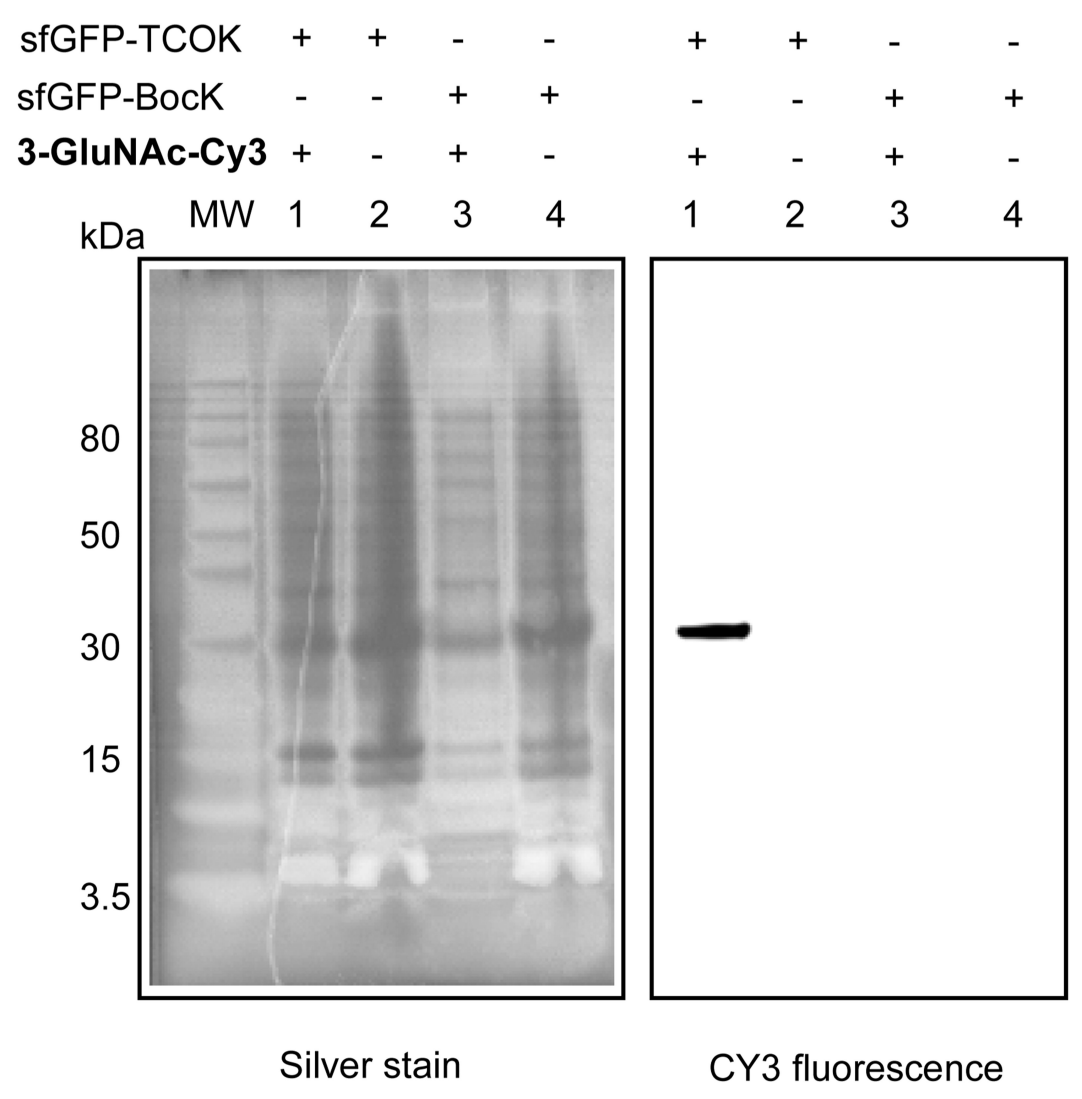
Glycoconjugation of **3-GluNAc-Cy3** in *E. coli*. SDS-PAGE analysis of the reaction with silver staining (left) and fluorescence scanning (Cy3, right).

We then investigated the specificity of the glycoconjugation in *E. coli* expressing sfGFP-TCOK. Cells were pelleted, washed with PBS to remove excess TCOK present in the medium, and incubated with **3-GluNAc-Cy3** (25 *μ*M; the Cy-3 is not sulfated and cell permeable^30^) for 8 h at 37 °C. As controls, the same reaction was performed on *E. coli* expressing sfGFP-BocK and in the absence of tetrazine **3-GluNAc-Cy3**. After the reaction, lysis buffer (LDS) was added, and the crude mixture was analyzed by SDS-PAGE (silver stain and fluorescence scan for Cy-3) showing that a single protein migrating at the molecular weight of GFP underwent conjugation (lane 1, Figure 4), whereas the control reaction showed no conjugation (lanes 2–4). Taken together, the data is consistent with a highly specific glycoconjugation of sfGFP-TCOK (lane5) within *E. coli*.

In summary, we have developed a simple method to rapidly access tetrazine functionalized glycans from native carbohydrates. The work reported establishes the compatibility of CuAAC with inverse electron demand Diels–Alder reaction. It is noteworthy that tetrazine adduct can be prepared with complex glycans (hexasaccharide) and the more challenging sialyl-type oligosaccharides. Rapid, site-specific glycoconjugation was achieved using genetically encoded *trans*-cyclooctene (TCO) and bicyclononyne (BCN) modified unnatural amino acids. The reaction was shown to be suitable for performing glycoconjugation in *E. coli*. We anticipate that the fast kinetics of the tetrazine cycloaddition coupled to the bioorthogonality of this reaction will facilitate the preparation of tailored glycoproteins.

## Supplementary Material

SI

## Figures and Tables

**Figure 1:**
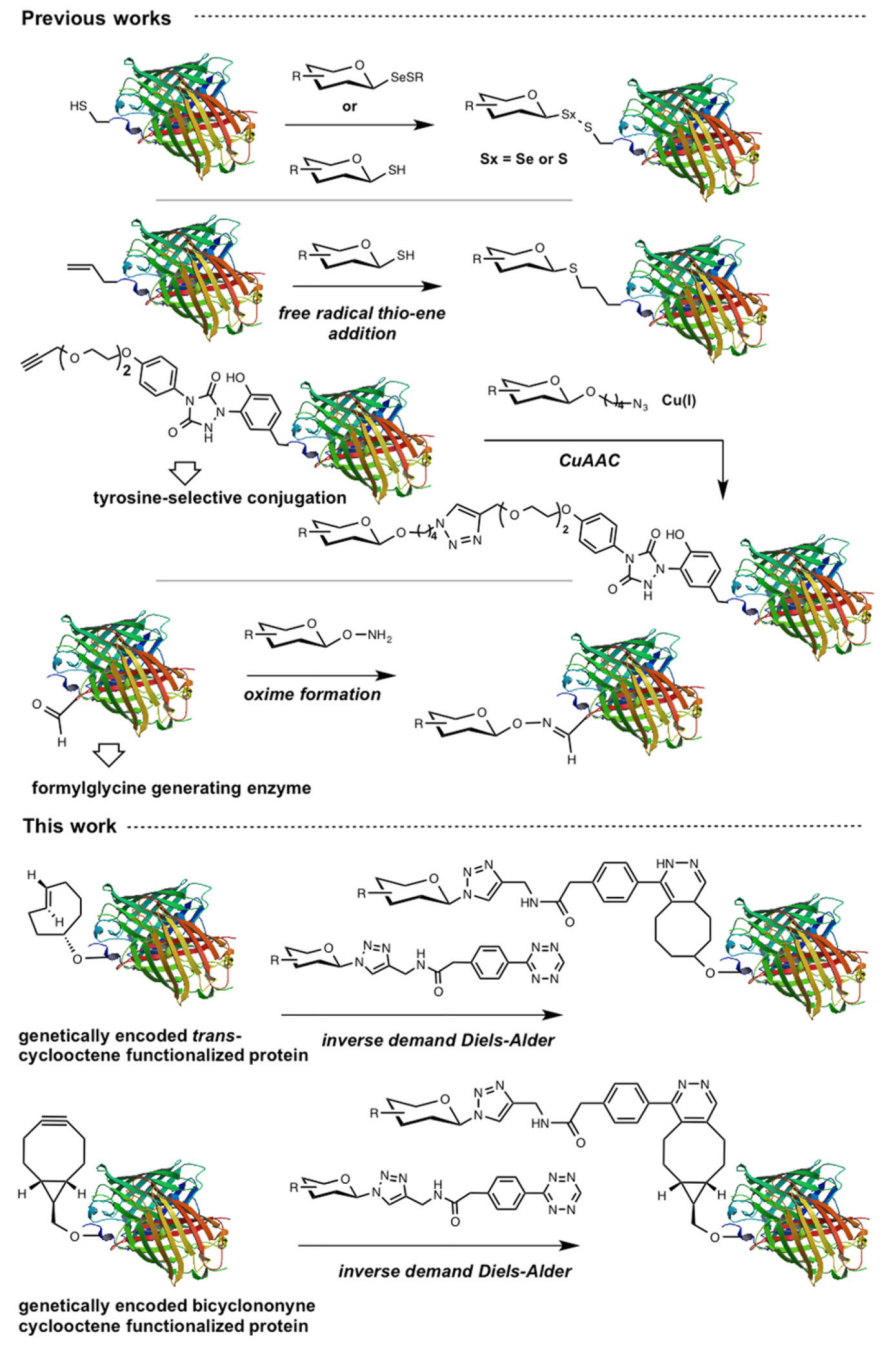
Schematic representation of glycoconjugation technologies.

**Figure 2:**
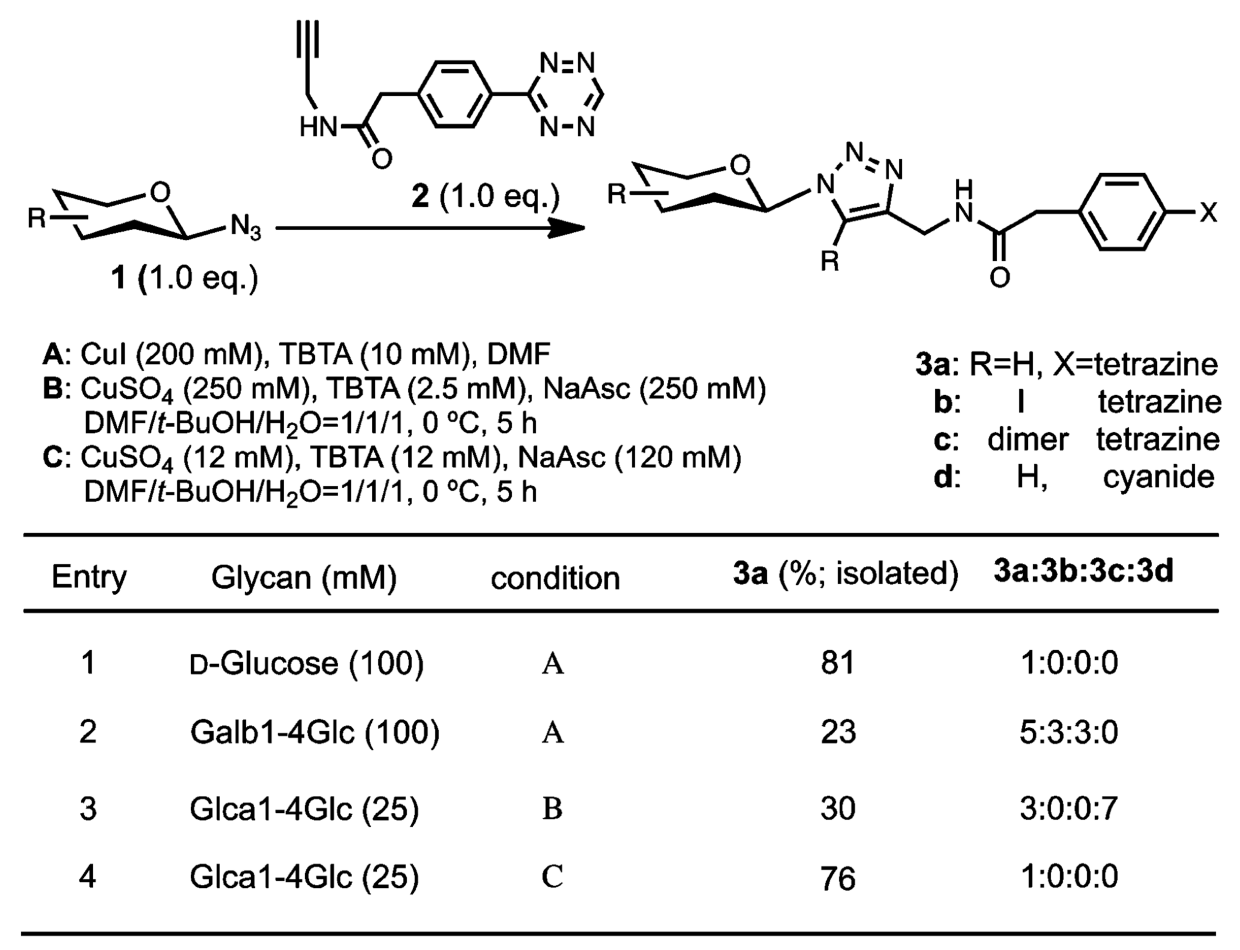
CuAAC conjugation of glycosyl azide (**1**) with alkynetetrazine (**2**).

**Table 1:** CuAAcC Conjugation of Alkyne-Tetrazine (2) with a Panel of Glycans 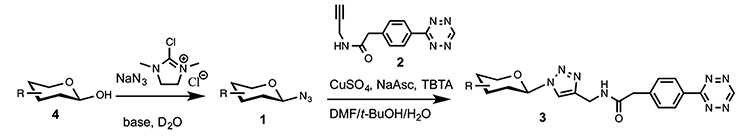

Entry	Glycan (mM)		base	**1** [%]	anomeric configuration	**3** [%]
1	**4a**: d-Glucose (200)	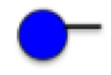	Et_3_N	98	β	80
2	**4b**: d-Mannose (200)	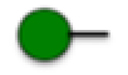	Et_3_N	85	α	76
3	**4c**: d-Galactose (200)	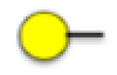	Et_3_N	53^a^	β	50
4	**4d**: l-Fucose (200)	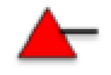	Et_3_N	80^a^	β	72
5	**4e**: N-Acetylglucosamine (200)	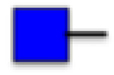	2,6-lutidine	97	β	70
6	**4f**: d-Glucuronic acid (200)	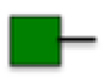	Et_3_N	50	β	57
7	**4g**: Glca1-4Glc (200)	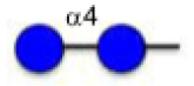	Et_3_N	74	β	76
8	**4h**: Glca1-4Glc (200)	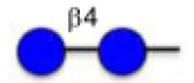	Et_3_N	82	β	72
9	**4i**: Galb1-4Glc (200)	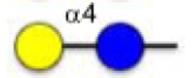	Et_3_N	99	β	64
10	**4j**: Fuca1-2Galb1-3[Fuca1-4] GlcNAcb1-3Galb1-4Glc (10)	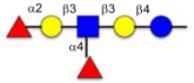	Et_3_N	78	β	20
11	**4k**: Neu5Aca2-3Galb1-4Glc (30)	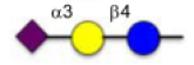	Et_3_N	80	β	21
12	**4l**: Neu5Aca2-6Galb1-4Glc (30)	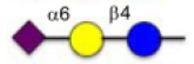	Et_3_N	91	β	17

**Table 2:** Glycoconjugation of sfGFP 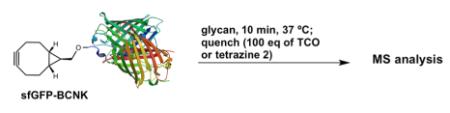

Entry	sfGFP(μM)	Glycan (equivalents)	MALDI
**1**	**13.5**	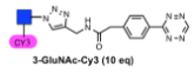	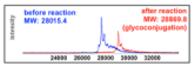
**2**	**1.0**	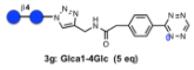	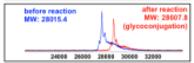
**3**	**1.0**	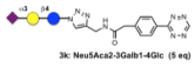	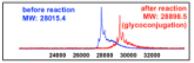
**4**	**1.0**	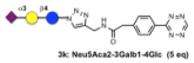	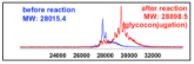
**5**	**13.5**