# Supplementary material for: Site-Specific Glycoconjugation of Protein via Bioorthogonal Tetrazine Cycloaddition with a Genetically Encoded trans-Cyclooctene or Bicyclononyne
Source: Bioconjug Chem. Author manuscript; Available in PMC 2015 Dec 9. (PMC4673905; doi:10.1021/acs.bioconjchem.5b00101)
Supplement: SI [file NIHMS66188-supplement-SI.pdf]

# Supporting Information

## Site-Specific Glycoconjugation of Protein via Bioorthogonal Tetrazine Cycloaddition with a Genetically Encoded *trans*-Cyclooctene or Bicyclononyne

Takuya Machida[†], Kathrin Lang[‡], Lin Xue,[∮] Jason W. Chin[⊥] and Nicolas Winssinger*[†]

[†]Department of Organic chemistry, NCCR Chemical Biology, University of Geneva, 30 quai Ernest Ansermet, 1211 Geneva, Switzerland
[‡]Technical University Munich, Institute for Advanced Study, Department of Chemistry, 4 Lichtenbergstraße, 85748 Garching, Germany
[∮]Ecole Polytechnique Fédérale de Lausanne (EPFL), Institut des sciences et ingénierie chimiques, NCCR Chemical Biology, 1015 Lausanne, Switzerland
[⊥]Medical Research Council Laboratory of Molecular Biology, Hills Road, Cambridge CB2 0GH, UK

### Table of Contents

1. General……………………………………………………………….……..2
2. Synthesis of alkyne-substituted tetrazine **2**……………….…………2
3. Preparation of azidoglycans **1** …………………………….…………3
4. Synthesis of glycan-tetrazine conjugates **3**…………………………10
5. Synthesis of 3-GluNAc-Cy3………………………………………………15
6. Synthesis of *trans*-cyclooctene derivative S7 for kinetic study …...17
7. Kinetic study of tetrazine-TCO cycloaddition………………………...19
8. Protein conjugation with purified *sf*GFP-TCOK……………….…….21
9. Protein conjugation with purified *sf*GFP-BCNK……………….…….21
10. Protein conjugation in *E. coli* expressing *sf*GFP-TCOK……..……21
11. MALDI analysis of glycoconjugation...………………………….…….22
12. Spectra…………………………………………………………….…….26
13. References……………………………………………………………….48

# 1. General

All reagents and solvents for the organic synthesis were purchased from commercial sources and were used without further purification. Anhydrous solvents were obtained by passing them through commercially available alumina column (Innovative Technology, Inc., ® VA). Acidic resin DOWEX® 50WX2 was generated by shaking the resin with 2M HCl and washing with deionized waster before use. Reverse phase purification was performed using Isolera Biotage with SNAP Cartridge KP-C18-HS of 60g or 12g. HPLC purification was performed with an Agilent 1260 infinity using ZORBAX 300SB-C18 column (9.4 x 250 mm). NMR spectra were measured using the following instruments Bruker 300 UltraShield (300 MHz), Bruker AVANCE 3 HD (400 MHz), Bruker 500 UltraShield (500 MHz) using deuterated solvents ($CDCl_3$, $CD_3OD$, or $D_2O$) referenced to undeuterated peak ($CDCl_3$ (δ=7.26) $CD_3OD$ (δ=3.31) $D_2O$ (δ=4.79)). LC-MS spectra were recorded using a DIONEX Ultimate 3000 UHPLC with a PINNACLE DB C18 column (1.9 μm, 50 x 2.1 mm) and with a Thermo LCQ Fleet Mass Spectrometer System operated in positive ion mode in electrospray ionization (ESI). MALDI-TOF Mass spectra were recorded using a Bruker Daltonics Autoflex spectrometer operated in positive ion mode. Fluorescence intensities were measured using a Molecular Devices Spectra Max M5. Electrophoresis was performed using Novex NuPAGE 4-12% Bis-Tris Gel. In gel fluorescence analysis were performed using a GE Healthcare Ettan DIGE Imager.

# 2. Synthesis of alkyne-substituted tetrazine 2

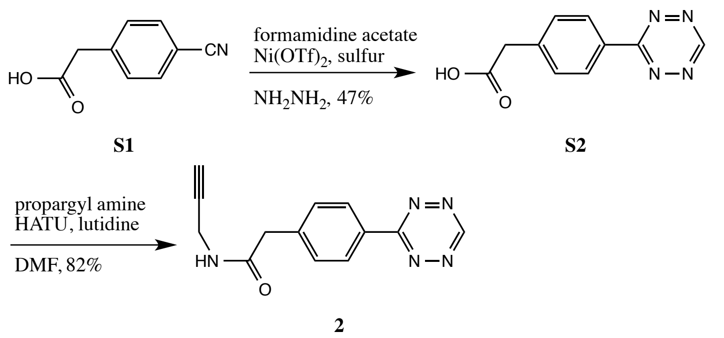


### 2-(4-(1,2,4,5-tetrazin-3-yl)phenyl)acetic acid (S2)

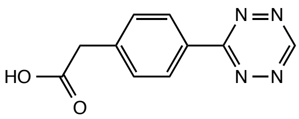


**S2**

Tetrazine phenyl acetic acid (**S2**, CAS: 1380500-92-4) is a commercial product. Conversely, it can be prepared according to the following procedure: To the mixture of 2-(4-cyanophenyl)acetic acid **S1** (687 mg, 4.27 mmol), nickel triflate (269 mg, 0.75 mmol), sulfur (136 mg, 4.27 mmol), formamidine acetate (4.44g, 42.7 mmol) was added anhydrous hydrazine (6.70 ml, 214 mmol, prepared by drying hydrazine monohydrate using flamed dried 3Å molecular sieves overnight) and stirred at 30°C for 24 h under nitrogen atmosphere. The reaction mixture was transfered to 500ml glass beaker and sodium nitrite solution (15.3 g, 222 mmol) in water (45 ml) was added to the reaction mixture followed by

1M HCl until gas evolution ceased and the pH reached 3. The mixture was extracted with $CH_2Cl_2$ and the organic phase was washed with brine, dried over sodium sulfate, filtrated and concentrated *in vacuo*. The residue was purified by silica gel column chromatography ($CH_2Cl_2$:MeOH=50:1) to obtain **S2** as a red solid (429 mg, 47%). NMR data for this compound matched literature report.[1]
$H^1$ NMR (300 MHz, $CDCl_3$): δ=10.22 (s, 1H), 8.62 (d, *J* = 8.4 Hz, 2H), 7.55 (d, *J* = 8.4 Hz, 2H), 3.80 (s, 2H).

### 2-(4-(1,2,4,5-tetrazin-3-yl)phenyl)-*N*-(prop-2-yn-1-yl)acetamide (2)

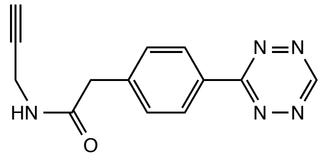


**2**

To a solution of **S2** (100 mg, 0.46 mmol), 2,6-lutidine (107 μl, 0.92 mmol) in dry DMF (3 ml) was added HATU (350 mg, 0.92 mmol) and the reaction mixture was stirred for 5 min at 0 °C under nitrogen atmosphere. Propargyl amine (68 μl, 1.06 mmol) was then added and the reaction was stirred for 2 h at 0 °C. Saturated sodium bicarbonate solution was added to the reaction mixture and the product was extracted with $CH_2Cl_2$. The organic phase was washed with brine, dried over sodium sulfate, filtrated and concentrated *in vacuo*. The residue was purified by silica gel column chromatography ($CH_2Cl_2$:MeOH=100:1) furnishing **2** as a red solid (95 mg, 82%).
$H^1$ NMR (400 MHz, $D_2O$): δ=10.23 (s, 1H), 8.63 (d, *J* = 8.4 Hz, 2H), 7.54 (d, *J* = 8.4 Hz, 2H), 4.07 (dd, *J* = 2.4 Hz, 5.2 Hz, 2H), 3.71 (s, 2H), 2.22 (t, *J* = 2.4 Hz, 1H); $C^{13}$ NMR (100 MHz, $D_2O$): δ=169.4, 166.2, 157.9, 139.8, 130.8, 130.4, 128.9, 79.1, 71.9, 43.4, 29.5 ; LRMS (ESI): calcd. for $C_{13}H_{12}N_5O$ $[M+H]^+$, 254.11. found 254.13.

## 3. Preparation of azide-glycan 1

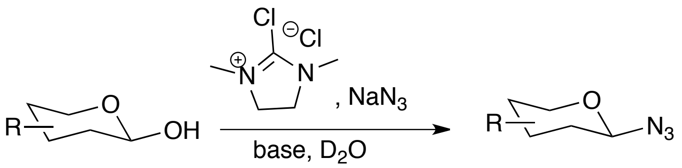


4 1

### General procedures

Azido glycan **1** were prepared according to the procedure reported by *Shoda et al.*[2] Briefly, a mixture of reducing glycan **4**, $Et_3N$, 2-chloro-1.3-dimethylimidazolium chloride (DMC), $NaN_3$, in 1,4-dioxane/ $H_2O$=1/1 was stirred for 1 h at – 10 °C. The solvent was evaporated and the residue was purified directly by column chromatography. In some cases, it was found to be more practical to transiently acetylate the product for purification purposes. For convenience, the reactions were also performed in $D_2O$ which facilitates reaction monitoring by NMR. In a

2.0ml Eppendorf tube, glycan **4**, $NaN_3$, 2-chloro-1.3-dimethylimidazolium chloride (DMC), and $Et_3N$ or 2,6-lutidine were added to $D_2O$. The mixture was shaken vigorously until the mixture became homogeneous. The mixture was then transferred to an NMR tube and the progress was monitored by $H^1$-NMR with intermittent shaking. Upon completion, the reaction was transferred back to the Eppendorf and lyophilized.

To the residue was added $Ac_2O$ (300 μl) and pyridine (1 ml) and the reaction mixture was stirred overnight at room temperature. The reaction was concentrated *in vaccuo* and the product was isolated by reverse phase chromatography (12g of C18 column, 50 ml of $H_2O$, then 100 ml of acetonitrile). The acetonitrile fraction was concentrated *in vacuo* then lyophilized. To the residue was added sodium methoxide (6 mg, 0.1 mmol) and dry methanol. The reaction mixture was stirred overnight under nitrogen atmosphere. The reaction mixture was neutralized with acidic resin (DOWEX® 50WX2), filtered and lyophilized.

After filtration, the mixture was concentrated *in vacuo* and the residue was purified by reverse phase column (12g of C18 column, flowed 200 ml of $H_2O$ ). Lyophilizing solvent gave desired azide-suger.

The mixture was then tra 1 M NaOH (100 μl) and stirred 3 h.

## β-*D*-Glucopyranosyl azide (1a)

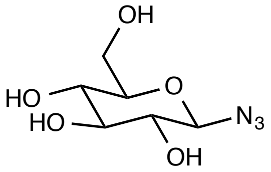


*D*-Glucose monohydrate (198 mg, 1.0 mmol), DMC (508 mg, 3.0 mmol), $NaN_3$ (584 mg, 9.0 mmol), triethylamine (1.25 ml, 9.0 mmol) in $H_2O$ (2 ml) and 1,4-dioxane (2 ml) were used. Silica gel column chromatography ($CH_2Cl_2$:MeOH=5:1) gave β-*D*-glucopyranosyl azide (200 mg, 98%) as colorless solid. Spectral characteristics of this compound matched the reported data.[2]

$H^1$ NMR (400 MHz, $D_2O$): δ=4.74 (d, *J* = 8.4 Hz, 1H), 3.92 (dd, *J* = 2.0 Hz, 12.4 Hz, 1H), 3.75 (dd, *J* = 5.6 Hz, 12.4 Hz, 1H), 3.49-3.56 (m, 2H), 3.42 (t, *J* = 10.0 Hz, 1H), 3.26 (t, *J* = 8.4 Hz, 1H)

## α-*D*-Mannopyranosyl azide (1b)

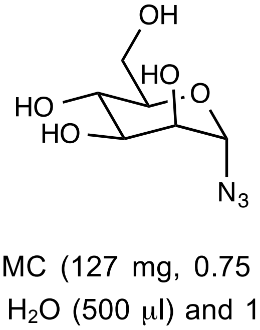


*D*-Mannose (45 mg, 0.25 mmol), DMC (127 mg, 0.75 mmol), $NaN_3$ (146 mg, 2.25 mmol), triethylamine (313 μl, 2.25 mmol) in $H_2O$ (500 μl) and 1,4-dioxane (500 μl) was used. Silica gel column chromatography ($CH_2Cl_2$:MeOH=5:1) afforded α-*D*-mannopyranosyl azide (41

mg, 80%) as colorless solid. Spectral characteristics of this compound matched the reported data.[3]
H[1] NMR (400 MHz, $D_2O$): δ=5.38 (d, *J* = 2.0 Hz, 1H), 3.79-3.85 (m, 2H), 3.64-3.73 (m, 3H), 3.57 (t, *J* = 9.6 Hz, 1H)

### *D*-Galactopyranosyl azide (1c)

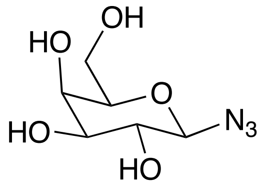


*D*-Galactose (54 mg, 0.3 mmol), DMC (153 mg, 0.9 mmol), $NaN_3$ (291 mg, 4.5 mmol), triethylamine (375 μl, 2.7 mmol) in deutrium oxide (1.2 ml) were shaken for 1 h at room temperature to afford a mixture of α and β anomers. The solvent was evaporated *in vacuo* and to the residues were treated with $Ac_2O$ (900 μl) and pyridine (3 ml). After stirring overnight at room temperature, the solvent was removed *in vacuo* and the residues were purified by silica gel column chromatography (Pentane:EtOAc=10:1) to separate α and β anomers. Each anomer was dissolved in dry methanol (1 mL) and treated with sodium methoxide (6 mg, 0.1 mmol). The reaction mixture was stirred overnight under nitrogen atmosphere. The mixture was neutralized with acidic resin (DOWEX® 50WX2), diluted with water (5 mL) and lyophilized to obtain α-*D*-Galactopyranosyl azide (9 mg, 15%) as colorless solid and β-*D*-Galactopyranosyl azide (33 mg, 53%) as colorless solid. Spectral characteristics of this compound matched the reported data.[3]
α anomer: H[1] NMR (400 MHz, $D_2O$): δ=5.56 (d, *J* = 4.4 Hz, 1H), 4.07 (m, 1H), 3.98 (d, *J* = 2.8 Hz, 1H), 3.93 (dd, *J* = 4.4 Hz, 10.4 Hz 1H), 3.73-3.80 (m, 3H); C[13] NMR (100 MHz, $D_2O$): δ=89.4, 73.0, 69.2, 69.0, 67.6, 61.2
β anomer: H[1] NMR (300 MHz, $D_2O$): δ=4.65 (d, *J* = 8.7 Hz, 1H), 3.94 (d, *J* = 3.3 Hz, 1H), 3.72-3.80 (m, 3H), 3.66 (dd, *J* = 3.3 Hz, 9.6 Hz 1H), 3.49 (dd, *J* = 8.7 Hz, 9.6 Hz 1H).

### *L*-Fucopyranosyl azide (1d)

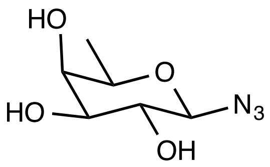


*L*-Fucose (48 mg, 0.3 mmol), DMC (153 mg, 0.9 mmol), $NaN_3$ (291 mg, 4.5 mmol), triethylamine (375 μl, 2.7 mmol) in deutrium oxide (1.2 ml) were shaken for 1 h at room temperature. The solvent was evaporated *in vacuo* and to the residues were treated with $Ac_2O$ (900 μl) and pyridine (3 ml). After stirring overnight at room temperature, the solvent was removed *in vacuo* and the residues were purified by silica gel column chromatography (Pentane:EtOAc=10:1) to separate α and β anomers. Each anomer was dissolved in dry

methanol (1 mL) and treated with sodium methoxide (6 mg, 0.1 mmol). The reaction mixture was stirred overnight under nitrogen atmosphere. The mixture was neutralized with acidic resin (DOWEX® 50WX2), diluted with water (5 mL) and lyophilized to obtain α-L-Fucopyranosyl azide (7 mg, 12%) as colorless solid and β-L-Fucopyranosyl azide (45 mg, 80%) as colorless solid.

α anomer: $H^1$ NMR (400 MHz, $D_2O$): δ=5.50 (d, *J* = 4.4 Hz, 1H), 4.19 (q, *J* = 6.4 Hz, 1H), 3.88 (dd, *J* = 4.4 Hz, 10.0 Hz, 1H), 3.81 (d, *J* = 3.2 Hz, 1H), 3.75 (dd, *J* = 3.2 Hz, 10.0 Hz , 1H), 1.24 (d, *J* = 6.4 Hz, 1H); $C^{13}$ NMR (100 MHz, $D_2O$): δ=89.6, 71.5, 69.3, 68.9, 67.3, 15.4.

β anomer: $H^1$ NMR (400 MHz, $D_2O$): δ=4.63 (d, *J* = 8.8 Hz, 1H), 3.88 (q, *J* = 6.8 Hz, 1H), 3.78 (d, *J* = 3.6 Hz, 1H), 3.67 (dd, *J* = 3.6 Hz, 10.0 Hz, 1H), 3.47 (dd, *J* = 8.8 Hz, 10.0 Hz , 1H), 1.27 (d, *J* = 6.8 Hz, 1H); $C^{13}$ NMR (100 MHz, $D_2O$): δ=90.5, 73.1, 72.8, 71.1, 70.0, 15.3

**2-Acetamido-2-deoxy-β-*D*-glucopyranosyl azide (1e)**

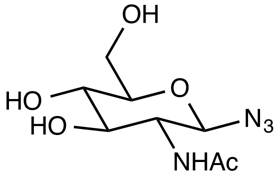


*N*-acetyl-*D*-glucosamine (22 mg, 0.1 mmol), DMC (51 mg, 0.3 mmol), $NaN_3$ (97 mg, 1.5 mmol), 2,6-lutidine (70 μl, 0.6 mmol) in deutrium oxide (500 μl) were shaken for 36 h. The reaction was lyophilized and purified by silica gel column chromatography ($CH_2Cl_2$:MeOH=5:1) to afford 2-acetamido-2-deoxy-β-*D*-glucopyranosyl azide **1e** (24 mg, 97%). as colorless solid. Spectral characteristics of this compound matched the reported data.[4]

$H^1$ NMR (400 MHz, $D_2O$): δ=4.76 (d, *J* = 9.2 Hz, 1H), 3.93 (d, *J* = 12.4 Hz, 1H), 3.69-3.79 (m, 2H), 3.48-3.60 (m, 3H), 2.06 (s, 3H).

**β-*D*-Glucopyranuronic azide (1f)**

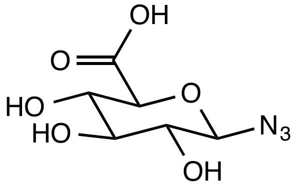


*D*-glucuronic acid (19 mg, 0.1 mmol), DMC (85 mg, 0.5 mmol), $NaN_3$ (58 mg, 0.9 mmol), triethylamine (125 μl, 0.9 mmol) in deutrium oxide (500 μl) were shaked for 1 h, lyophilized and purified by reversed phase chromatography to afford β-*D*-glucopyranuronic azide **1f** (11 mg, 50%) as colorless solid.

$H^1$ NMR (400 MHz, $D_2O$): δ=4.82 (d, *J* = 8.8 Hz, 1H), 3.99 (d, *J* = 10.0 Hz, 1H), 3.54-3.60 (m, 2H), 3.32 (dd, *J* = 8.8 Hz, 9.2 Hz, 1H); $C^{13}$ NMR (100 MHz, $D_2O$): δ=173.3, 90.0, 76.7, 75.4, 72.4, 71.1.

**β-*D*-Maltosyl azide (1g)**

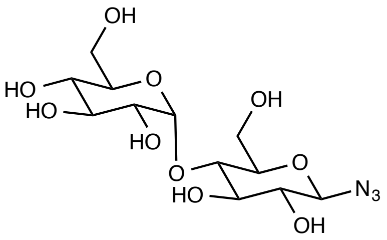

*D*-Maltose monohydrate (36 mg, 0.1 mmol), DMC (85 mg, 0.5 mmol), $NaN_3$ (97 mg, 1.5 mmol), triethylamine (208 μl, 1.5 mmol) in deutrium oxide (500 μl) were shaken for 1 h and lyophilized. To the residue was added $Ac_2O$ (300 μl) and pyridine (1 ml) and the reaction mixture was stirred overnight at room temperature. The reaction was concentrated *in vaccuo* and the product was isolated by reverse phase chromatography (12g of C18 column, 50 ml of $H_2O$, then 100 ml of acetonitrile). The acetonitrile fraction was concentrated *in vacuo* then lyophilized. To the residues was added sodium methoxide (6 mg, 0.1 mmol) and dry methanol. The reaction mixture was stirred overnight under nitrogen atmosphere. The reaction mixture was neutralized with acidic resin (DOWEX® 50WX2), filtered and lyophilized to afford β-*D*-maltosyl azide **1g** (27 mg, 74%) as colorless solid. Spectral characteristics of this compound matched the reported data.[2]

$H^1$ NMR (400 MHz, $D_2O$): δ=5.41 (d, *J* = 4.0 Hz, 1H), 4.75 (d, *J* = 8.8 Hz, 1H), 3.94 (d, *J* = 12.4 Hz, 1H), 3.66-3.87 (m, 8H), 3.57 (dd, *J* = 4.0 Hz, 10.0 Hz, 1H), 3.41 (t, *J* = 9.2 Hz, 1H), 3.30 (t, *J* = 9.2 Hz, 1H)

**β-*D*-Cellobiosyl azide (1h)**

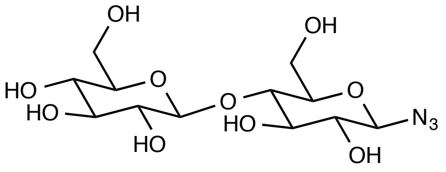

*D*-Cellobiose (34 mg, 0.1 mmol), DMC (85 mg, 0.5 mmol), $NaN_3$ (97 mg, 1.5 mmol), triethylamine (208 μl, 1.5 mmol) in deutrium oxide (500 μl) were shaken for 1 h and lyophilized. To the residue was added $Ac_2O$ (300 μl) and pyridine (1 ml) and the reaction mixture was stirred overnight at room temperature. The reaction was concentrated *in vaccuo* and the product was isolated by reverse phase chromatography (12g of C18 column, 50 ml of $H_2O$, then 100 ml of acetonitrile). The acetonitrile fraction was concentrated *in vacuo* then lyophilized. To the residues was added sodium methoxide (6 mg, 0.1 mmol) and dry methanol. The reaction mixture was stirred overnight under nitrogen atmosphere. The reaction mixture was neutralized with acidic resin (DOWEX® 50WX2), filtered and lyophilized to afford β-D-cellobiosyl azide **1h** (30 mg, 82%) as colorless solid. Spectral characteristics of this compound matched the reported data.[2]

$H^1$ NMR (400 MHz, $D_2O$): δ=4.77 (d, *J* = 8.8 Hz, 1H), 4.51 (d, *J* = 8.0 Hz, 1H), 3.65-4.00 (m, 3H), 3.65-3.76 (m, 4H), 3.46-3.53 (m, 2H), 3.41 (t, *J* = 9.2 Hz, 1H), 3.29-3.35 (m, 2H).

**β-*D*-Lactosyl azide (1e)**

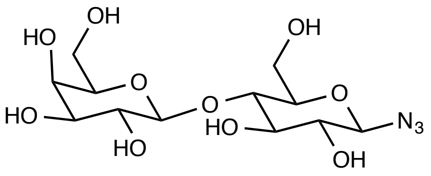

*D*-Lactose monohydrade (36 mg, 0.1 mmol), DMC (85 mg, 0.5 mmol), $NaN_3$ (97 mg, 1.5 mmol), triethylamine (208 μl, 1.5 mmol) in deutrium oxide (500 μl) were shaken for 1 h and lyophilized. To the residue was added $Ac_2O$ (300 μl) and pyridine (1 ml) and the reaction mixture was stirred overnight at room temperature. The reaction was concentrated *in vaccuo* and the product was isolated by reverse phase chromatography (12g of C18 column, 50 ml of $H_2O$, then 100 ml of acetonitrile). The acetonitrile fraction was concentrated *in vacuo* then lyophilized. To the residues was added sodium methoxide (6 mg, 0.1 mmol) and dry methanol. The reaction mixture was stirred overnight under nitrogen atmosphere. The reaction mixture was neutralized with acidic resin (DOWEX® 50WX2), filtered and lyophilized to afford β-*D*-Lactosyl azide (36 mg, 99%) as colorless solid. Spectral characteristics of this compound matched the reported data.[2]

$H^1$ NMR (400 MHz, $D_2O$): δ=4.77 (d, *J* = 8.8 Hz, 1H), 4.44 (d, *J* = 10.4 Hz, 1H), 3.91-4.00 (m, 2H), 3.65-3.84 (m, 8H), 3.54 (m, 1H), 3.31 (m, 1H).

**Fucα1-2Galβ1-3[Fucα1-4]GlcNAcβ1-3Galβ1-4Glc-1$N_3$ (1j)**

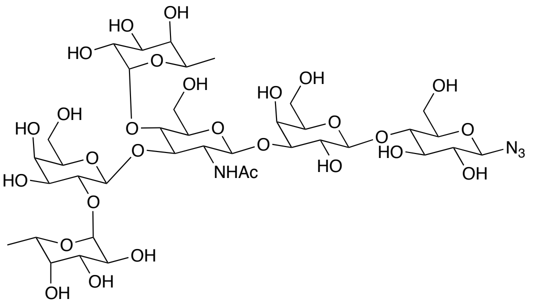

Lacto-*N*-difucohexaose (Fucα1-2Galβ1-3[Fucα1-4]GlcNAcβ1-3Galβ1-4Glc, 5.0 mg, 0.005 mmol), DMC (8.5 mg, 0.05 mmol), $NaN_3$ (8.0 mg, 0.125 mmol), triethylamine (10.4 μl, 0.075 mmol) in deutrium oxide (100 μl) were shaken for 8 h and lyophilized. To the residue was added $Ac_2O$ (50 μl) and pyridine (150 μl) and the reaction mixture was stirred overnight at room temperature. The reaction was concentrated *in vaccuo* and the product was isolated by reverse phase chromatography (1g of C18 column, 5 ml of $H_2O$, then 10 ml of acetonitrile). The acetonitrile fraction was concentrated *in vacuo* then lyophilized. To the residues was added sodium methoxide (2 mg) and dry methanol (100 μl). The reaction mixture was stirred overnight under nitrogen atmosphere. The reaction mixture was neutralized with acidic resin (DOWEX® 50WX2), filtered, washed with water and lyophilized to afford **1j** (4.0 mg, 78%) as colorless solid.

HRMS (MALDI-TOF): calcd. for $C_{41}H_{70}N_4O_{25}Na$ $[M+Na]^+$, 1047.3605. found 1047.3641.

**β–3'-Sialyllactosyl azide (entry 1k)**

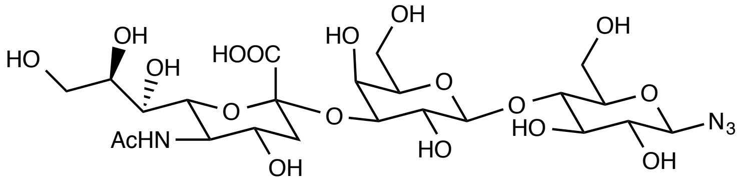


3'-Sialyllactose (Neu5Acβ2-3Galβ1-4Glc, 10.0 mg, 0.015 mmol), DMC (25.5 mg, 0.15 mmol), $NaN_3$ (24 mg, 0.375 mmol), triethylamine (31.2 μl, 0.225 mmol) in deutrium oxide (250 μl) were shaken for 5 h and and lyophilized. To the residue was added $Ac_2O$ (150 μl) and pyridine (450 μl) and the reaction mixture was stirred overnight at room temperature. The reaction was concentrated *in vaccuo* and the product was isolated by reverse phase chromatography (2g of C18 column, 10 ml of $H_2O$, then 20 ml of acetonitrile). The acetonitrile fraction was concentrated *in vacuo* then lyophilized. To the residues was added LiOH (2 mg) and methanol:water (1:1, 100 μl) and stirred overnight. The reaction mixture was neutralized with acidic resin (DOWEX® 50WX2), filtered, washed with water and lyophilized to afford β–3'-Sialyllactosyl azide **1k** (8.0 mg, 80%) as colorless solid.
HRMS (MALDI-TOF): calcd. for $C_{31}H_{51}N_5O_{22}Na[M+Na]^+$, 681.2079. found 681.2071.

**β–6'-Sialyllactosyl azide (entry 1l)**

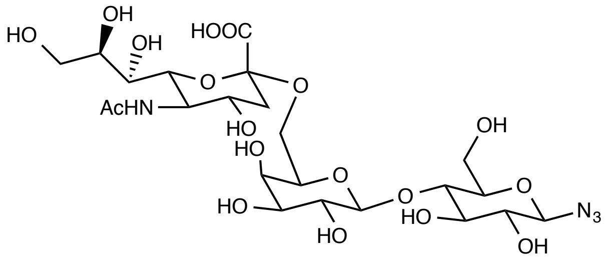


6'-Sialyllactose (Neu5Acβ2-6Galβ1-4Glc, 10.0 mg, 0.015 mmol), DMC (25.5 mg, 0.15 mmol), $NaN_3$ (24 mg, 0.375 mmol), triethylamine (31.2 μl, 0.225 mmol) in deutrium oxide (250 μl) were shaken for 5 h and and lyophilized. To the residue was added $Ac_2O$ (150 μl) and pyridine (450 μl) and the reaction mixture was stirred overnight at room temperature. The reaction was concentrated *in vaccuo* and the product was isolated by reverse phase chromatography (2g of C18 column, 10 ml of $H_2O$, then 20 ml of acetonitrile). The acetonitrile fraction was concentrated *in vacuo* then lyophilized. To the residues was added LiOH (2 mg) and methanol:water (1:1, 100μl) and stirred overnight. The reaction mixture was neutralized with acidic resin (DOWEX® 50WX2), filtered, washed with water and lyophilized to afford β–6'-Sialyllactosyl azide **1l** (9.1 mg, 91%) as colorless solid.
HRMS (MALDI-TOF): calcd. for $C_{31}H_{51}N_5O_{22}Na$ $[M+Na]^+$, 681.2079. found 681.2071.

## 4. Synthesis of glycan-tetrazine probe 3

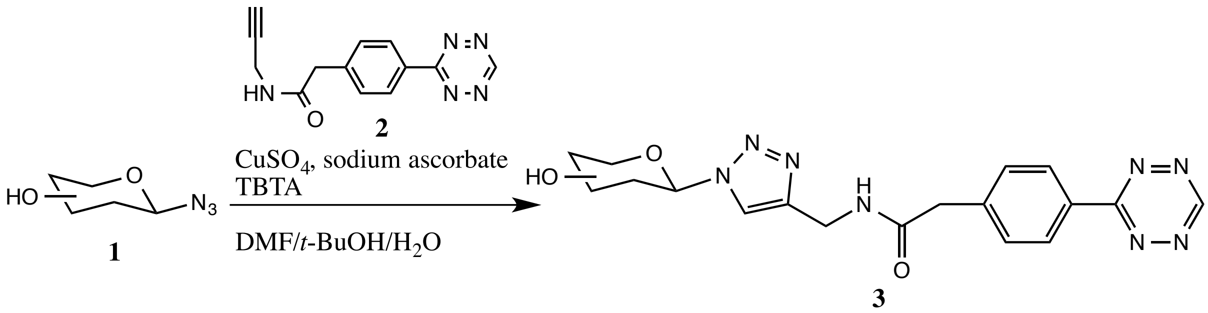


**General procedures**

**Procedure 1** (entry 1-9, Table 1)
Azido glycan **1** (1.0 eq.) and $CuSO_4$ (0.2 eq.) were dissolved in water (40 mM). To this solution at 0 ºC was added a solution of sodium ascorbate (2.0 eq.), TBTA (Tris[(1-benzyl-1,2,3-triazol-4-yl)methyl]amine, 0.2 eq.) in DMF:*t*-BuOH (20mM) followed by the alkyne **2** (1.0 eq.) was added to the reaction mixture and stirred at 0ºC for 5 h. The reaction mixture was directly loaded on a column for reverse phage purification (column: SNAP Cartridge KP-C18-HS 60g, flow rate: 35ml/min with collection based on UV detection) to afford the triazole product 3.

**Procedure 2** (used for smaller scale reaction, entry 10-12, Table 1)
Azido glycan **1** (1.0 eq.) and $CuSO_4$ (2 mg, 12 mM) were dissolved in water (200 μl). To the mixture were added sodium ascorbate (14 mg, 120 mM), TBTA (Tris[(1-benzyl-1,2,3-triazol-4-yl)methyl]amine, 4 mg, 12 mM), DMF (200 μl), *t*-BuOH (200 μl) and the mixture was kept at 0 ºC. Alkyne **3** (9 mg, 60 mM) was added to the reaction mixture and stirred at 0ºC for 5 h and the reaction was lyophilized. The residues were dissolved in water (200 μl) and the precipitates were removed by centrifugation. Purification by HPLC (column: ZORBAX 300SB-C18 column (9.4 x 250 mm), flow rate : 3 ml/min) afforded the triazole product.

**Tetrazine-glucose conjugate 3a**

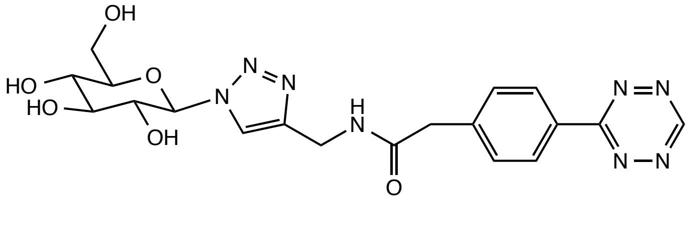

β-*D*-Glucopyranosyl azide **1a** (10 mg, 0.1 mmol) was used following procedure 1. Reverse phase purification (eluent: acetonitrile/water=1/4) afforded conjugate **3a** (18 mg, 80%) as red solid.
$H^1$ NMR (400 MHz, MeOD): δ=10.32 (s, 1H), 8.53 (d, *J* = 8.4 Hz, 2H), 8.04 (s, 1H), 7.57 (d, *J* = 8.4 Hz, 2H), 5.59 (d, *J* = 8.8 Hz, 1H), 4.50 (s, 2H), 3.3-3.9 (m, 8H); $C^{13}$ NMR (100 MHz, MeOD): δ=173.2, 167.6, 159.2, 142.2, 133.4, 132.1, 131.4, 131.3, 129.3, 89.5, 81.1, 78.4,

74.0, 70.9, 62.4, 43.5, 35.9. HRMS (MALDI-TOF): calcd. for $C_{19}H_{22}N_8O_6Na$ $[M+Na]^+$, 481.1560. found 481.1553.

**Tetrazine-mannose conjugate 3b**

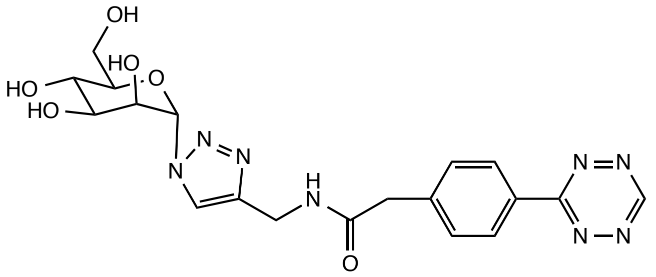

α-*D*-Mannopyranosyl azide **1b** (20 mg, 0.1 mmol) was used following procedure 1. Reverse phase purification (eluent: acetonitrile/water=1/4) afforded conjugate **3b** (35 mg, 76%) as red solid.

$H^1$ NMR (400 MHz, $D_2O$): δ=10.20 (s, 1H), 8.24 (d, *J* = 7.6 Hz, 2H), 7.83 (s, 1H), 7.39 (d, *J* = 7.6 Hz, 2H), 5.89 (d, *J* = 2.4 Hz, 1H), 4.37 (s, 2H), 3.93 (dd, *J* = 3.6 Hz, 8.8 Hz, 1H), 3.58-3.65 (m, 6H), 3.05-3.09 (m, 1H) ; $C^{13}$ NMR (100 MHz, $D_2O$): δ=173.8, 166.3, 157.3, 140.3, 130.5, 130.2, 129.7, 128.6, 86.6, 76.0, 70.5, 68.2, 66.4, 60.4, 42.2, 34.6; HRMS (MALDI-TOF): calcd. for $C_{19}H_{22}N_8O_6Na$ $[M+Na]^+$, 481.1560. found 481.1553.

**Tetrazine-galactose conjugate 3c**

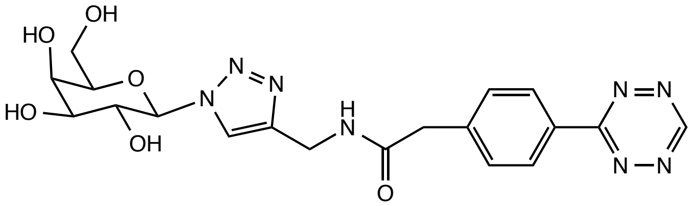

β-*D*-Galactopyranosyl azide **1c** (10 mg, 0.05 mmol) was used following procedure 1. Reverse phase purification (eluent: acetonitrile/water=1/4) afforded conjugate **3c** (11 mg, 50%) as red solid.

$H^1$ NMR (500 MHz, $D_2O$): δ=10.31 (s, 1H), 8.35 (broad s, 2H), 8.08 (s, 1H), 7.42 (broad s, 2H), 5.61 (d, *J* = 9.0 Hz, 1H), 4.49 (s, 2H), 4.13 (dd, *J* = 9.0 Hz, 9.0 Hz, 1H), 4.04 (d, *J* = 3 Hz, 1H), 3.94 (dd, *J* = 5.5 Hz, 6.0 Hz, 1H), 3.82 (dd, *J* = 3.0 Hz, 9.0 Hz, 1H), 3.72-3.73 (m, 4H) ; $C^{13}$ NMR (125 MHz, $D_2O$): δ=173.9, 166.3, 157.3, 140.3, 132.7, 130.2, 129.8, 129.6, 128.5, 88.0, 78.2, 72.9, 69.9, 68.5, 60.8, 42.1, 34.7; HRMS (MALDI-TOF): calcd. for $C_{19}H_{22}N_8O_6Na$ $[M+Na]^+$, 481.1560. found 481.1578.

**Tetrazine-fucose conjugate 3d**

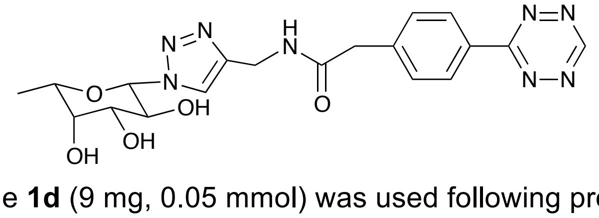

β-L-Fucopyranosyl azide **1d** (9 mg, 0.05 mmol) was used following procedure 1. Reverse phase purification (eluent: acetonitrile/water=1/4) afforded conjugate **3d** (15 mg, 72%) as red solid.

$H^1$ NMR (400 MHz, $D_2O$): δ=10.33 (s, 1H), 8.34 (d, *J* = 7.6 Hz, 2H), 8.12 (s, 1H), 7.51 (d, *J* = 7.6 Hz, 2H), 5.62 (d, *J* = 8.8 Hz, 1H), 4.52 (s, 2H), 4.13 (dd, *J* = 8.8 Hz, 9.6 Hz, 1H), 4.06 (q, *J* = 6.0 Hz, 1H), 3.89 (d, *J* = 3.2 Hz, 1H), 3.84 (dd, *J* = 3.2 Hz, 9.6 Hz, 1H), 3.75 (s, 2H), 1.25 (d, *J* = 6.0, 3H); $C^{13}$ NMR (100 MHz, $D_2O$): δ=173.8, 166.2, 157.3, 140.3, 132.7, 130.2, 129.8, 129.7, 128.5, 87.9, 74.3, 73.0, 71.1, 69.4, 42.1, 34.5, 15.4. HRMS (MALDI-TOF): calcd. for $C_{19}H_{22}N_8O_5Na$ $[M+Na]^+$, 465.1611. found 465.1624.

**Tetrazine-*N*-actyl glucosamine conjugate 3e**

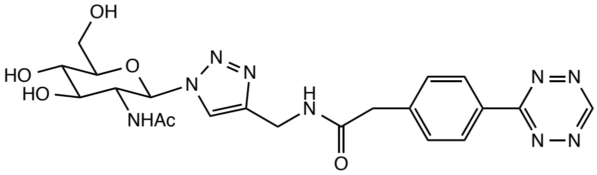


2-Acetamido-2-deoxy-β-D-glucopyranosyl azide **1e** (13 mg, 0.05 mmol) was used following procedure 1. Reverse phase purification (eluent: acetonitrile/water=1/4) afforded conjugate **3e** (18 mg, 70%) as red solid.

$H^1$ NMR (500 MHz, $D_2O$): δ=10.36 (s, 1H), 8.45 (d, *J* = 8.5 Hz, 2H), 7.96 (s, 1H), 7.58 (d, *J* = 8.5 Hz, 2H), 5.78 (d, *J* = 9.5 Hz, 1H), 4.50 (s, 2H), 4.17 (dd, *J* = 10.0 Hz, 10.0 Hz, 1H), 3.90 (dd, *J* = 2.0 Hz, 12.5 Hz, 1H), 3.70-3.81 (m, 5H), 3.64 (dd, *J* = 9.0 Hz, 9.5 Hz, 1H), 1.76 (s, 3H); $C^{13}$ NMR (100 MHz, $D_2O$): δ=174.4, 173.8, 166.4, 157.3, 140.4, 130.2, 128.6, 86.3, 78.8, 73.4, 69.2, 60.3, 55.3, 42.2, 34.5, 21.5; HRMS (MALDI-TOF): calcd. for $C_{21}H_{25}N_9O_6Na$ $[M+Na]^+$, 522.1826. found 522.1863.

**Tetrazine-glucoronic acid conjugate 3f.**

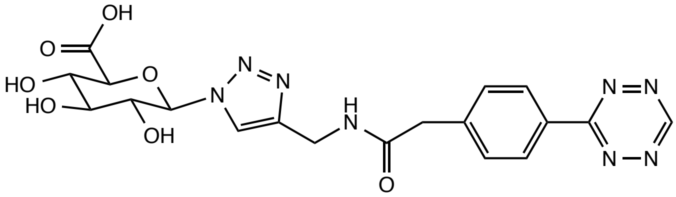


β-*D*-Glucopyranuronic azide **1f** (11 mg, 0.05 mmol) was used following procedure 1. Reverse phase purification (eluent: acetonitrile/water=1/4) conjugate **3f** (14 mg, 57%) as red solid.

$H^1$ NMR (400 MHz, $D_2O$): δ=10.35 (s, 1H), 8.42 (broad s, 2H), 7.99 (s, 1H), 7.56 (broad s, 2H), 5.74 (d, *J* = 9.2 Hz, 1H), 4.49 (s, 2H), 4.18 (m, 1H), 3.95 (dd, *J* = 9.2 Hz, 9.2 Hz, 1H), 3.77 (s, 2H), 3.70 (m, 2H); $C^{13}$ NMR (100 MHz, $D_2O$): δ=173.9, 166.4, 157.3, 140.3, 132.7, 130.3, 130.2, 129.9, 128.6, 87.0, 75.5, 75.4, 71.8, 70.9, 42.3, 34.7 ; HRMS (MALDI-TOF): calcd. for $C_{19}H_{20}N_8O_7Na$ $[M+Na]^+$, 495.1353. found 495.1394.

**Tetrazine-maltose conjugate 3g**

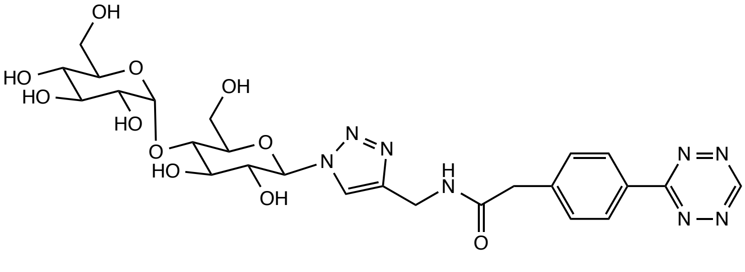

β-*D*-Maltosyl azide **1g** (13 mg, 0.04 mmol) was used following procedure 1. Reverse phase purification (eluent: acetonitrile/water=1/4) afforded conjugate **3g** (17 mg, 76%) as red solid. $H^1$ NMR (500 MHz, $D_2O$): δ=10.37 (s, 1H), 8.46 (d, *J* = 8.5, 2H), 7.98 (s, 1H), 7.59 (d, *J* = 8.5, 2H), 5.71 (d, *J* = 9 Hz, 1H), 5.44 (d, *J* = 3.5 Hz, 1 H), 4.53 (s, 2H), 3.68-3.95 (m, 12H), 3.60 (dd, *J* = 3.5 Hz, 10.0 Hz, 1H), 3.43 (dd, *J* = 9.5 Hz, 9.5 Hz, 1H), 5.74 (d, *J* = 9.2 Hz, 1H), 4.49 (s, 2H), 4.18 (m, 1H), 3.95 (dd, *J* = 9.2 Hz, 9.2 Hz, 1H), 3.77 (s, 2H), 3.70 (m, 2H) ; $C^{13}$ NMR (125 MHz, $D_2O$): δ=174.0, 166.4, 157.3, 140.4, 130.3, 1302, 130.2, 128.6, 99.6, 87.2, 77.4, 76.2, 75.8, 72.8, 72.7, 72.1, 71.6, 69.3, 60.4, 60.3, 42.2, 34.7 ; HRMS (MALDI-TOF): calcd. for $C_{25}H_{32}N_8O_{11}Na$ $[M+Na]^+$, 643.2089. found 643.2122.

**Tetrazine-cellobiose conjugate 3h**

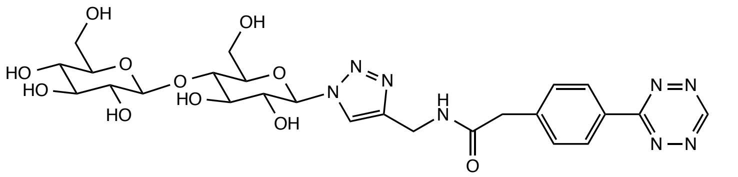

β-*D*-Cellobiosyl azide **1h** (20 mg, 0.05 mmol) was used used following procedure 1. Reverse phase purification (eluent: acetonitrile/water=1/4) afforded conjugate **3h** (24 mg, 72%) as red solid.

$H^1$ NMR (500 MHz, $D_2O$): δ=10.36 (s, 1H), 8.42 (d, *J* = 8.0, 2H), 8.03 (s, 1H), 7.56 (d, *J* = 8.0, 2H), 5.73 (d, *J* = 9.5 Hz, 1H), 4.44 (d, *J* = 7.5 Hz, 1 H), 4.53 (s, 2H), 3.72-3.99 (m, 12H), 3.53 (dd, *J* = 9.0 Hz, 9.0 Hz, 1H), 3.43 (dd, *J* = 9.0 Hz, 10.0 Hz, 1H);

$C^{13}$ NMR (125 MHz, $D_2O$): δ=173.9, 166.3, 157.3, 140.3, 130.3, 1302, 128.6, 102.5, 87.2, 77.6, 77.6, 76.0, 75.4, 74.4, 73.1, 72.0, 69.4, 60.5, 59.6, 42.2, 34.6; HRMS (MALDI-TOF): calcd. for $C_{25}H_{32}N_8O_{11}Na$ $[M+Na]^+$, 643.2089. found 643.2072.

**Tetrazine-lactose conjugate 3i**

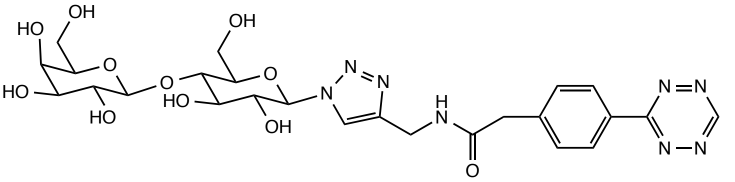

β-D-Lactosyl azide (22 mg, 0.06 mmol) was used used following procedure 1. Reverse phase purification (eluent: acetonitrile/water=1/4) afforded conjugate **3i** (24 mg, 64%) as red solid.

$H^1$ NMR (400 MHz, $D_2O$): δ=10.30 (s, 1H), 8.34 (d, *J* = 7.2 Hz, 2H), 8.05 (s, 1H), 7.49 (d, *J* = 7.2 Hz, 2H), 5.70 (d, *J* = 6.4 Hz, 1H), 4.45-4.48 (m, 3H), 3.51-3.97 (m, 14H); $C^{13}$ NMR (100 MHz, $D_2O$): δ=173.8, 166.2, 157.3, 140.3, 132.7, 130.2, 129.9, 129.7, 128.5, 102.9, 87.2,

77.6, 77.3, 75.4, 74.5, 72.5, 71.9, 71.0, 68.5, 61.0, 59.7, 42.2, 34.6; HRMS (MALDI-TOF): calcd. for $C_{25}H_{32}N_8O_{11}Na$ $[M+Na]^+$, 643.2089. found 643.2037.

**Tetrazine Fucα1-2Galβ1-3[Fucα1-4]GlcNAcβ1-3Galβ1-4Glc conjugate 3j**

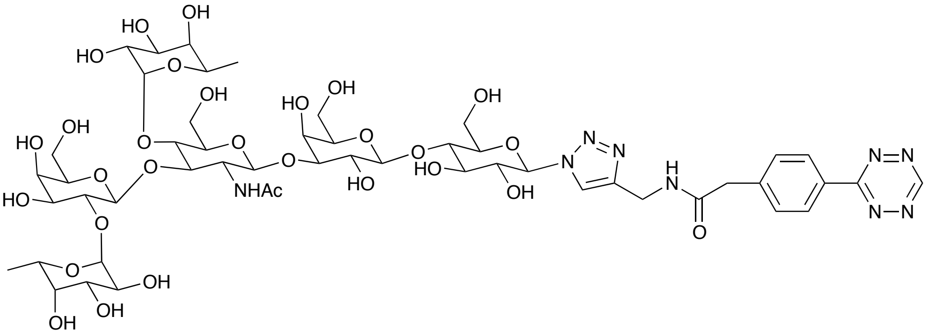

β-lacto-*N*-difucohexaosy azide **1j** (4.0 mg, 0.0039 mmol) was used following procedure 2. HPLC purification (eluent: acetonitrile/water=1/4) afforded conjugate **3j** (1.0 mg, 20%) as red solid.

HRMS (MALDI-TOF): calcd. for $C_{51}H_{76}N_9O_{29}$ $[M+H]^+$, 1278.4750. found 1278.4632.

**Tetrazine-β-3'-sialyllactose conjugate 3k**

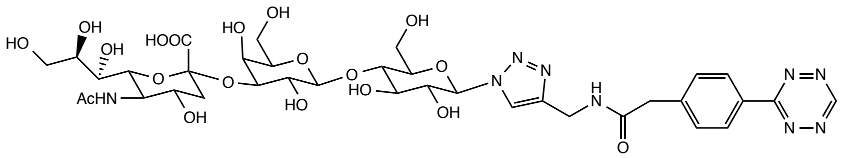

β–3'-sialyllactosyl azide **1k** (3.4 mg, 0.0052 mmol) was used as starting suger-azide in **General procedure2**. HPLC purification (eluent: acetonitrile/water=1/4) gave 2-(4-(1,2,4,5-tetrazin-3-yl)phenyl)-*N*-(β–3'-sialyllactosyl-1,2,3-triazol-4-yl)methyl)acetamide (1.4 mg, 30%) as red solid.

HRMS (MALDI-TOF): calcd. for $C_{36}H_{49}N_9O_{19}Na$ $[M+Na]^+$, 934.3043. found 934.3011.

**Tetrazine-β-6'-sialyllactose conjugate 3l**

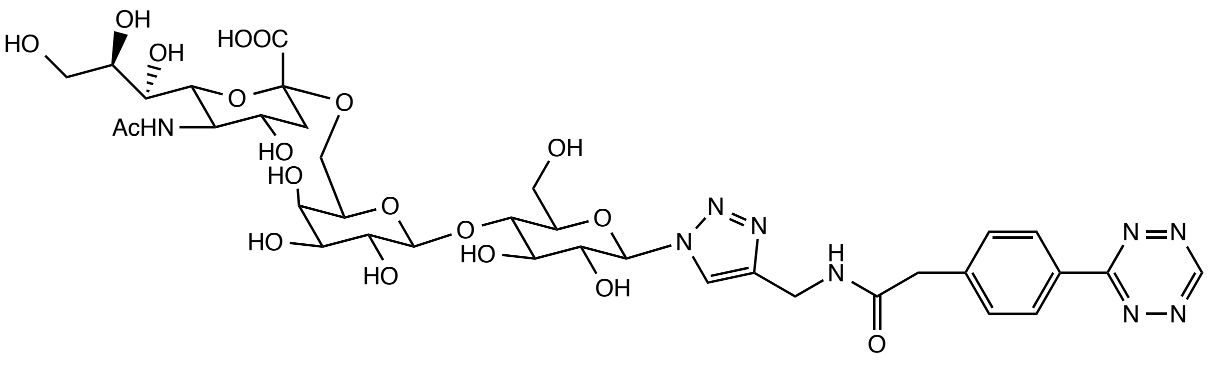

β–6’-sialyllactosyl azide **1l** (9.1 mg, 0.014 mmol) was used according to procedure 2. HPLC purification (eluent: acetonitrile/water=1/4) afforded conjugate **3l** (3.5 mg, 17%) as red solid. HRMS (MALDI-TOF): calcd. for $C_{36}H_{49}N_9O_{19}Na$ $[M+Na]^+$, 934.3043. found 934.3045.

## 5. Synthesis of 3-GluNAc-Cy3

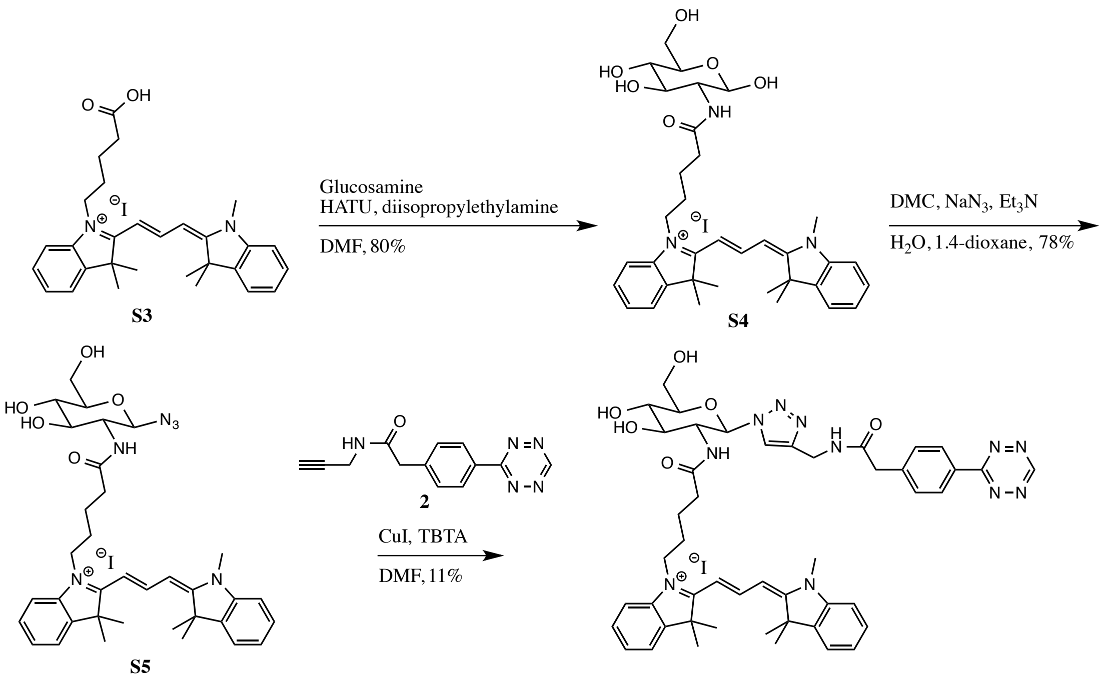


### *N*-CY3-Glucosamine (S4)

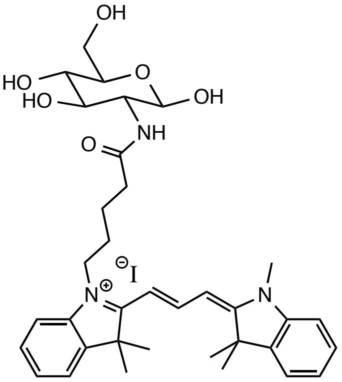

S4

To the mixture C33-COOH **S3** (41 mg, 0.072 mmol) and 2.6-lutidine (10 μl, 0.087 mmol) in dry DMF (0.5 ml) was added HATU (33 mg, 0.087 mmol) at 0 ºC and reaction mixture was stirred for 5 min. Glucosamine hydrochloride (23 mg, 0.107 mmol) was added and the reaction was stirred for 1 h at 0 ºC. The mixture was directly loaded on a reverse phase column for purification (eluent: acetonitrile/water=3/7) to afford **S4** (42 mg, 80%) as red solid.

$H^1$ NMR (400 MHz, MeOD): δ=8.46 (td, *J* = 13.6 Hz, 5.6 Hz, 1H), 7.46 (d, *J* = 7.2 Hz, 2H), 7.36 (m, 2H), 7.24 (m, 4H), 6.38 (m, 2H), 5.02 (d, *J* = 3.2 Hz, 0.7H for α isomer), 4.50 (d, *J* = 8.0 Hz, 0.3 H for β isomer); , 4.09 (m, 2H), 3.65 (m, 8H), 3.27 (m, 1H), 2.30 (m, 2H), 1.75 (m, 16H); $C^{13}$ NMR (100 MHz, MeOD): δ=176.9, 176.1, 175.9, 152.3, 144.2, 143.2, 142.3, 142.2, 130.2, 130.1, 126.9, 126.8, 123.6, 123.5, 112.5, 112.4, 104.0, 103.8, 92.7, 78.2, 76.2, 73.2, 72.7, 72.4, 62.9, 58.8, 56.0, 50.7, 45.1, 36.2, 31.9, 28.4, 28.3, 27.8, 24.1. HRMS (MALDI-TOF): calcd. for $C_{35}H_{46}N_3O_6$ $[M-I]^+$, 604.3381. found 604.3327.

## *N*-CY3-Glucosamine-azide (S5)

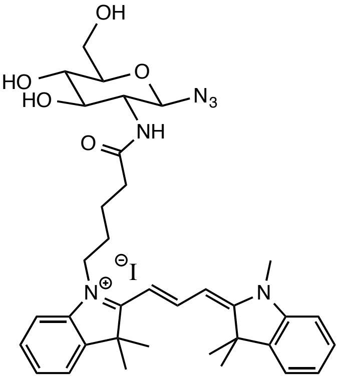


**S5**

Compound **S4** (30 mg, 0.041 mmol), $NaN_3$ (24 mg, 0.37 mmol) and triethylamine (51 μl, 0.37 mmol) in water (125 μl) and 1.4-dioxane (125 μl) were treated with DMC (35 mg, 0.21 mmol) and the reaction mixture was shaken for 1 h. The crude mixture was directly loaded on a reverse phase column (eluent: acetonitrile/water=3/7) to afford **S5** (24 mg, 78%) as red solid.

$H^1$ NMR (400 MHz, MeOD): δ=8.56 (t, *J* = 13.6 Hz, 1H), 7.55 (d, *J* = 7.2 Hz, 2H), 7.46 (m, 2H), 7.34 (m, 4H), 6.47 (d, *J* = 13.6 Hz, 2H), 4.45 (d, *J* = 9.6 Hz, 1 H), 4.19 (m, 2H), 3.88 (d, *J* = 12.0 Hz, 1H), 3.71 (m, 5H), 3.46 (t, *J* = 8.0 Hz, 1H), 3.33 (m, 2H), 2.38 (m, 2H), 1.85 (m, 16H); $C^{13}$ NMR (100 MHz, MeOD): δ=176.7, 176.0, 175.9, 152.1, 144.1, 143.3, 142.2, 142.1, 130.0, 129.9, 126.8, 126.7, 123.5, 123.3, 112.4, 112.2, 103.8, 103.7, 90.2, 80.4, 75.7, 71.8, 62.5, 56.5, 50.6, 50.6, 44.9, 36.5, 31.8, 28.3, 28.3, 28.1, 27.6, 24.1. HRMS (MALDI-TOF): calcd. for $C_{35}H_{45}N_6O_5$ $[M-I]^+$, 629.3446. found 629.3441.

## 3-GluNAc-Cy3

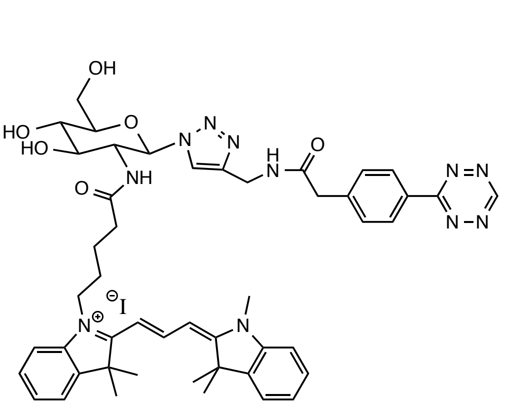

Compound **S5** (24 mg, 0.03 mmol), CuI (12 mg, 0.06 mmol), TBTA (2 mg, 0.003 mmol) were taken up in DMF (1 ml) at 0 °C and alkyne **3** (11 mg, 0.04 mmol) was added. The reaction was stirred for 5 h at 0 °C and the crude reaction mixture was loaded directly on a reverse phase column (eluent: acetonitrile/water=2/3) to afford **3-GluNAc-Cy3** (4 mg, 11%) as red solid.

$H^1$ NMR (400 MHz, MeOD): δ=10.29 (s, 1H), 8.52 (m, 3H), 8.07 (s, 1H), 7.57 (d, *J* = 8.0 Hz, 2H), 7.52 (m, 2H), 7.42 (m, 2H), 7.29 (m, 4H), 6.45 (m, 2H), 5.77 (d, *J* = 10.0 Hz, 1H), 4.44 (m, 2H), 4.20 (t, *J* = 10.0 Hz, 1H), 4.00 (m, 2H), 3.90 (m, 1H), 3.75 (m, 1H), 3.69 (m, 4H), 3.62 (s, 2H), 3.52 (m, 3H), 2.15 (m, 2H), 1.77 (m, 12H), 1.58 (m, 4H); $C^{13}$ NMR (125 MHz, MeOD): δ=176.5, 175.8, 175.5, 172.8, 167.4, 159.2, 152.0, 146.3, 144.0, 143.3, 142.3, 142.1, 142,0, 132.0, 131.3, 129.9, 129.9, 129.2, 126.7, 126.6, 123.4, 123.3, 122.8, 112.4, 112.2, 104.0, 103.9, 88.2, 81.3, 75.5, 71.4, 62.2, 56.8, 50.6, 50.5, 44.8, 43.5, 37.7, 36.3, 35.9, 31.7, 28.3, 28.3, 28.1, 27.5, 23.9; HRMS (MALDI-TOF): calcd. for $C_{48}H_{56}N_{11}O_6$ $[M-I]^+$, 882.4410. found 882.4410.

# 6. Synthesis of *trans*-cyclooctene derivative S7 for kinetic study

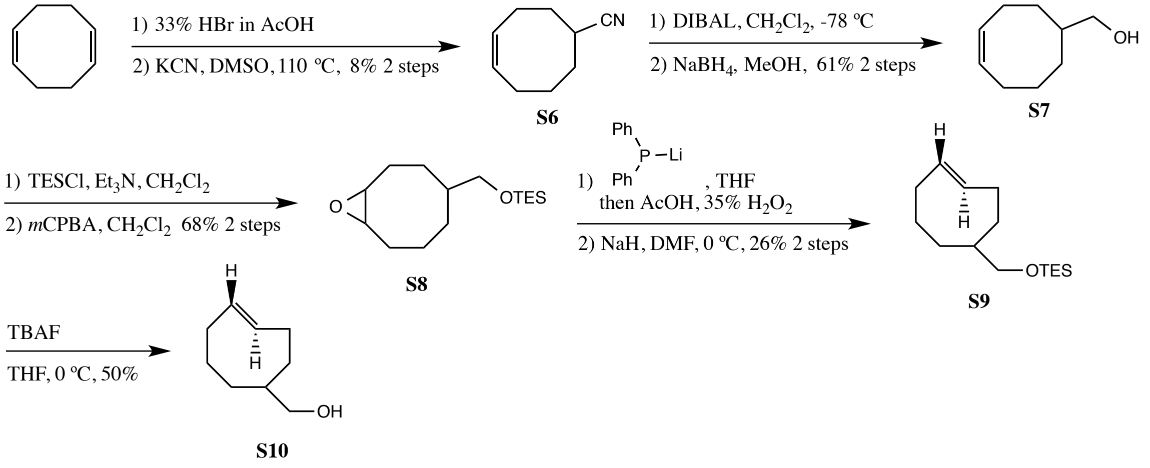


## (*Z*)-cyclooct-4-en-1-ylmethanol (S7)

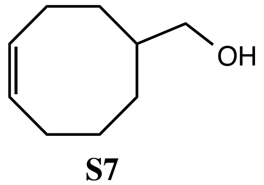


**S7**

Compound **S6**[5] (1.1 g, 8.15 mmol) in $CH_2Cl_2$ (33 ml) was treated dropwise with diisobutylalminium hydride (1.0 M in toluene, 12.2 ml, 12.2 mmol) at -78 °C and stirred for 1 h under nitrogen atmosphere. The reaction mixture was warmed up to 0 °C and 2 M HCl (20 ml) was added dropwise to the reaction mixture. The mixture was stirred for another 30 min at room temperature then the organic compounds were extracted with ethyl acetate, washed with brine, dried over $Na_2SO_4$, filtrated and concentrated *in vacuo.* The residue were taken up

in MeOH (33 ml) at room temperature and treated with sodium borohydride (620 mg, 16.3 mmol). After 1 h, the reaction was quenched with saturated $NH_4Cl$ and the organic compounds were extracted with ethyl acetate, washed with brine, dried over $Na_2SO_4$, filtrated and concentrated *in vacuo*. Purification by silica gel column chromatography (pentane : ethyl acetate = 10 : 1) afforded **S7** (690 mg, 61%) as colorless oil.
$H^1$ NMR (400 MHz, $CDCl_3$): δ=5.63-5.67 (m, 2H), 3.35-3.44 (m, 2H), 2.07-2.35 (m, 4H), 1.51-1.72 (m, 4H), 1.30-1.39 (m, 2H), 1.09-1.18 (m, 1H); $C^{13}$ NMR (100 MHz, $CDCl_3$): δ=130.2, 130.1, 69.2, 40.5, 31.7, 29.6, 28.1, 25.9, 24.6.

## Epoxide S8 (S8)

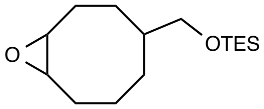


**S8**

Compound **S7** (358 mg, 2.56 mmol) dissolved in $CH_2Cl_2$ (5 ml) at room temperature was treated with triethylamine (534 μl, 3.84 mmol) and chlorotriethylsilane (645 μl, 3.84 mmol). The reaction mixture was stirred for 1 h then quenched with saturated $NaHCO_3$ and the organic compounds were extracted with ethyl acetate, washed with brine, dried over $Na_2SO_4$, filtrated and concentrated *in vacuo*. The residue were taken back up in $CH_2Cl_2$ (5 ml) at 0 °C and treated with *m*-chloroperbenzoic acid (<70%, 1.28 g, 5.12 mmol). The reaction mixture was stirred for 2 h at 0 °C, quenched with saturated $Na_2S_2O_3$ and $NaHCO_3$ and the organic compounds were extracted with ethyl acetate, washed with brine, dried over $Na_2SO_4$, filtrated and concentrated *in vacuo*. Purification by silica gel column chromatography (pentane : ethyl acetate = 20 : 1) gave **S8** (471 mg, 68%, mixture of diasteromer) as colorless oil.
$H^1$ NMR (400 MHz, $CDCl_3$): δ=3.22-3.31 (m, 2H), 2.77-2.91 (m, 2H), 2.12-2.26 (m, 1H), 1.96-2.06 (m, 1H), 1.46-1.85 (m, 4H), 1.06-1.37 (m, 4H), 0.89 (t, *J* = 8.0 Hz, 9H), 0.52 (q, *J* = 8.0 Hz, 6H); $C^{13}$ NMR (100 MHz, $CDCl_3$): δ=68.9, 55.9, 55.3, 41.1, 30.3, 29.4, 28.0, 25.8, 23.4, 6.82, 4.39.

## (*E*)-(cyclooct-4-en-1-ylmethoxy)triethylsilane (S9)

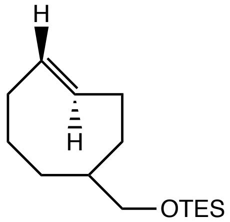


**S9**

According to the previously reported procedure,[7] to chlorodiphenylphosphine (723 μl, 4.0 mmol) in dry THF (5 ml) was slowly added lithium wire (62 mg, 8.8 mmol) washed in dry THF (5 ml). The mixture was stirred vigorously at room temperature overnight to give dark red lithium diphenylphosphide solution. This solution was added to **S8** (471 mg, 1.74 mmol) dissolved in a few drops of THF and the reaction mixture was stirred for 2 d at room

temperature (the color changed to pale yellow). The reaction mixture was cooled to 0 ºC and AcOH (251 μl, 4.4 mmol) followed by 35% $H_2O_2$ (466 μl, 4.8 mmol) were slowly added to the stirring for 1 h at room temperature. The organic compounds were extracted with $CH_2Cl_2$, washed with brine, dried over $Na_2SO_4$, filtrated and concentrated *in vacuo*. To the residue dissolved in DMF (10 ml) were added to NaH (<60%, 209 mg, 5.22 mmol, washed with dry hexane before using) and stirred for 1 h at room temperature. The reaction was quenched with saturated $NH_4Cl$ and the organic compounds were extracted with ethyl acetate, washed with brine, dried over $Na_2SO_4$, filtrated and concentrated *in vacuo*. Purification by silica gel column chromatography (pentane : ethyl acetate = 10 : 1) gave **S9** (113 mg, 26%, mixture of atropisomers) as colorless oil.
$H^1$ NMR (400 MHz, $CDCl_3$): δ=5.55-5.74 (m, 2H), 3.77-3.85 (m, 1H), 1.45-2.36 (m, 12H) ; $C^{13}$ NMR (100 MHz, $CDCl_3$): δ=134.9, 133.3, 68.4, 45.0, 38.2, 35.5, 35.0, 33.9, 32.8, 6.78, 4.05.

### (*E*)-cyclooct-4-en-1-ylmethanol (S10)

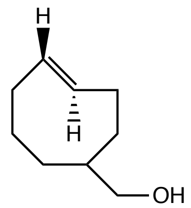


**S10**

To the mixture of **S9** (20 mg, 0.08 mmol) in THF (1 ml) was added tetrabuthylammonium fluoride trihydrate (37 mg, 0.12 mmol) at 0 ºC and reaction mixture was stirred for 1 h at 0 ºC. Water was added to the reaction mixture and the organic compounds were extracted with ethyl acetate, washed with brine, dried over $Na_2SO_4$, filtrated and concentrated *in vacuo*. Purification by silica gel column chromotography (pentane : ethyl acetate = 10 : 1) gave **S10** (5 mg, 50%, mixture of atropisomers) as colorless oil.
$H^1$ NMR (400 MHz, $CDCl_3$): δ=5.49-5.69 (m, 2H), 3.33-3.46 (m, 1H), 1.18-2.44 (m, 12H) ; $C^{13}$ NMR (125 MHz, $CDCl_3$): δ=134.6, 133.5, 69.3, 45.0, 38.2, 35.4, 35.0, 33.9, 32.9.

## 7. Kinetic study of tetrazine-TCO cycloaddition

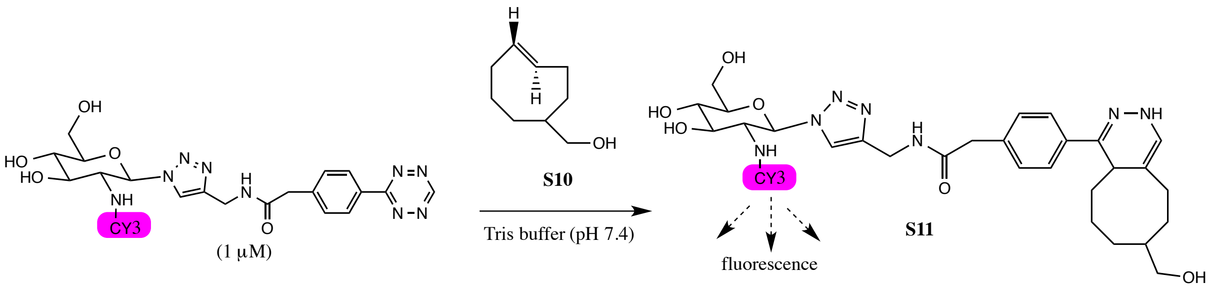


Kinetic studies of tetrazine-*trans*-cyclooctene (TCO) cycloaddition were performed by monitoring the change in CY3 fluorescence resulting from the cycloaddition[8] (**Figure SI1**). Stock solutions of **3-GluNAc-Cy3** and TCO in DMSO (1 mM) were diluted in Tris buffer pH 7.4 to final concentration of 1.0 μM. Reactions were performed with 1.0 and 10 eq of TCO

**S7** while monitoring Cy-3 fluorescence (blue and red curve respectively line, curve are the average of triplicates). As a control, the same reaction was performed in the absence of TCO **S7**. From the observed fluorescent intensity, 1/[sub] values were plotted (**Figure SI2**). Second order constant ($k_2$, $M^{-1}s^{-1}$) was calculated from the slope of the line from 1/[sub] value ($k_2$=8649 $M^{-1}s^{-1}$).

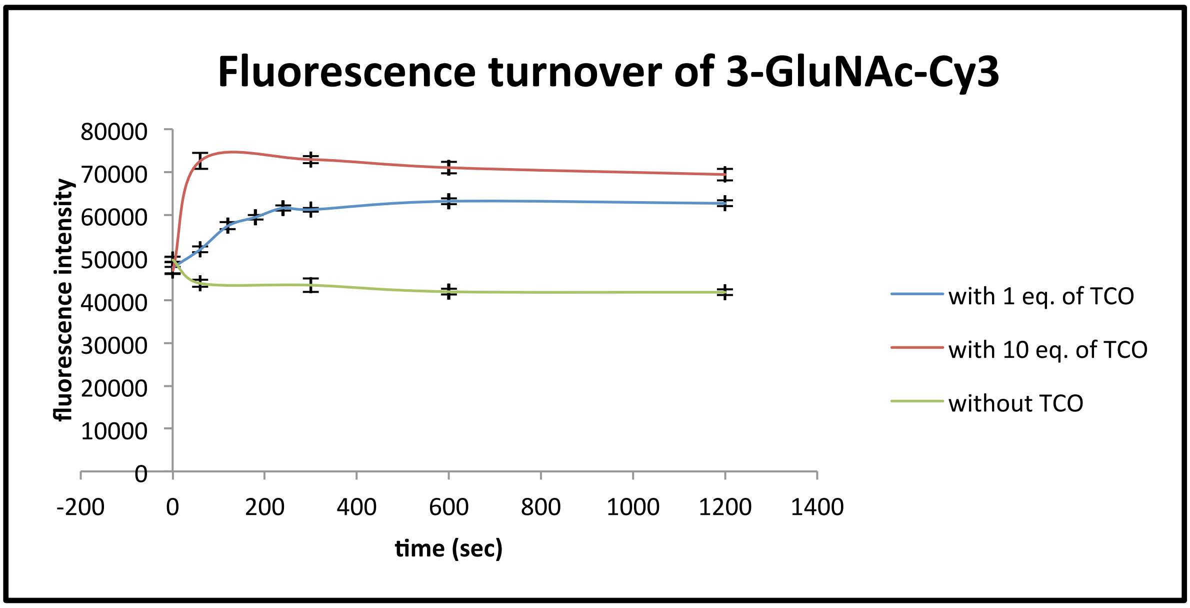


**Figure SI1**: Observation value of fluorescent intensity of tetrazine-CY3 at 570 nm.

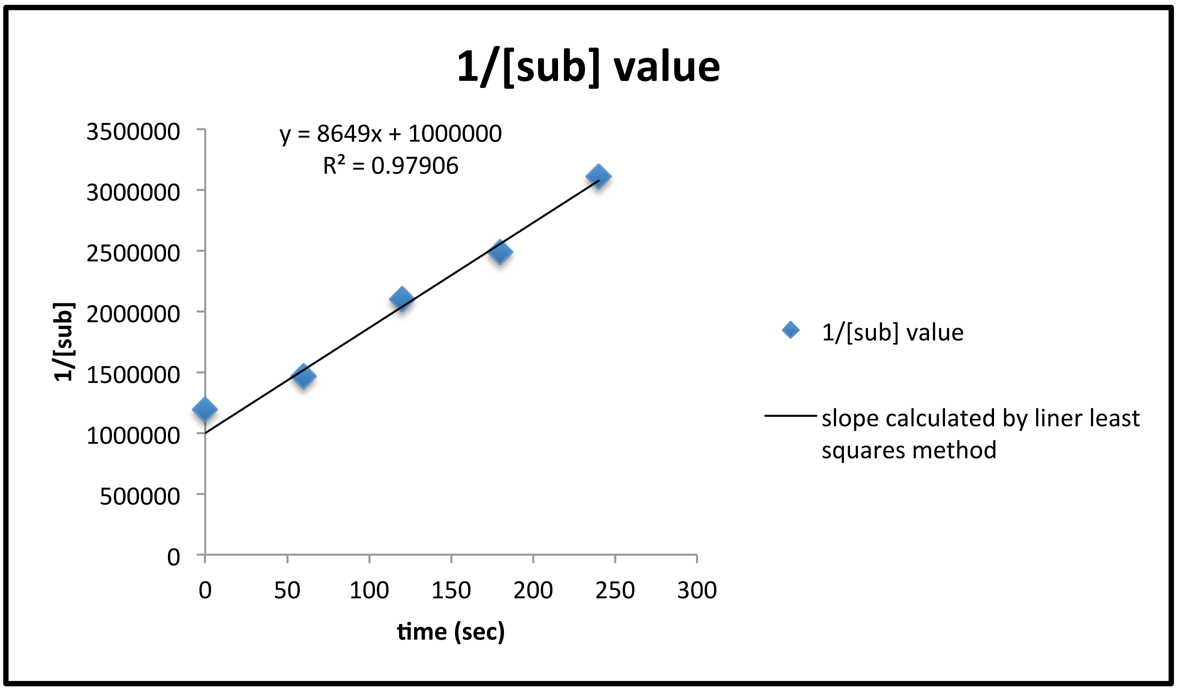


**Supplymentaly figure2**: Slope of 1/[sub] value from the fluorescence intensity.

## 8. Protein conjugation with purified *sf*GFP-TCOK

Purified sfGFP-TCOK and sfGFP-BocK were obtained according to published protocols.[8] The mixture of sfGFP-TCOK (13.5 μM in Tris buffer pH 7.4, 5 μl) and **3-GluNAc-Cy3** (1 mM in DMSO, 0.68 μl) was incubated for 12 h at 37 ºC. The mixture of sfGFP-BocK (13.5 μM in Tris buffer pH 7.4, 10 μl) and **3-GluNAc-Cy3** (1 mM in DMSO, 1.36 μl) were also incubated as control experiment. Each sample was mixed with NuPAGE lithum dodecyl sulfate (LDS) sample buffer and analyzed by 4-12 % SDS-PAGE. The gel was scanned with Ettan DIGE Imager to visualize in-gel flurorescence and stained by silver-staining. The completion of conjugation was assayed by MALDI-TOF sass spectroscopy.

## 9. Protein conjugation with purified *sf*GFP-TCOK

Purified sfGFP-BCNK were prepared according to published protocols.[8]
To the solution of sfGFP-BCNK in Tris buffer pH 7.4 (13.5, 1, 0.1 μM, respectively) was added tetrazine-glycan at 10 equivalents (entry 1) or 5 equivalents (entry 2-4) and the solution was incubated for 10 min at room temperature with gentle agitation on an orbital shaker. The reaction was quenched by addition of 100 eq. of TCO-OH **S10** and the product was analyzed by MALDI-TOF. The same procedure was performed for azide-glycan (entry 5) for 10 min and 3 hours and the reaction was quenched by addition of 100 eq. of tetrazine **2** instead of TCO-OH **S10**.

## 10. Protein modification of *E. coli* expressing *sf*GFP-TCOK

*E. coli* pellet (50 μl) expressing sfGFP-TCOK or sfGFP-BocK were obtained as previously described from 3 mL of culture.[8] The pellets were washed 3x with PBS to remove excess TCOK or BOCK. An aliquot of each *E. coli* pellet (15 μl) were treated with 100 μl of a 25 μM solution of **3-GluNAc-Cy3** in PBS buffer and incubated for 8h at room temperature with gentle agitation on an orbital shaker. The *E. coli* were re-pelleted by centrifugation and washed twice with PBS (centrifuge and discard supernatant). Then, 30 μl of LDS buffer was added to the pellet and the mixture was incubated for 10 min at room temperature. The mixture was analyzed by 4-12 % SDS-PAGE. The gel was characterized by in-gel fluorescence imaging and silver-staining.

## 10. MALDI analysis of Glycoconjugation

GFP-TCOK glycoconjugation (figure 3)

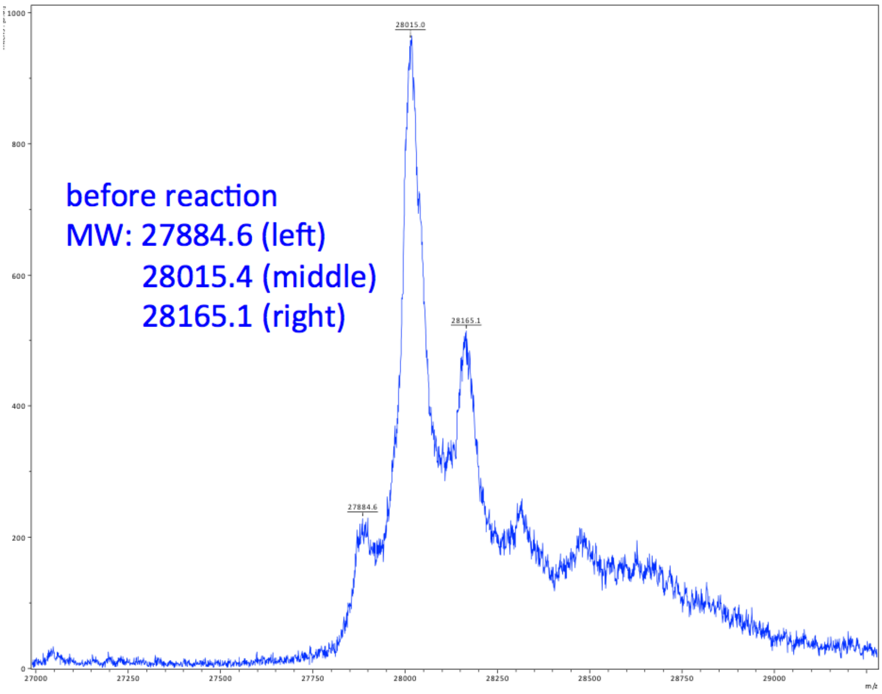


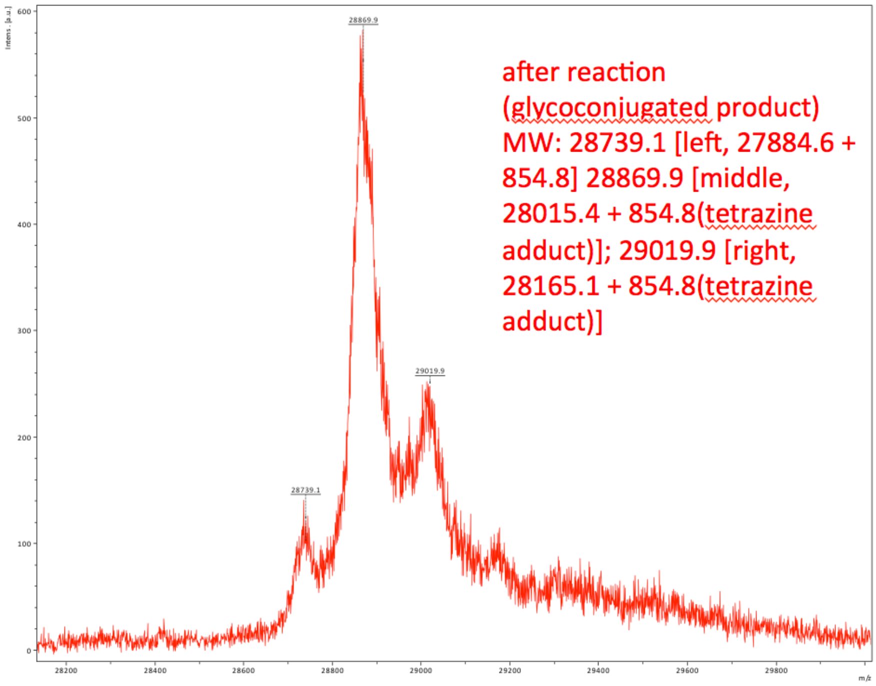

**Table 2**

Entry 1

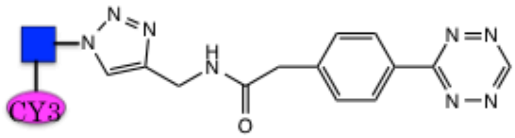


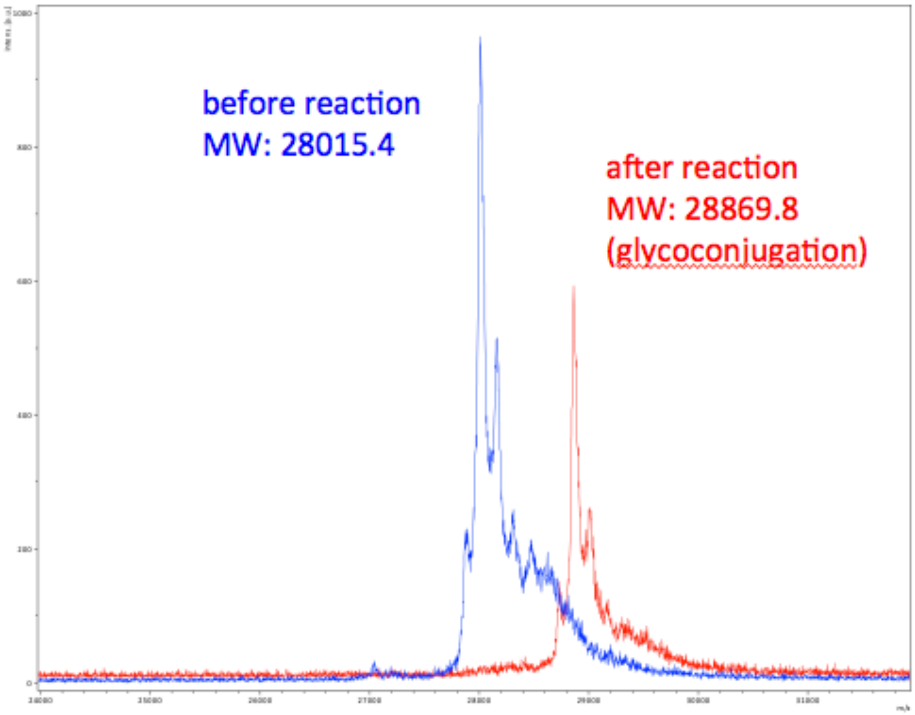


Entry 2

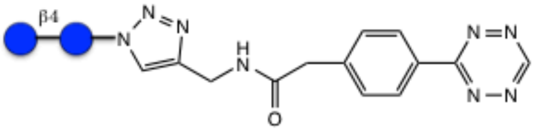


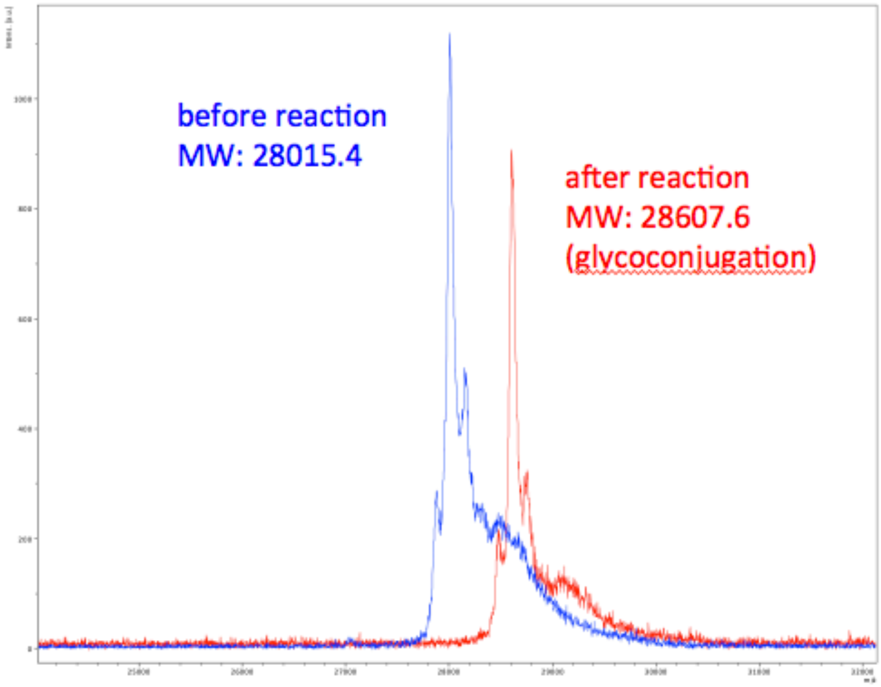

Entry 3

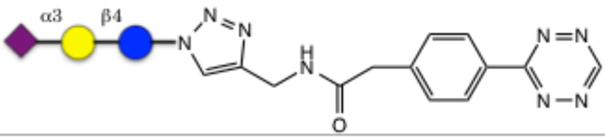

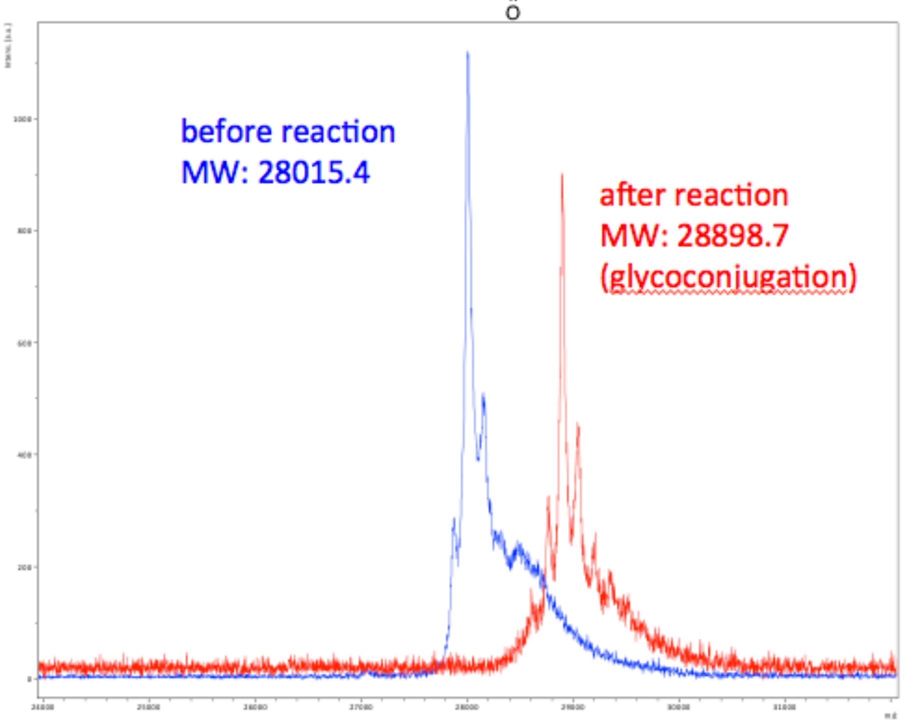


Entry 4

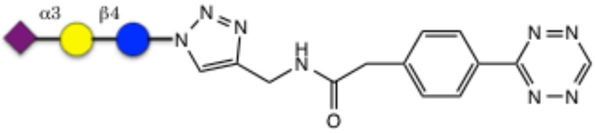

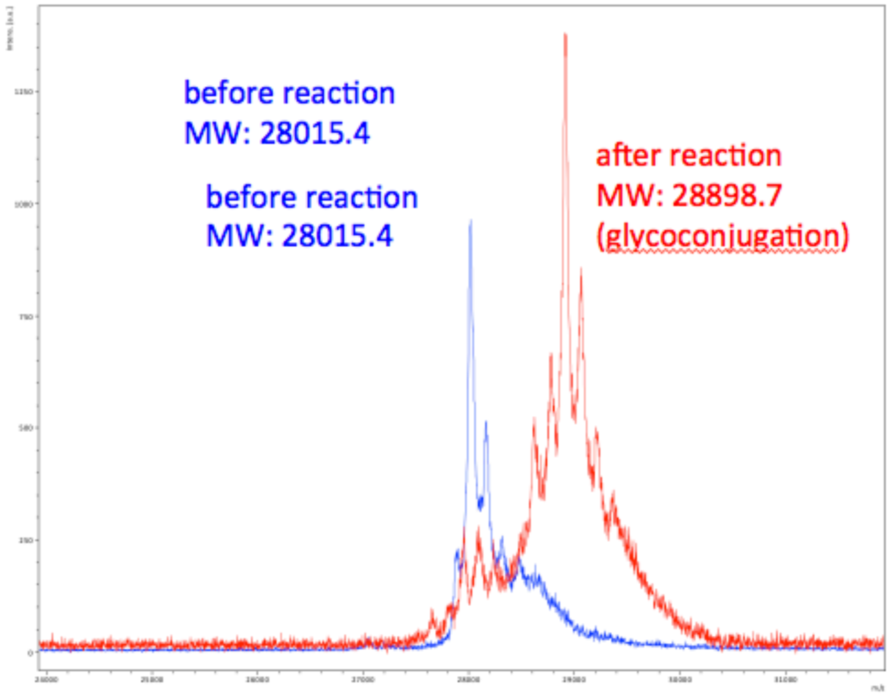

Entry 5 (10 min)

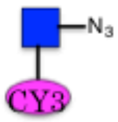


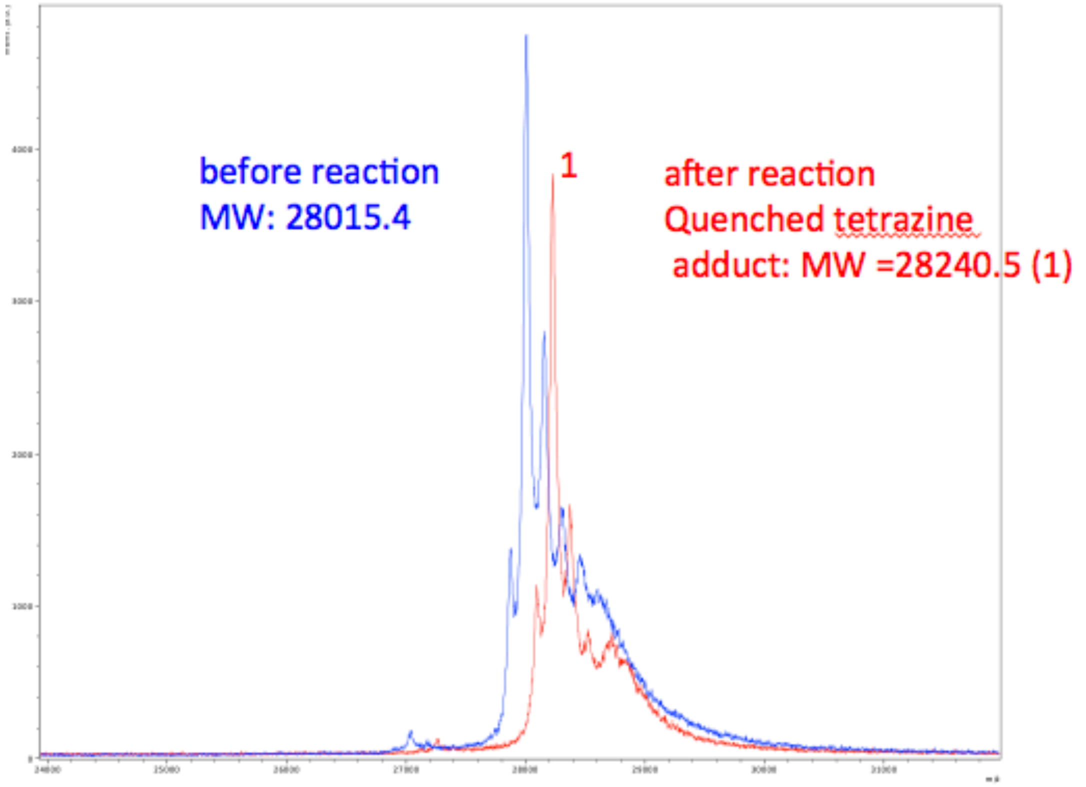


Entry 5 (3 h)

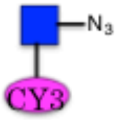


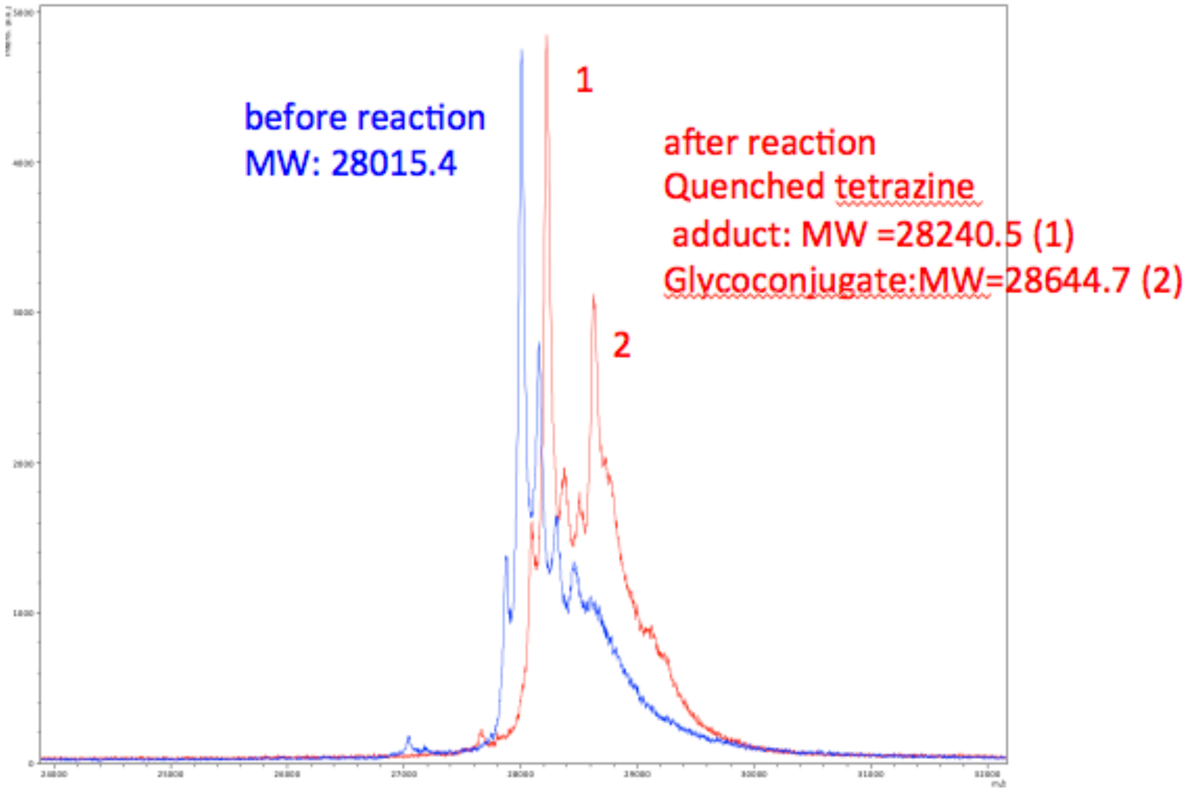

## 12. Spectra

**Tetrazine-glucose conjugate 3a**

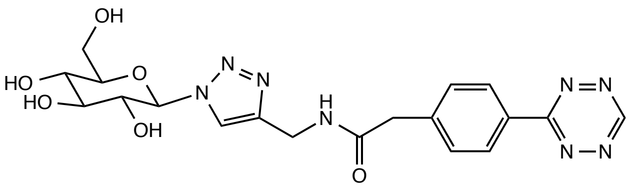

**H¹-NMR**

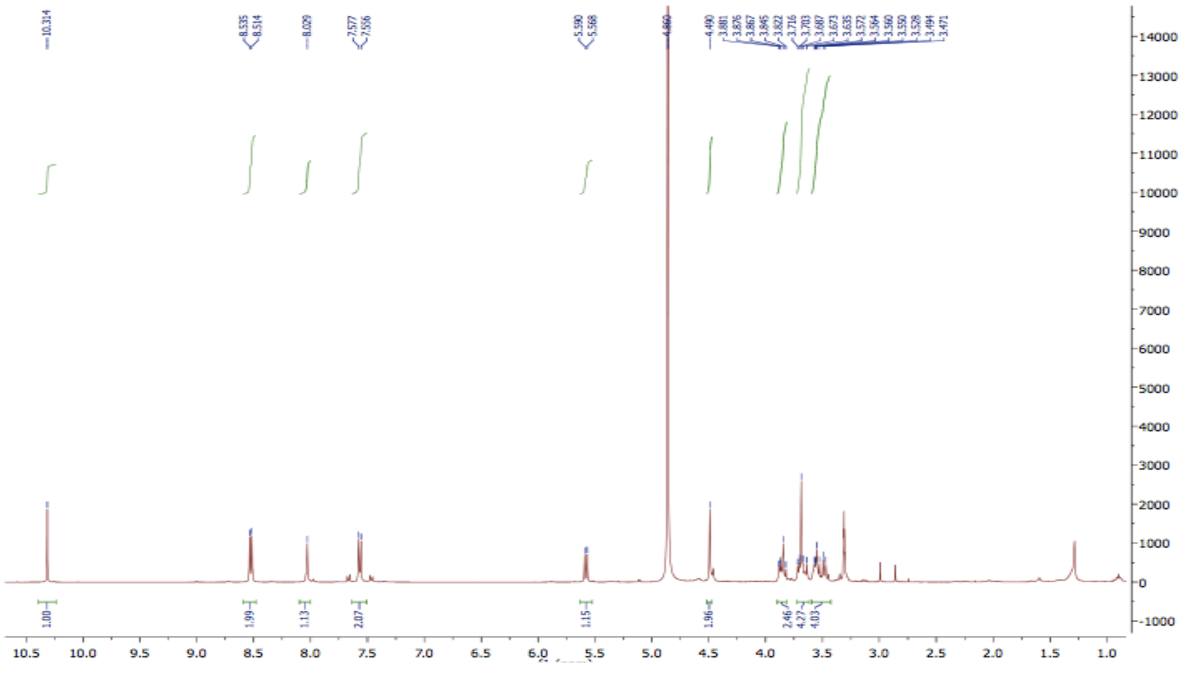

**C¹³-NMR**

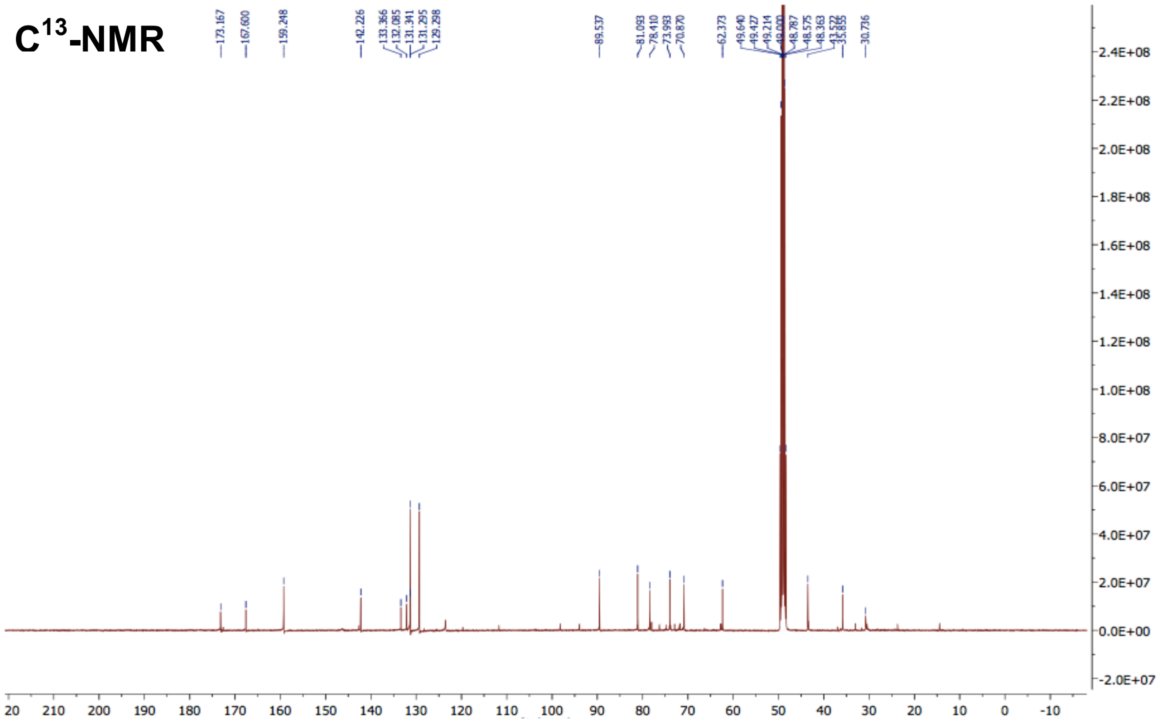

**Tetrazine-mannose conjugate 3b**

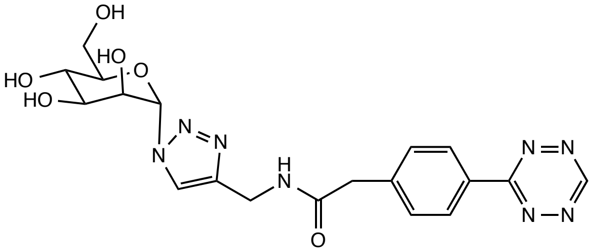


# H[1]-NMR

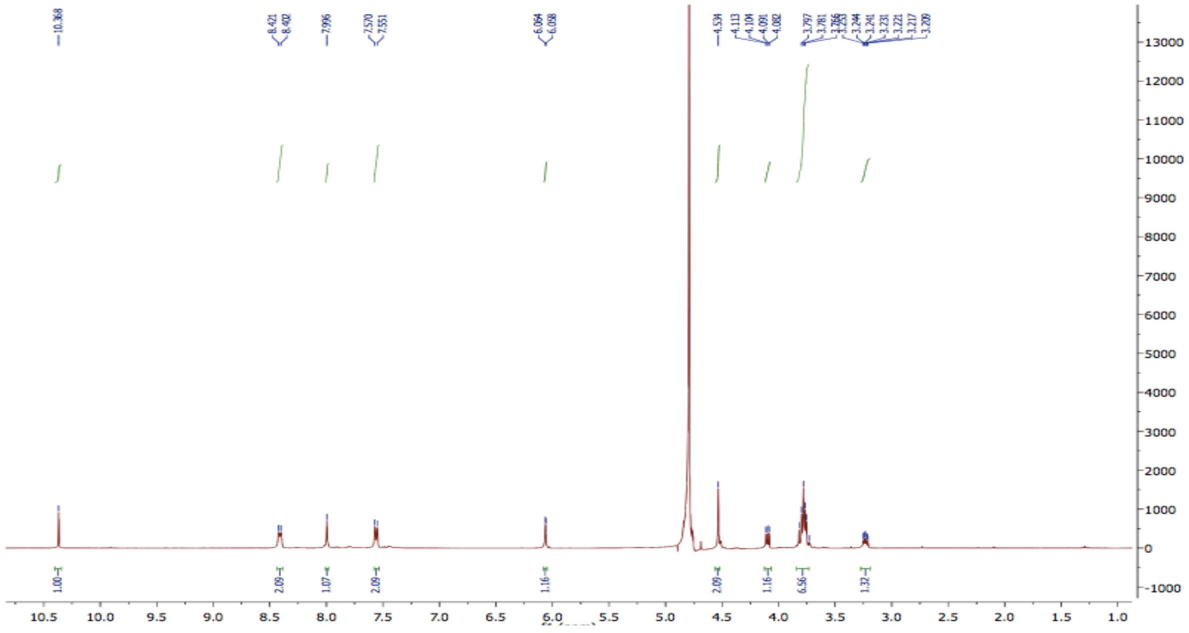


# C[13]-NMR

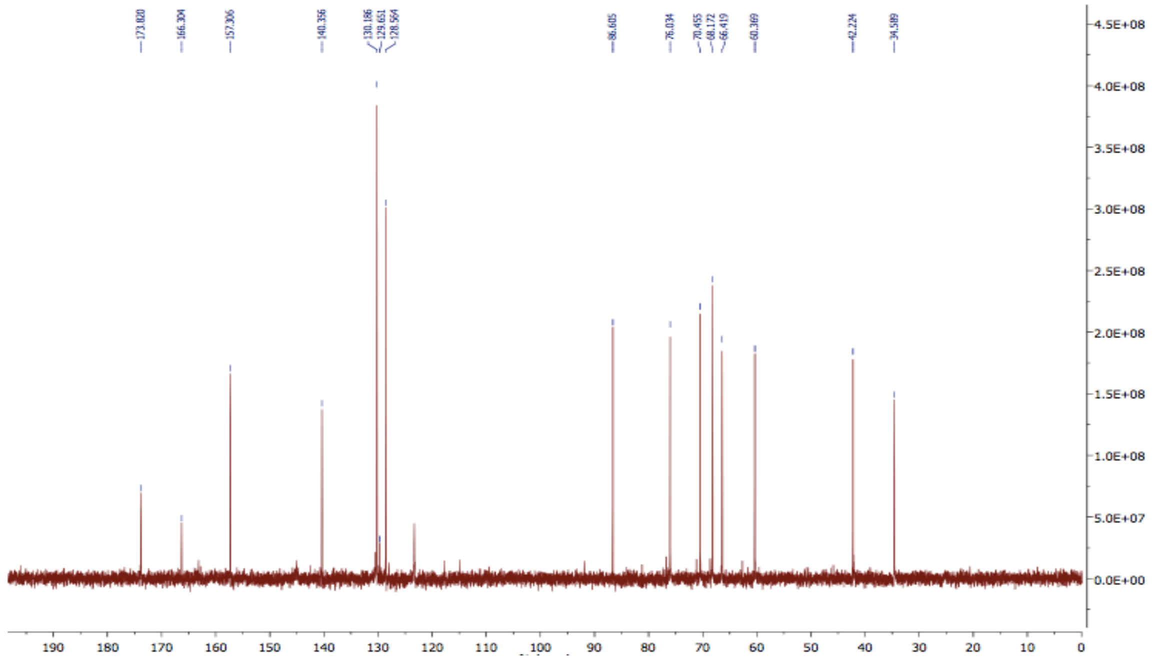

**Tetrazine-galactose conjugate 3c**

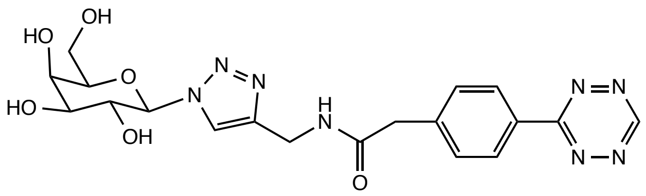

## H[1]-NMR

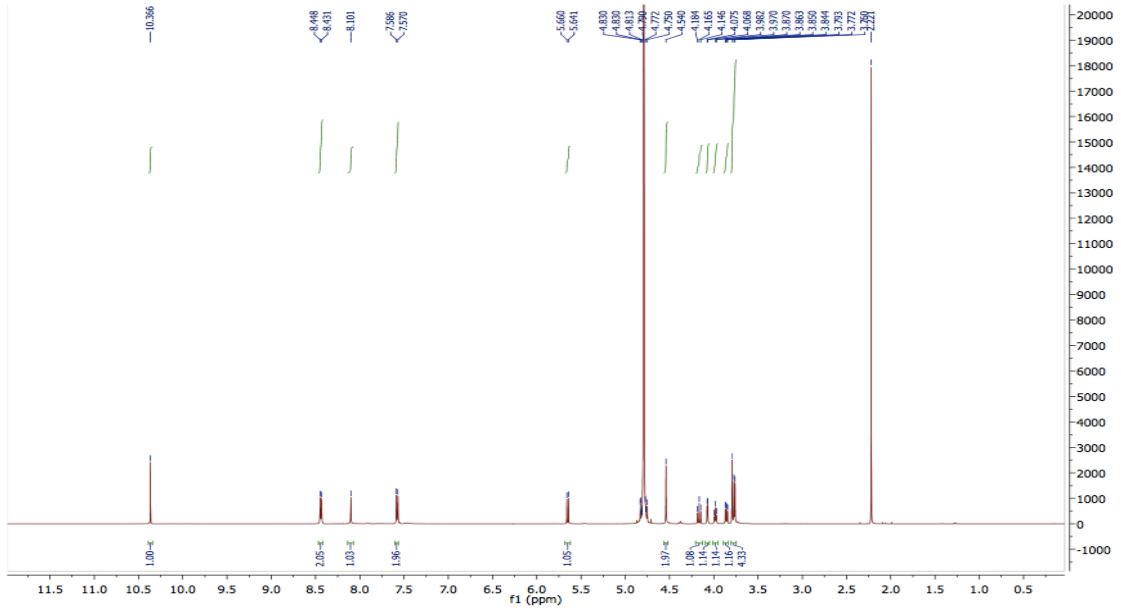

## C[13]-NMR

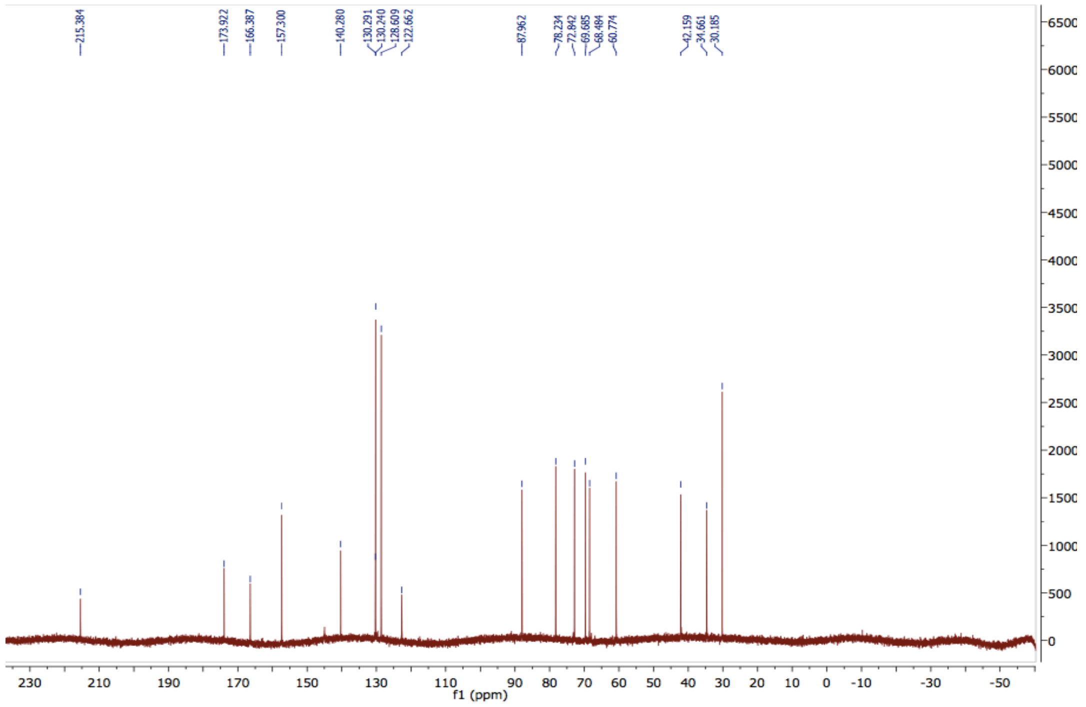

## H1-NMR

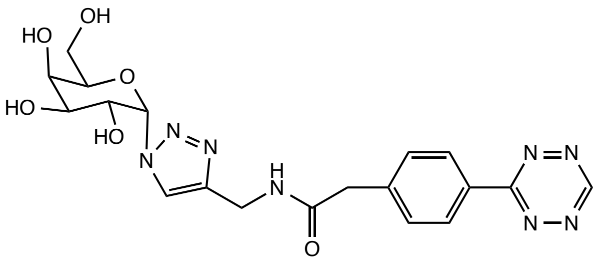


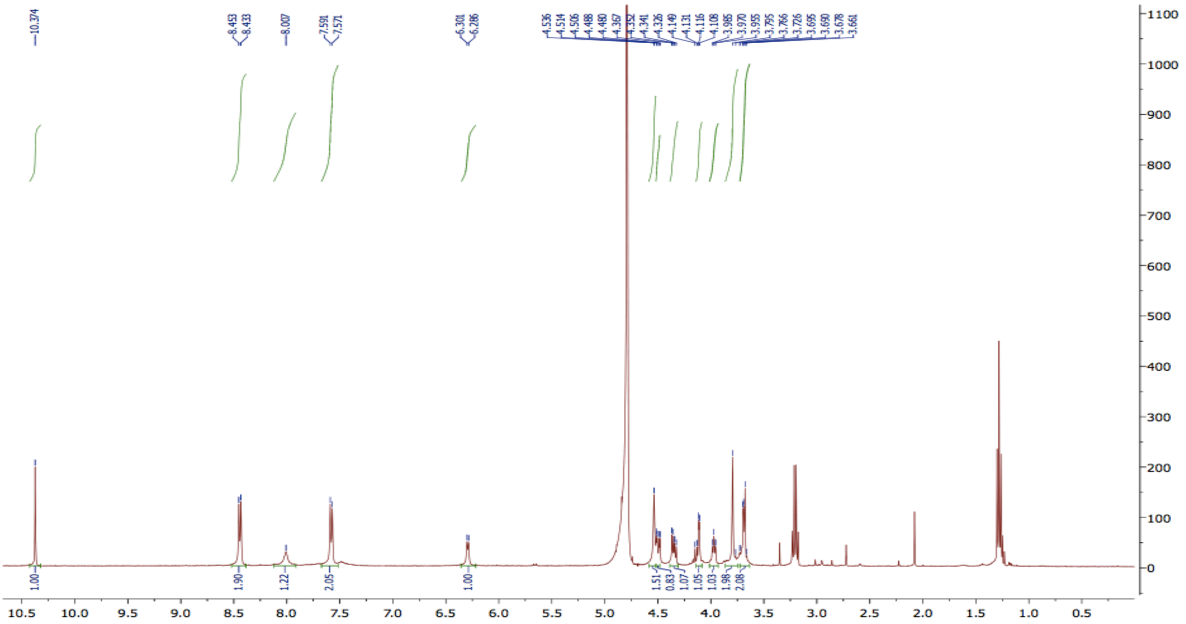


## C13-NMR

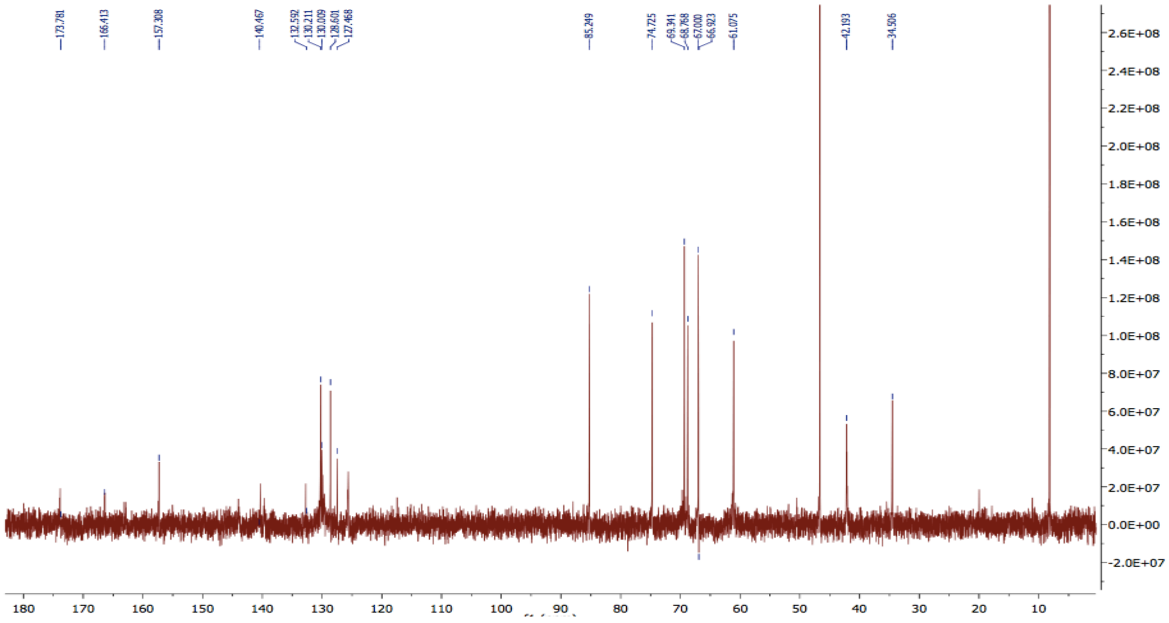

**Tetrazine-fucose conjugate 3d**

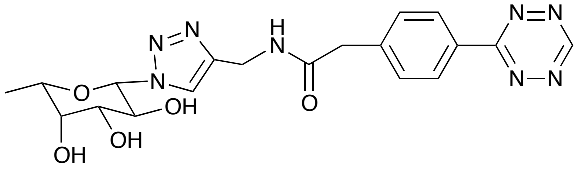

## H[1]-NMR

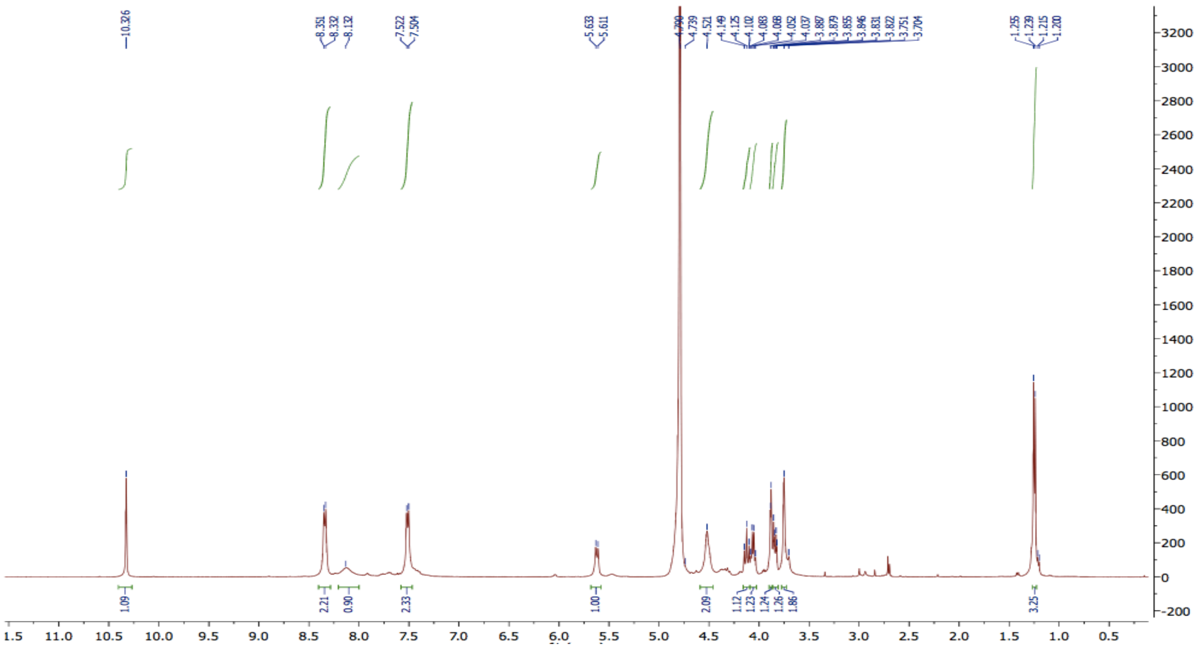

## C[13]-NMR

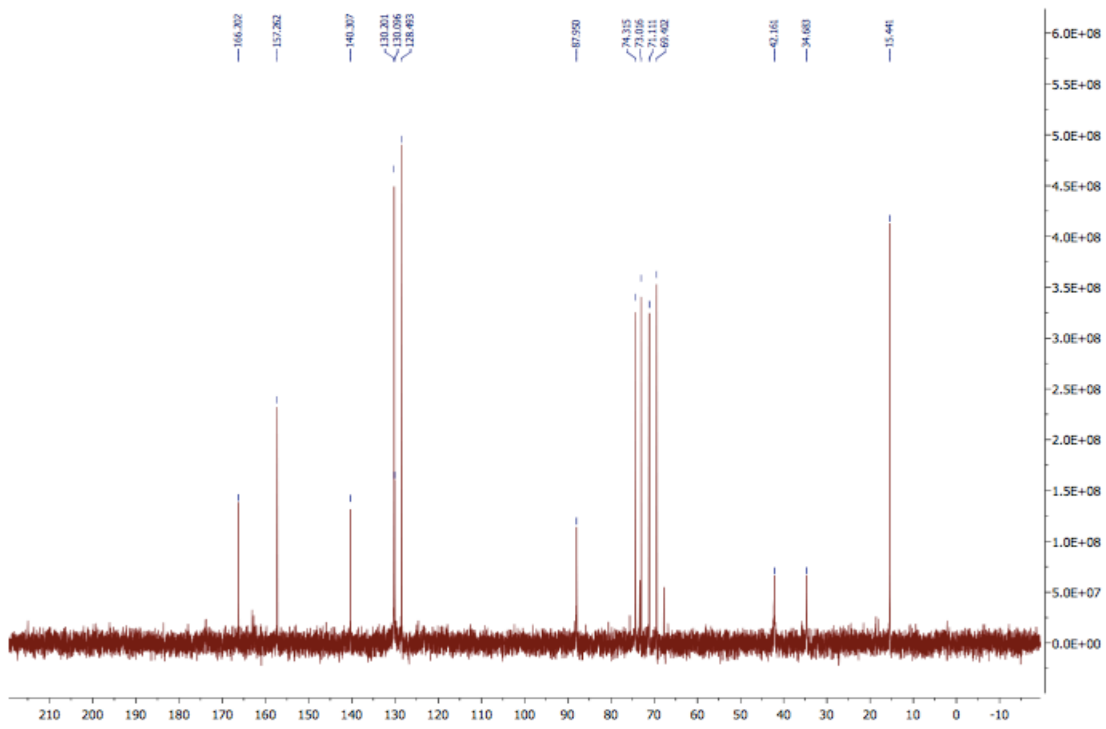

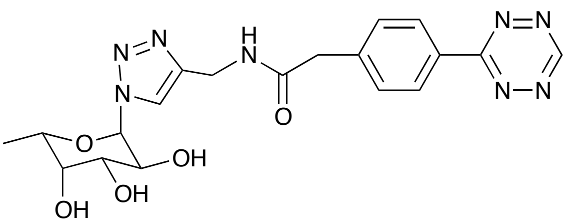

## H¹-NMR

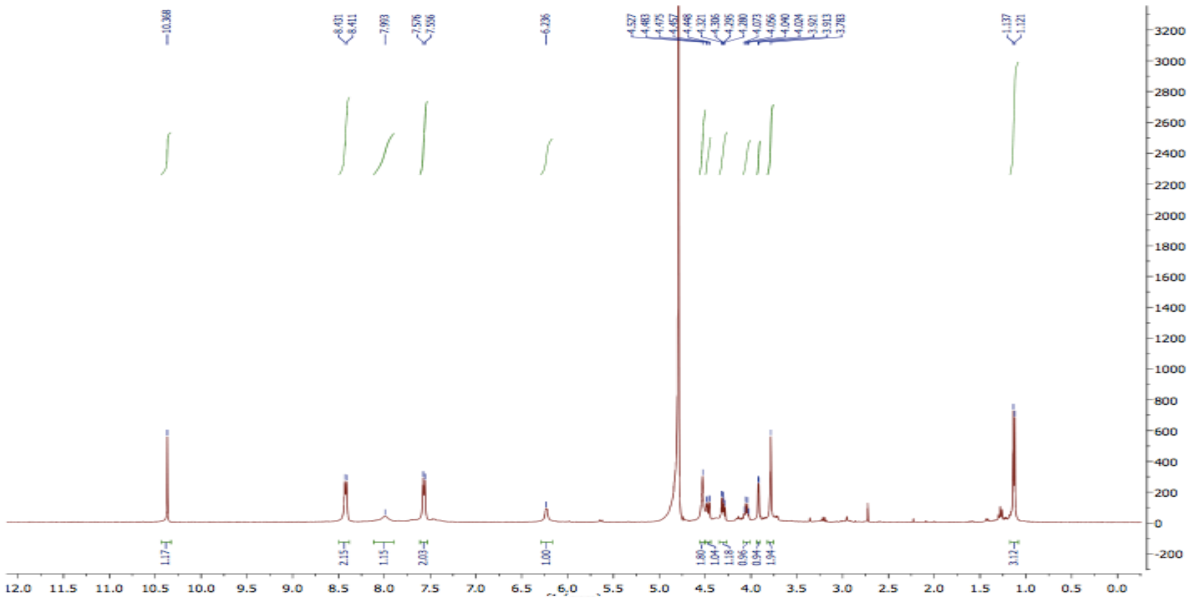

## C¹³-NMR

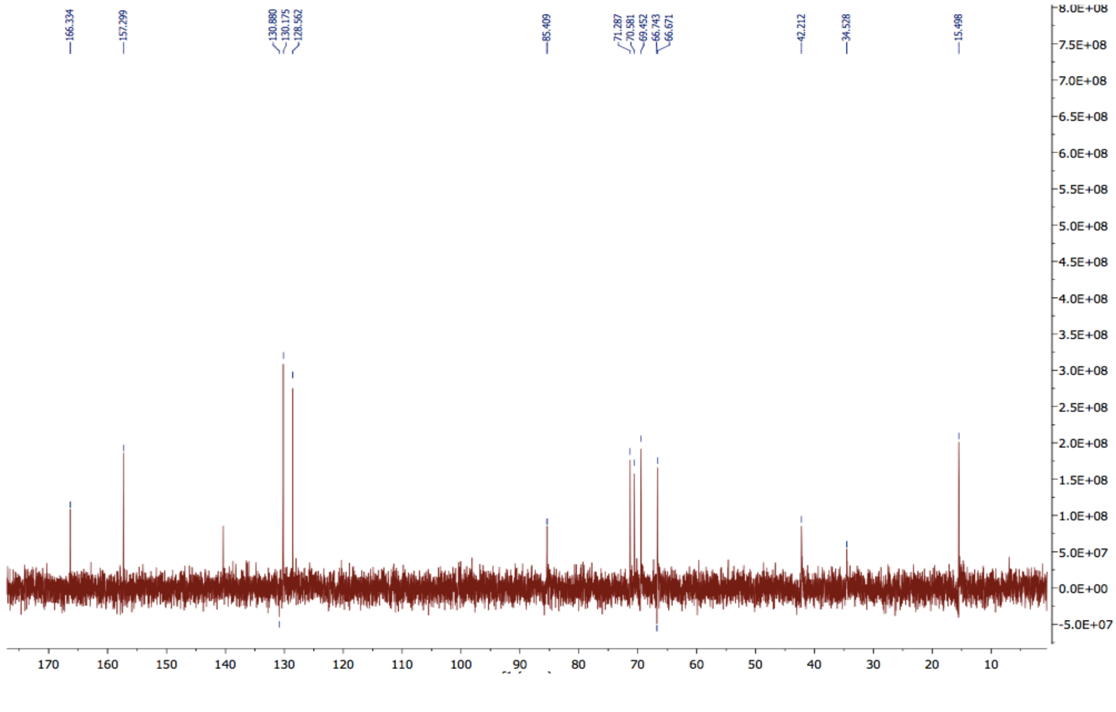

**Tetrazine-*N*-actyl glucosamine conjugate 3e**

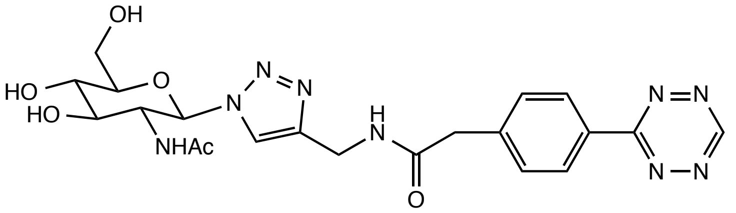


## H1-NMR

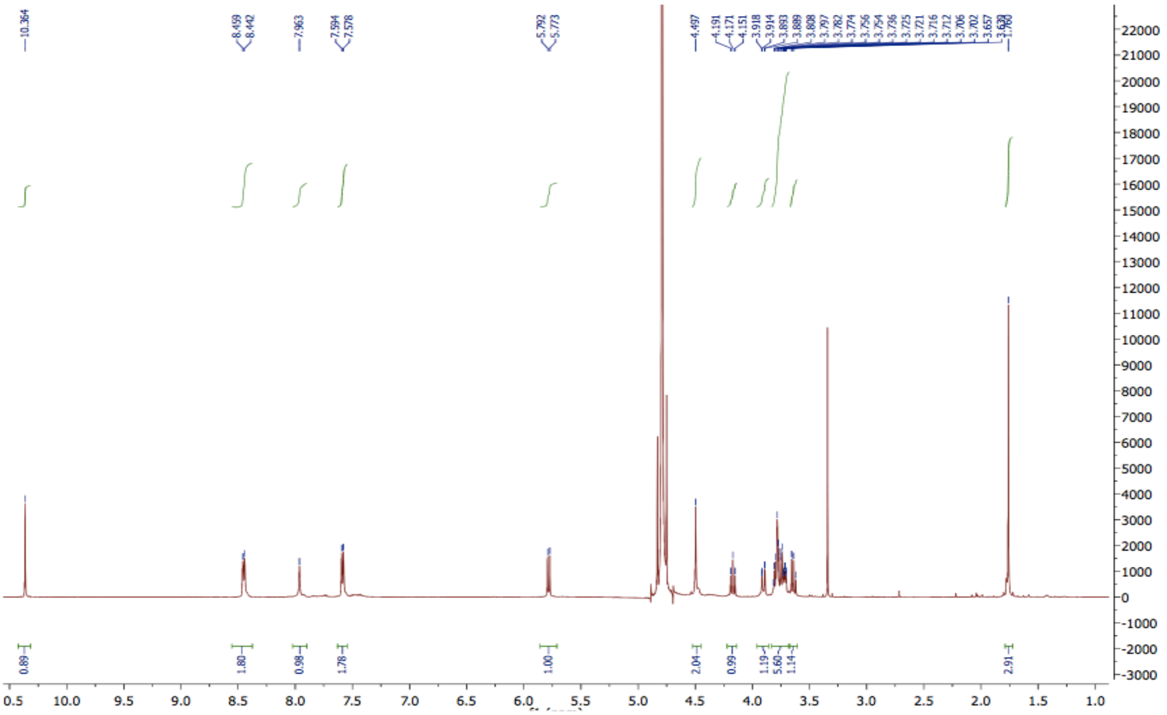


## C13-NMR

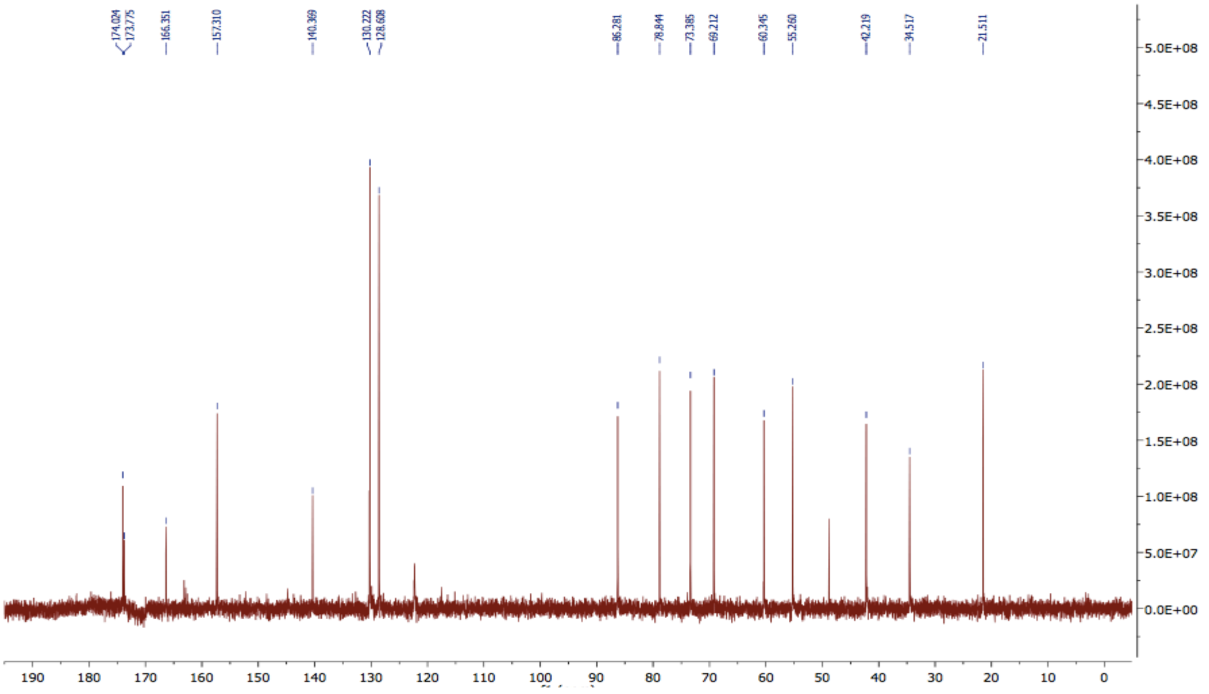

**Tetrazine-glucoronic acid conjugate 3f**

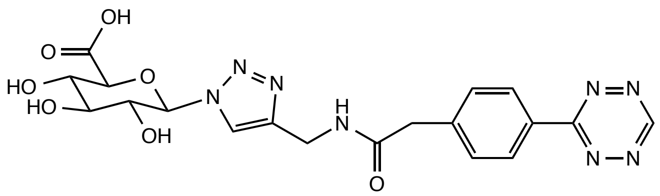

# H1-NMR

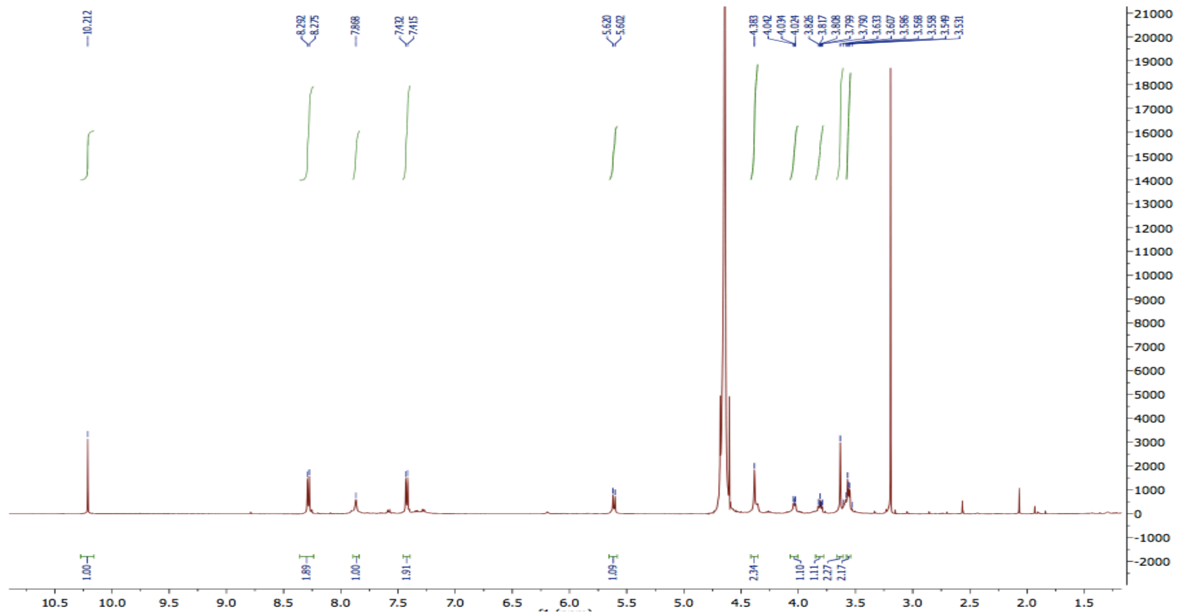

# C13-NMR

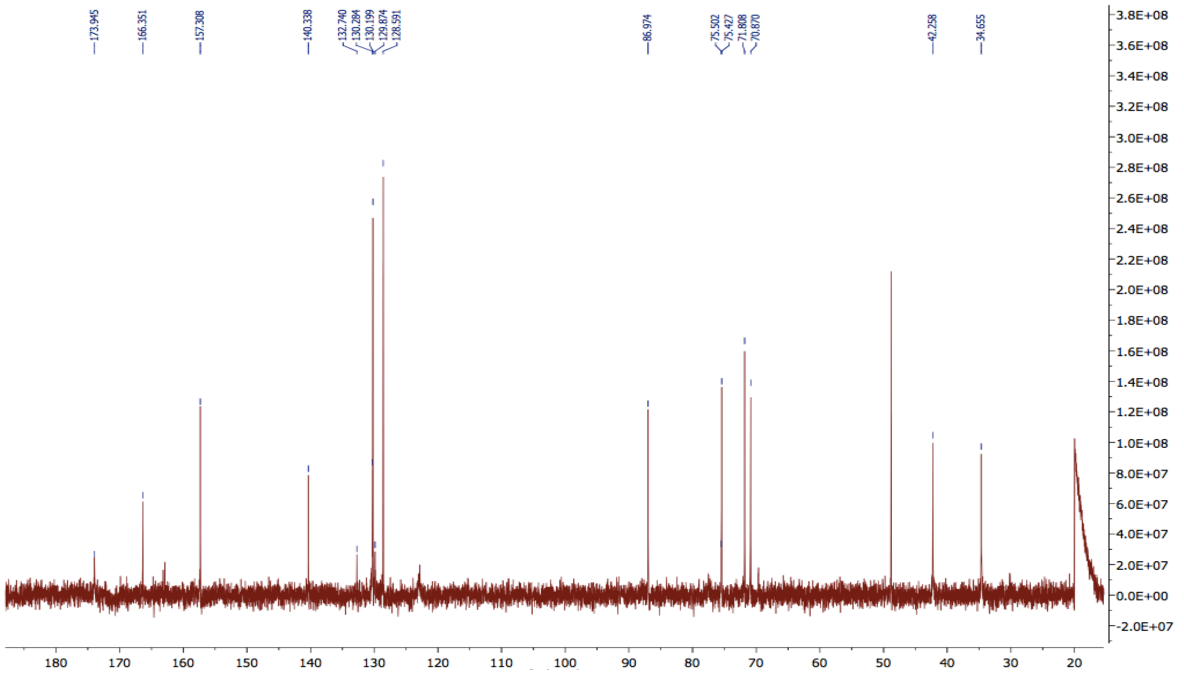

**Tetrazine-maltose conjugate 3g**

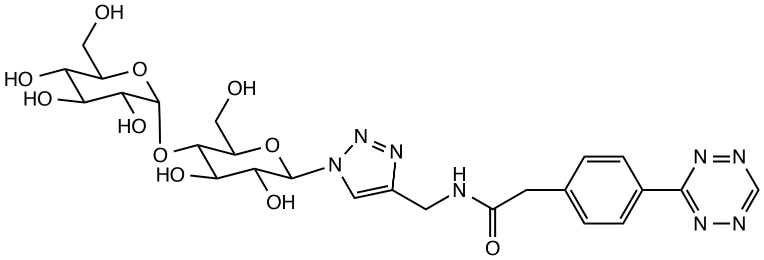

## $H^1$-NMR

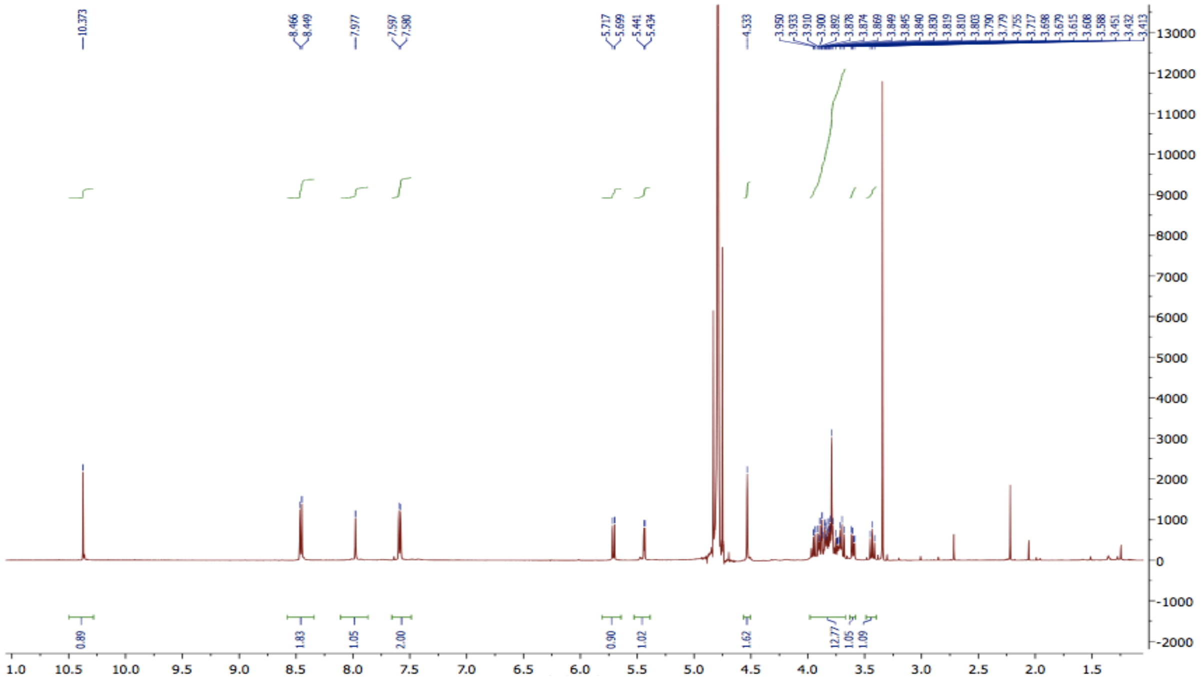

## $C^{13}$-NMR

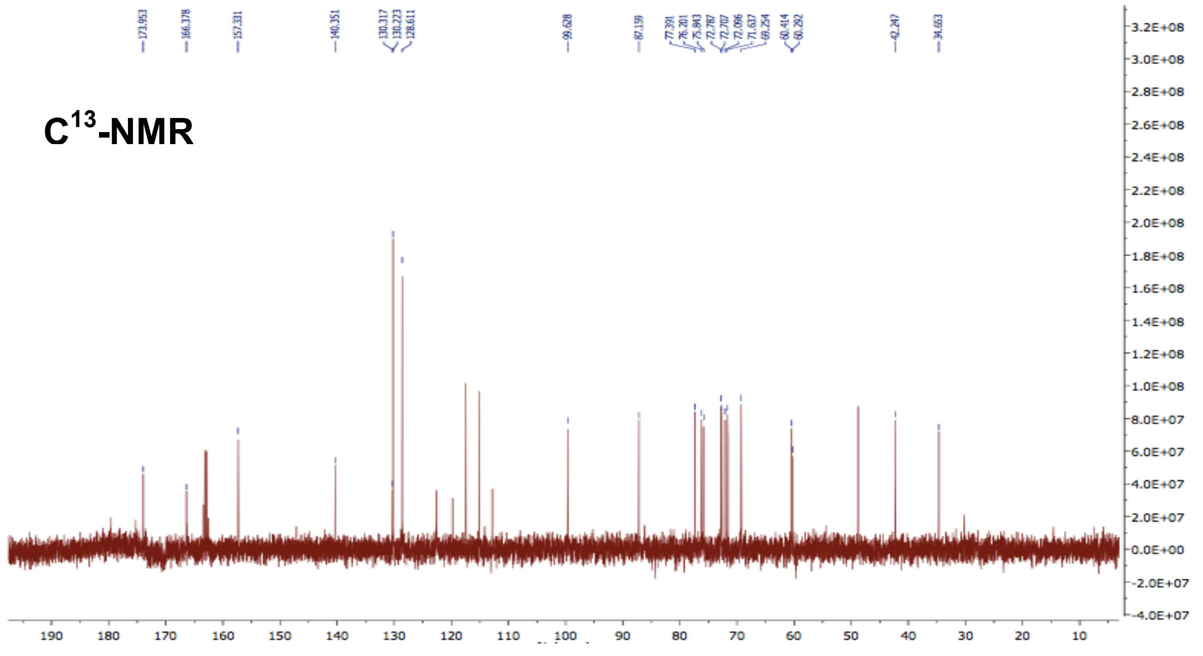

**Tetrazine-cellobiose conjugate 3h**

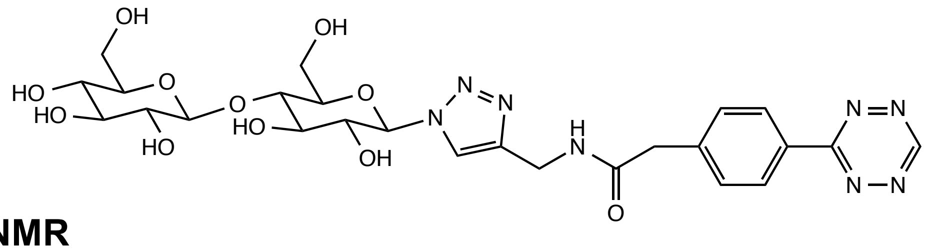


## H[1]-NMR

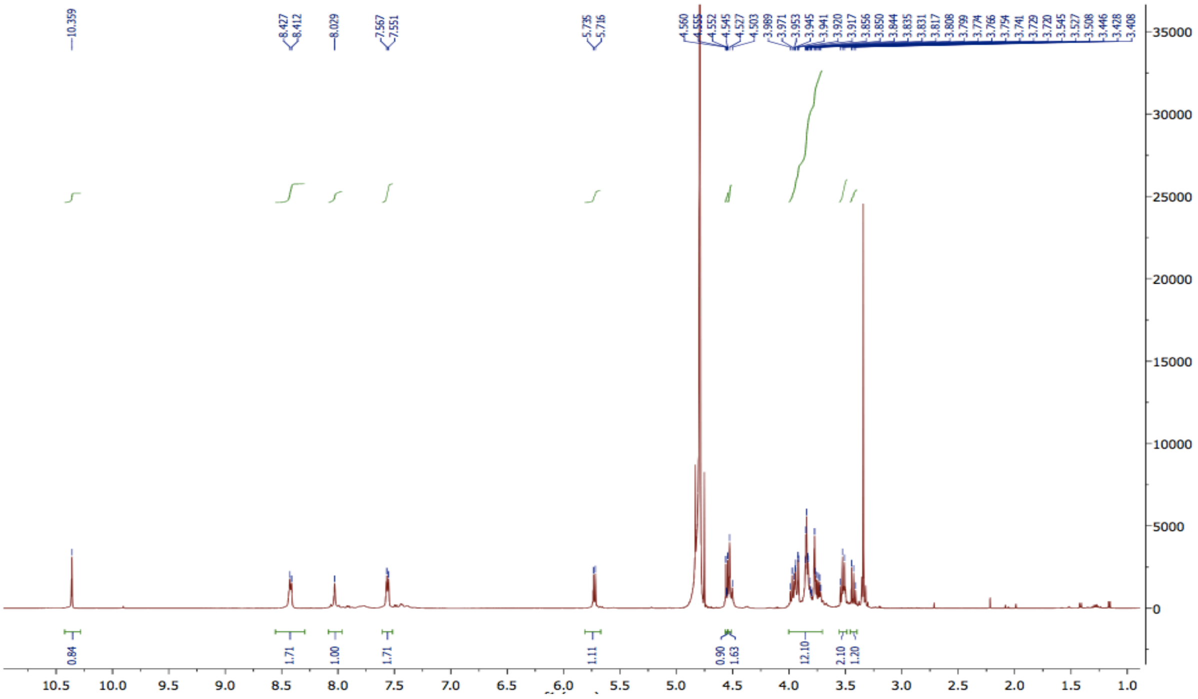


## C[13]-NMR

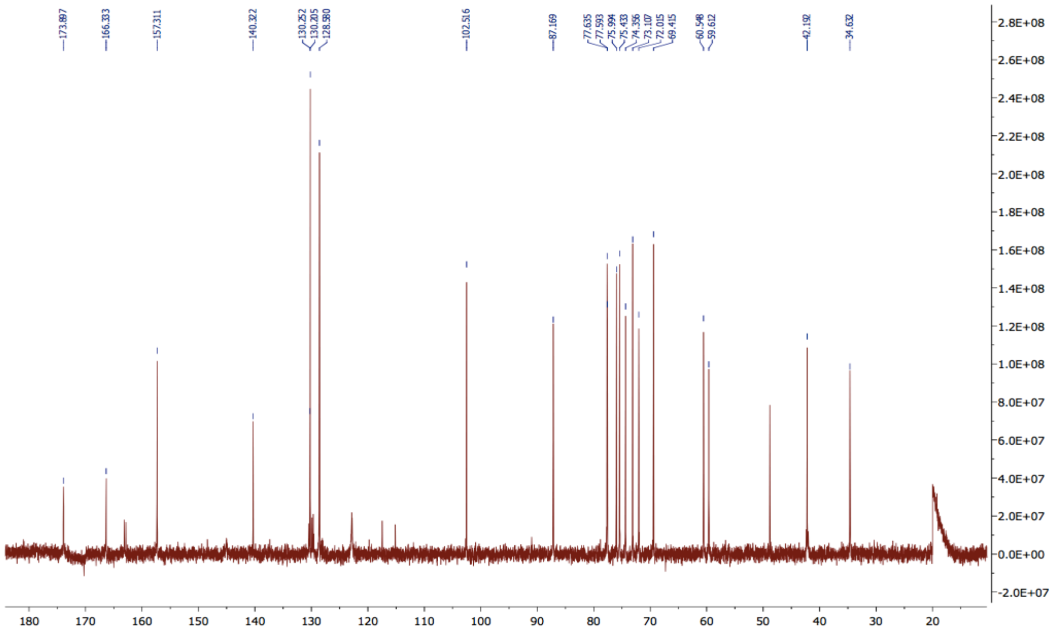

**Tetrazine-lactose conjugate 3i**

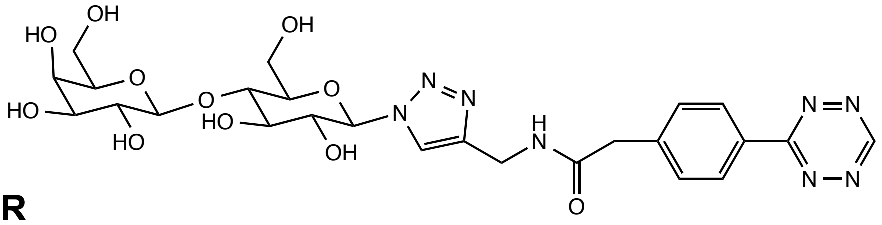

## H[1]-NMR

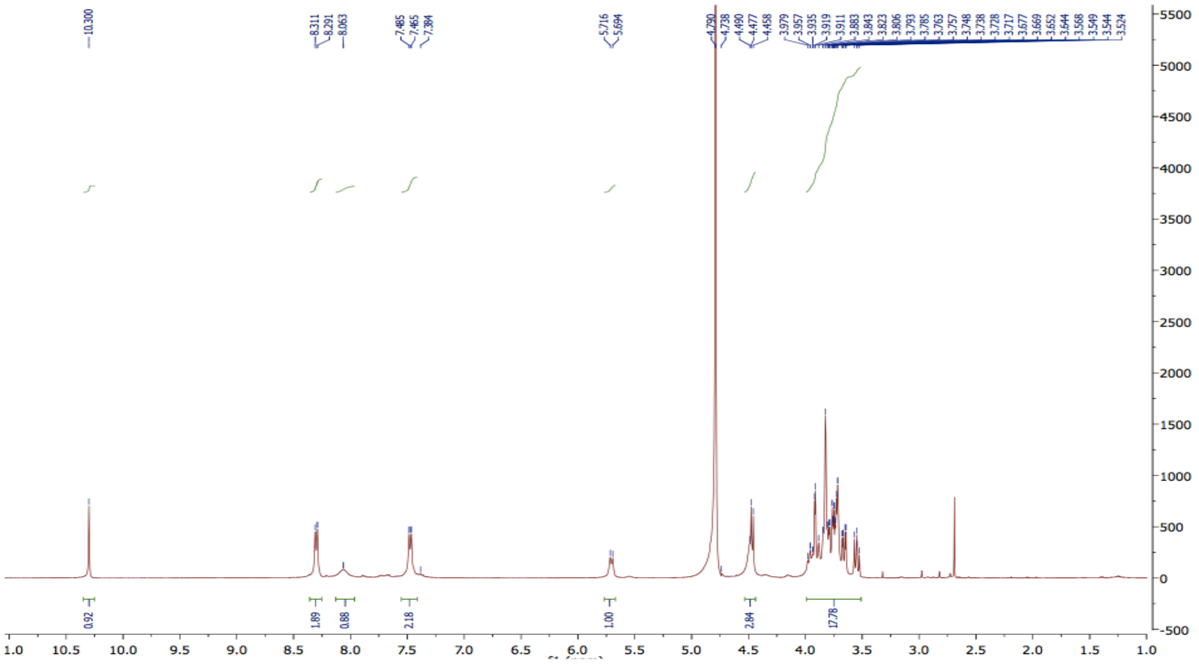

## C[13]-NMR

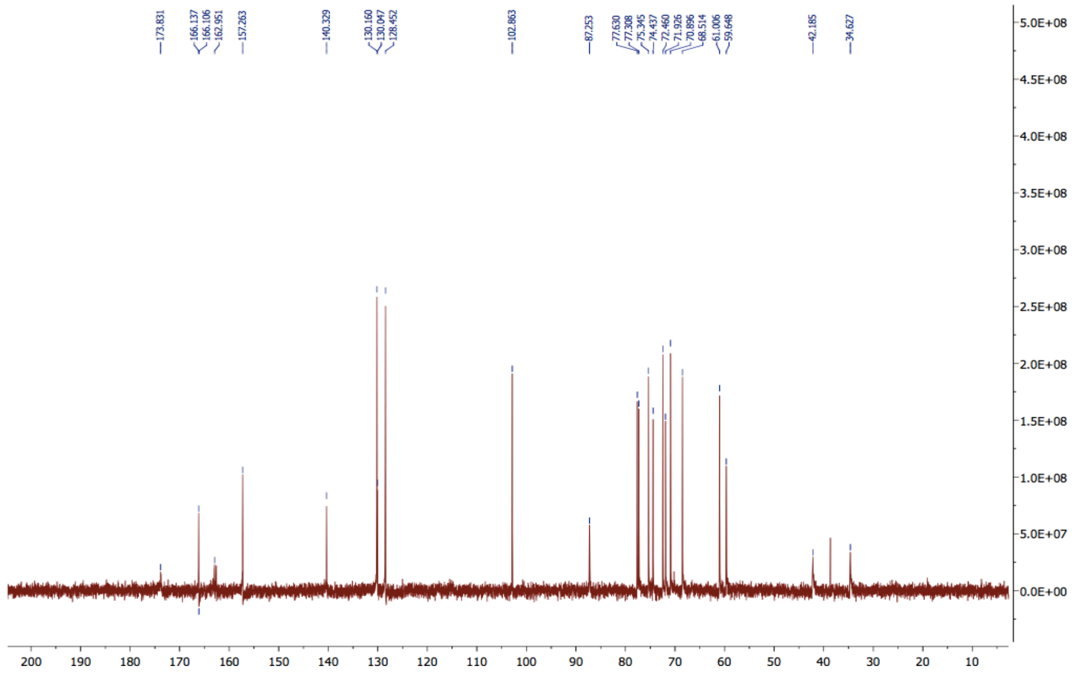

**Tetrazine Fucα1-2Galβ1-3[Fucα1-4]GlcNAcβ1-3Galβ1-4Glc conjugate 3j**

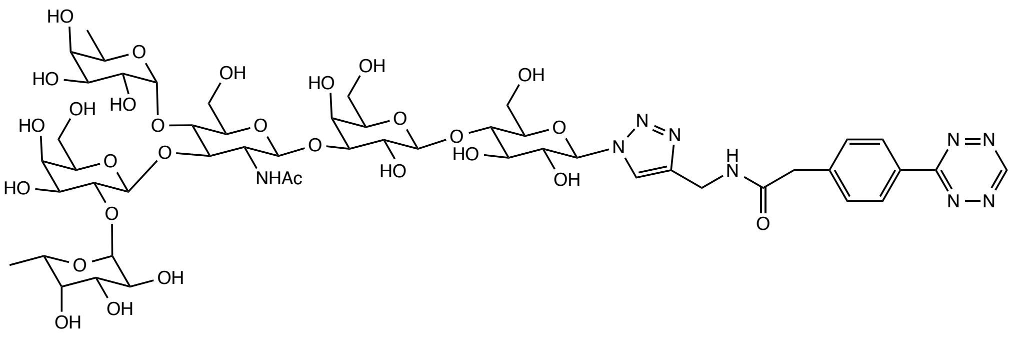

**LC-MS**

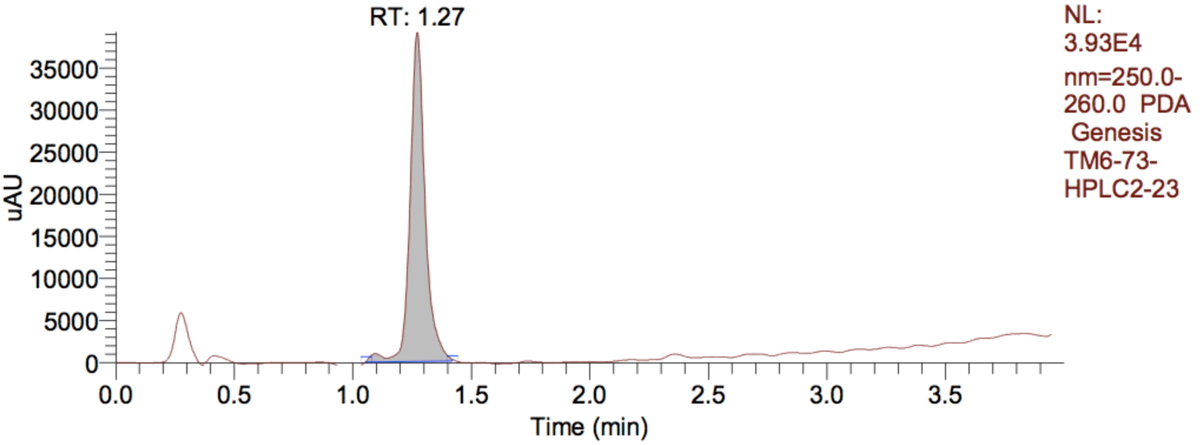


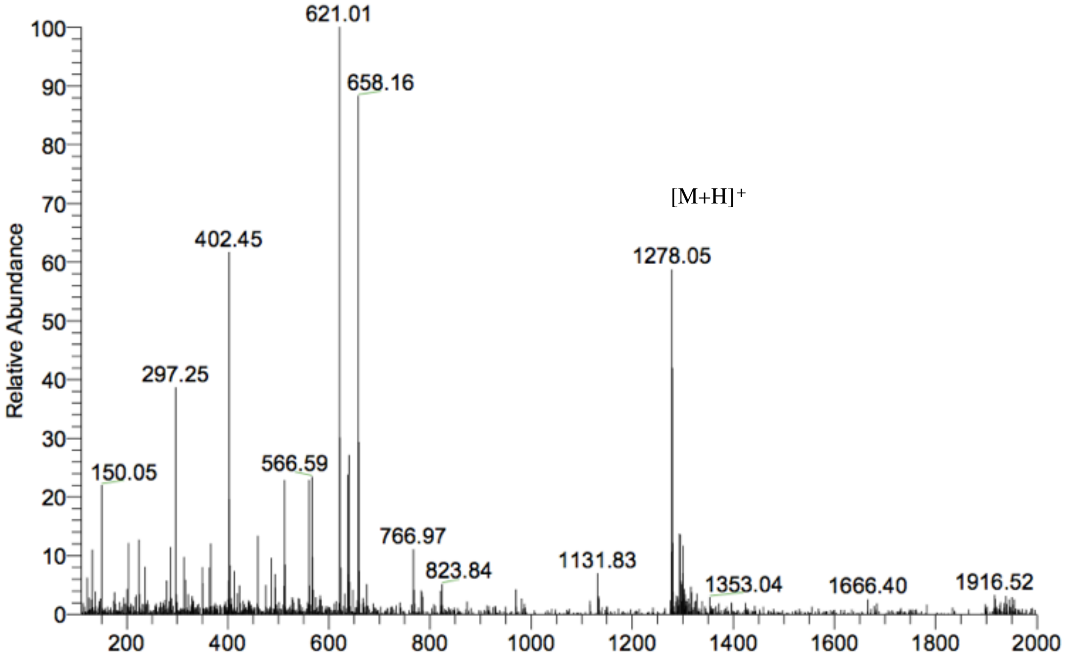

## H1-NMR

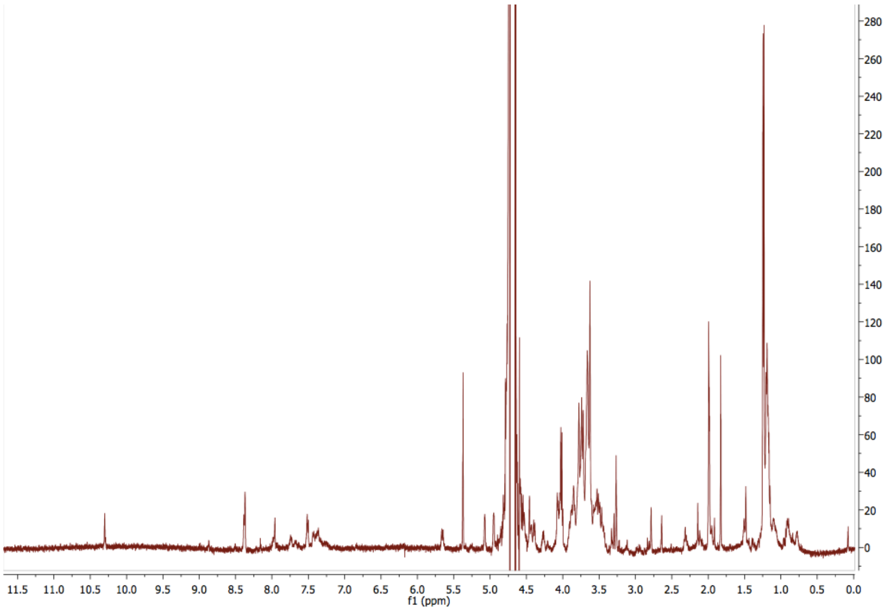


## MALDI-TOF mass

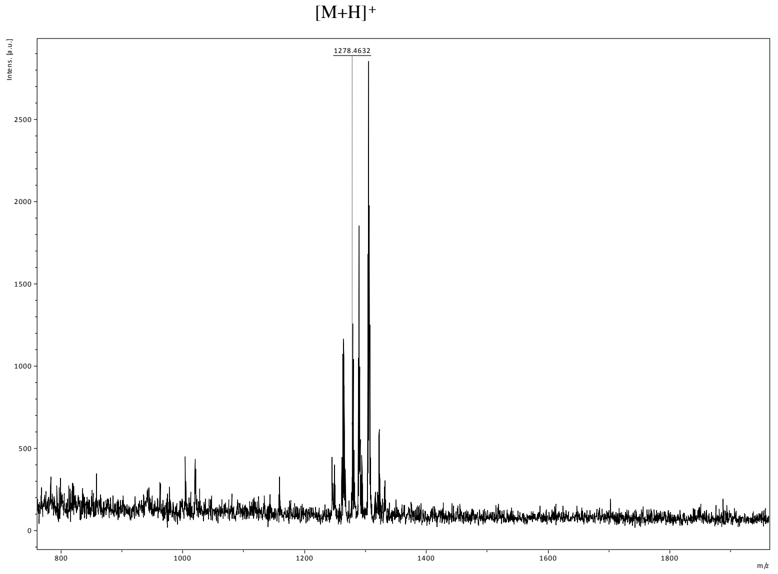


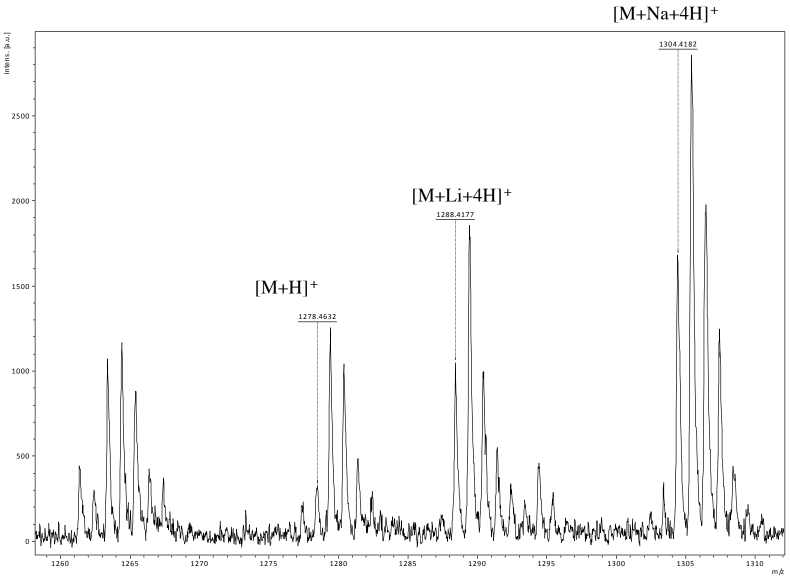

**Tetrazine-β-3'-sialyllactose conjugate 3k**

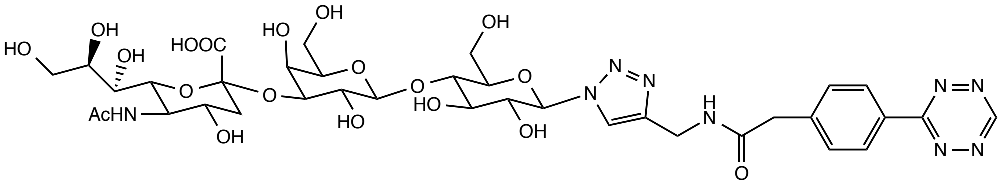


## LC-MS

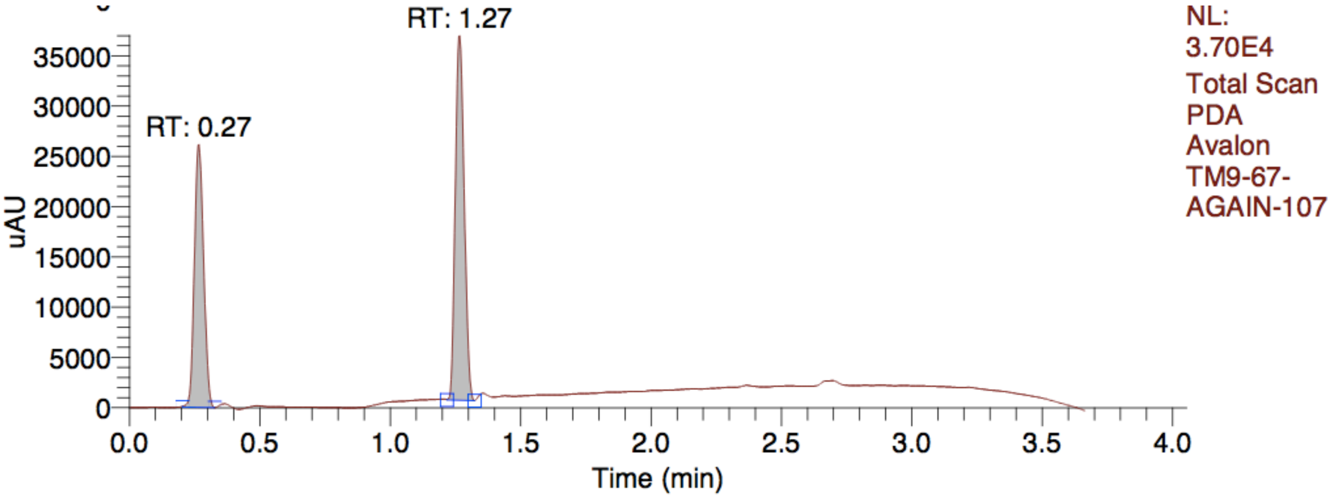


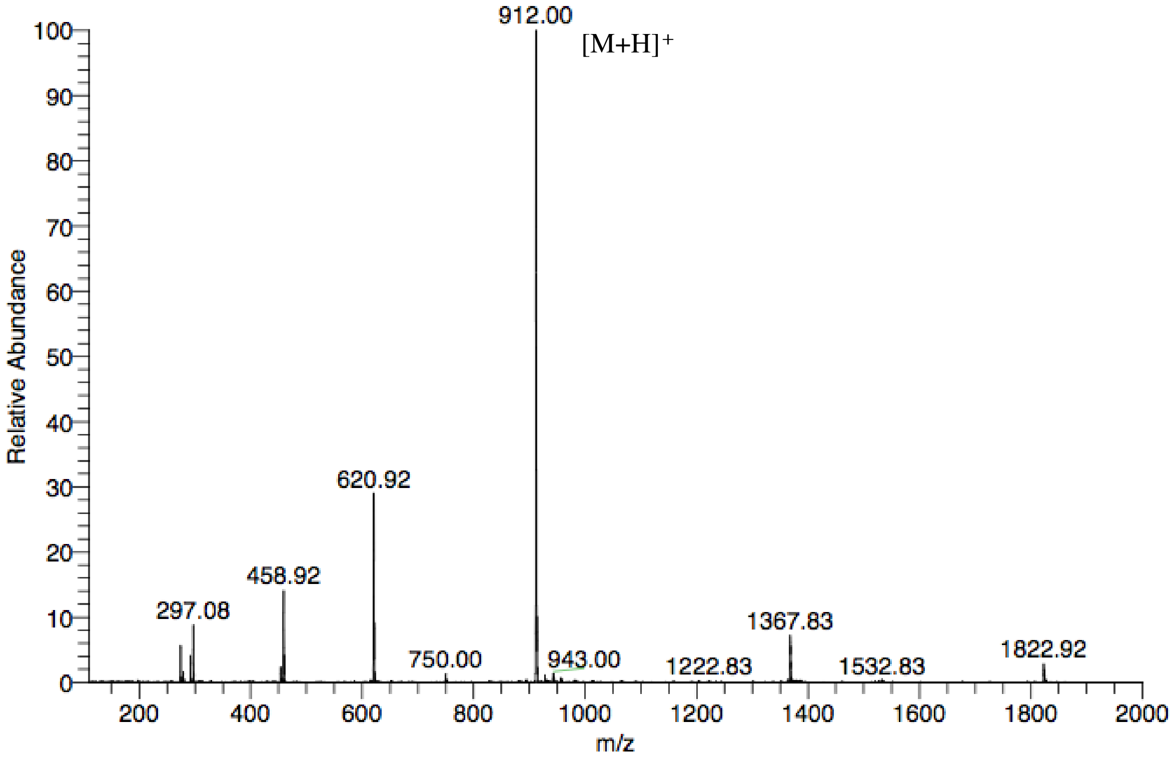

## H1-NMR

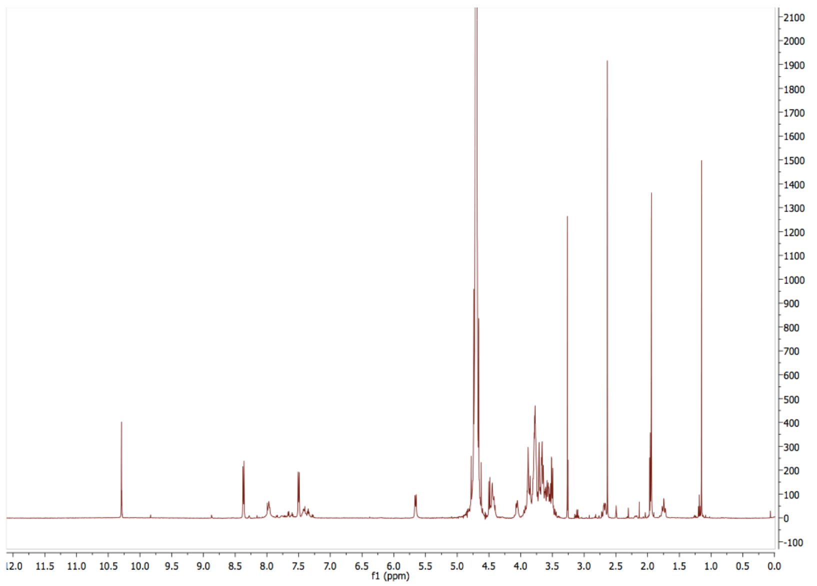

## MALDI-TOF

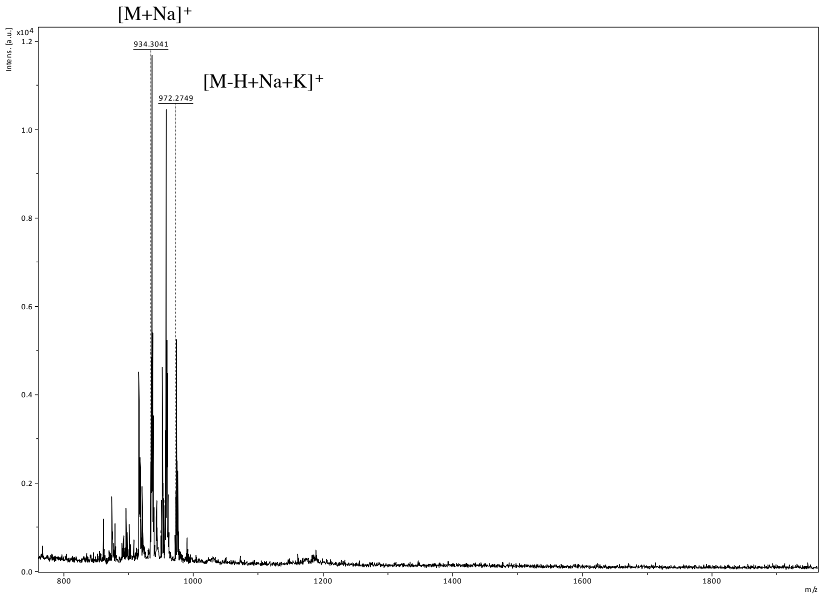


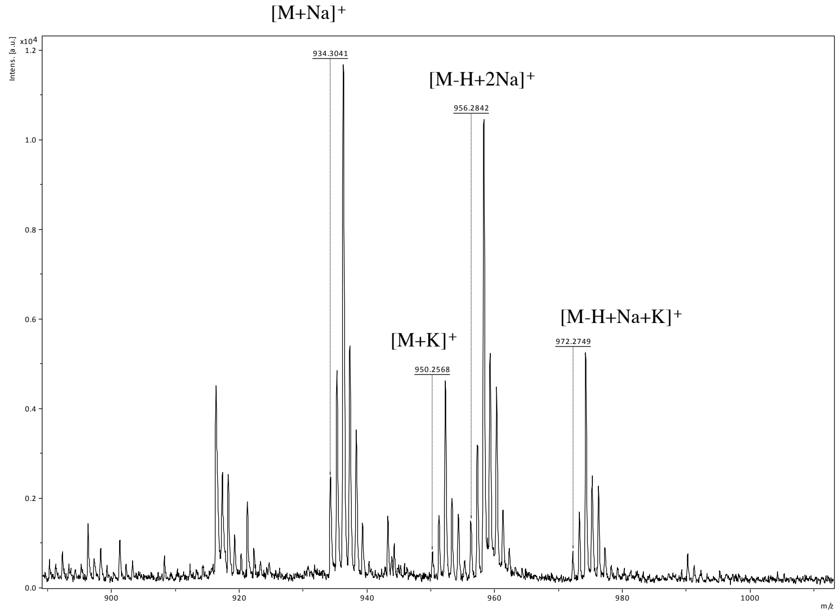

**Tetrazine-β-6'-sialyllactose conjugate 3l**

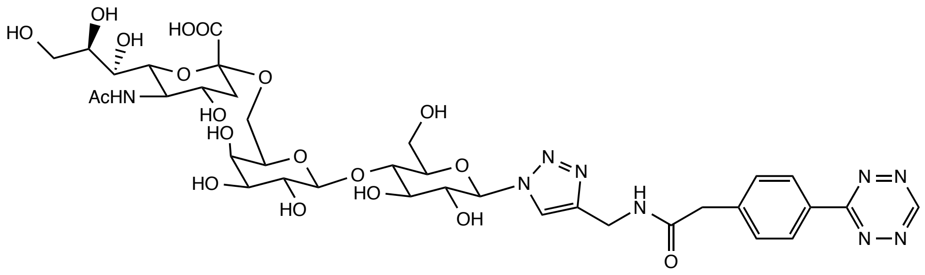


## LC-MS

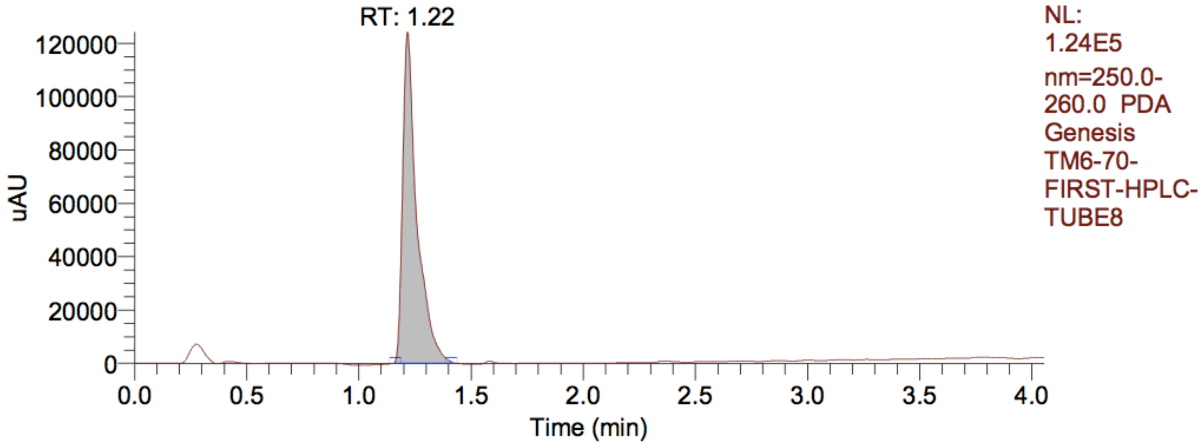


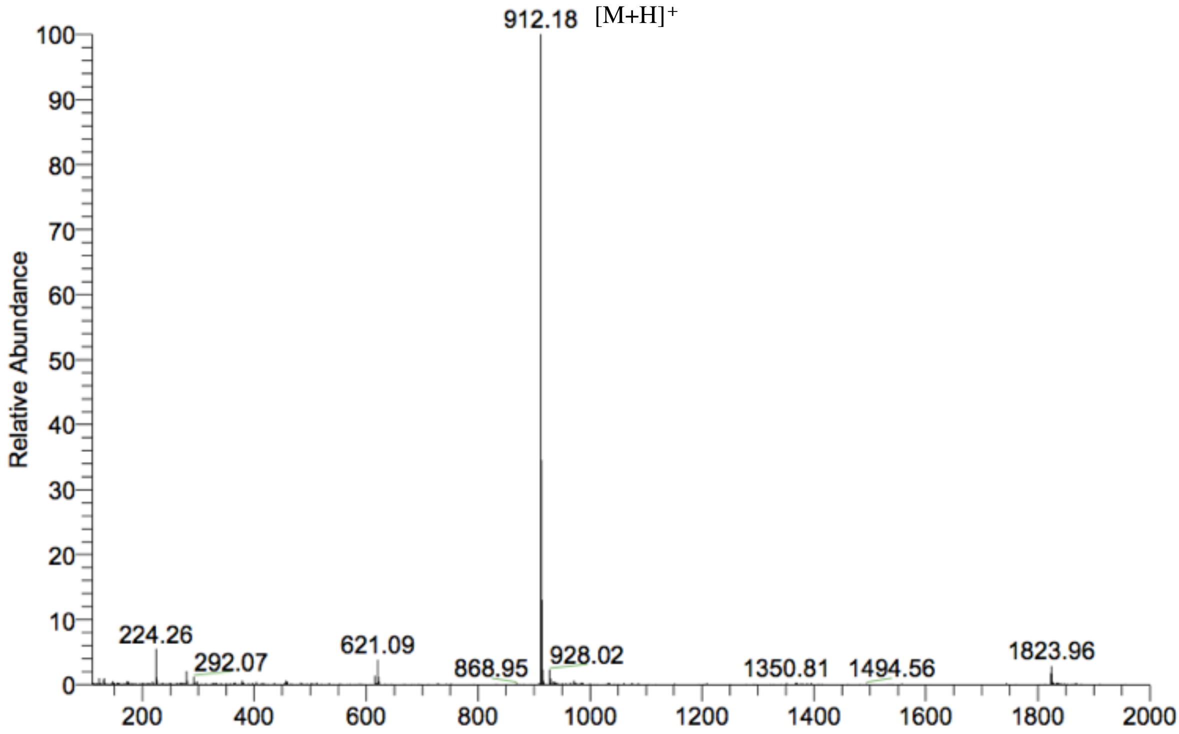

# $H^1$-NMR

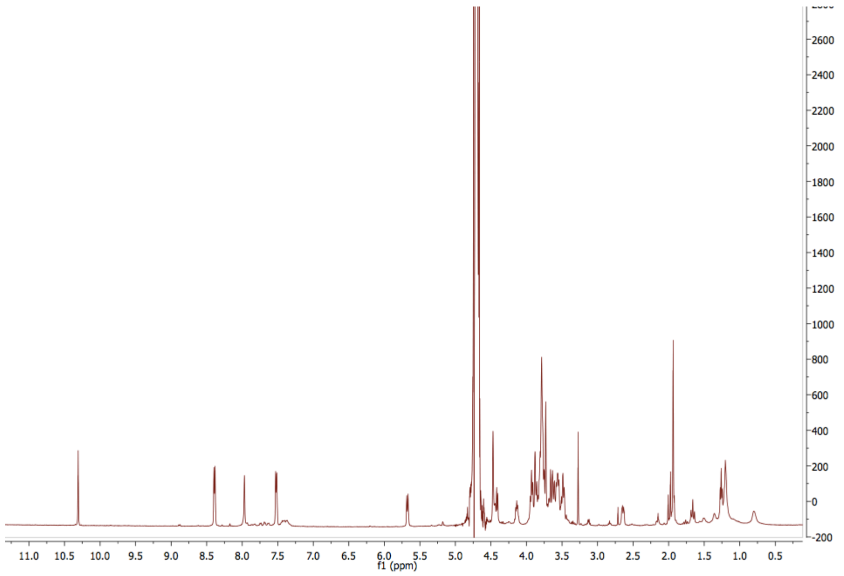


# MALDI-TOF

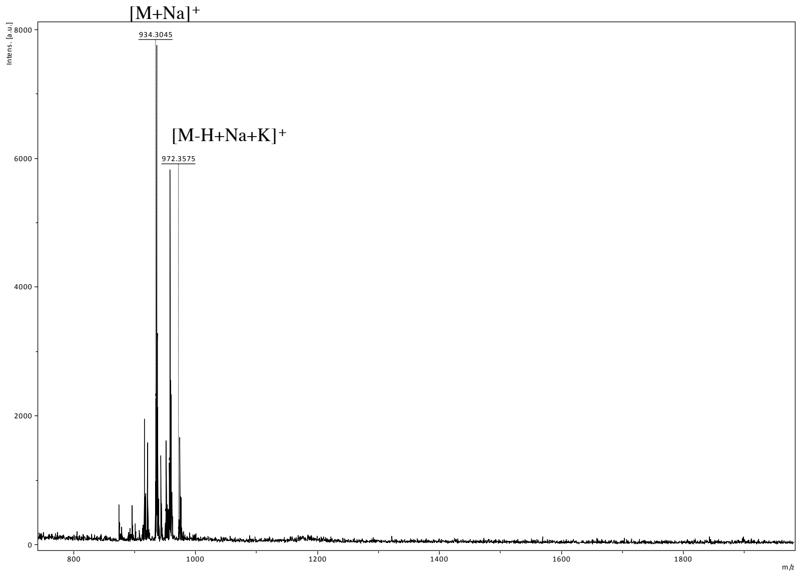


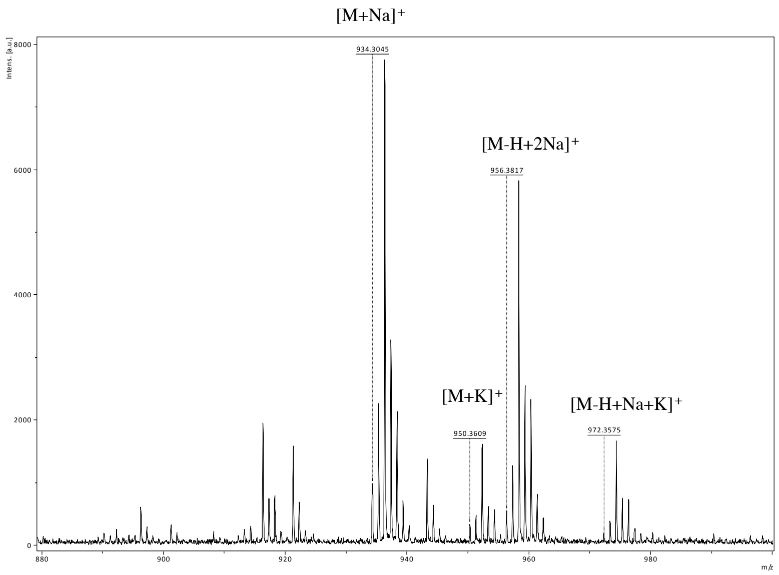

### *N*-CY3-Glucosamine (S4)

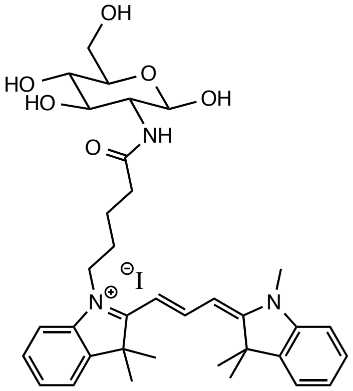


**S4**

## LC-MS

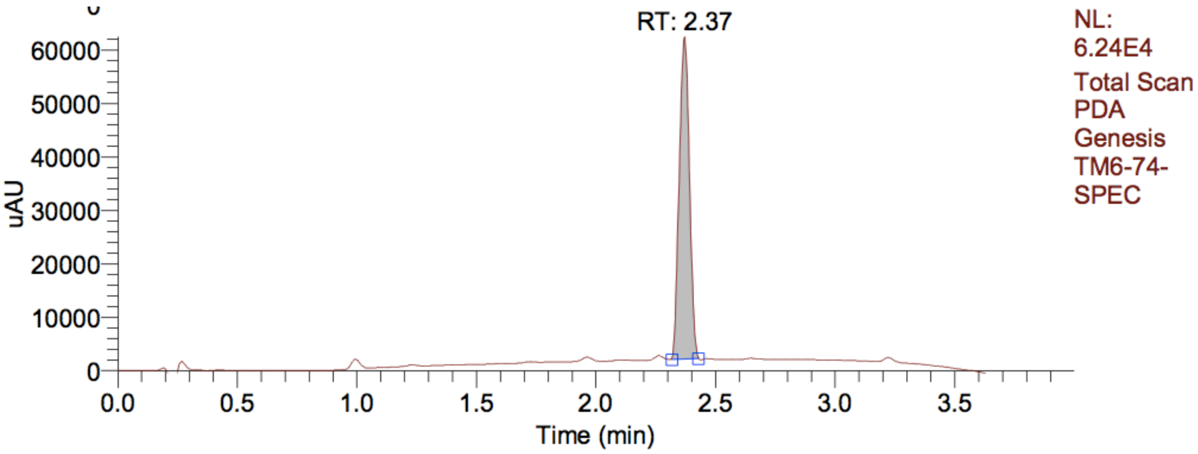


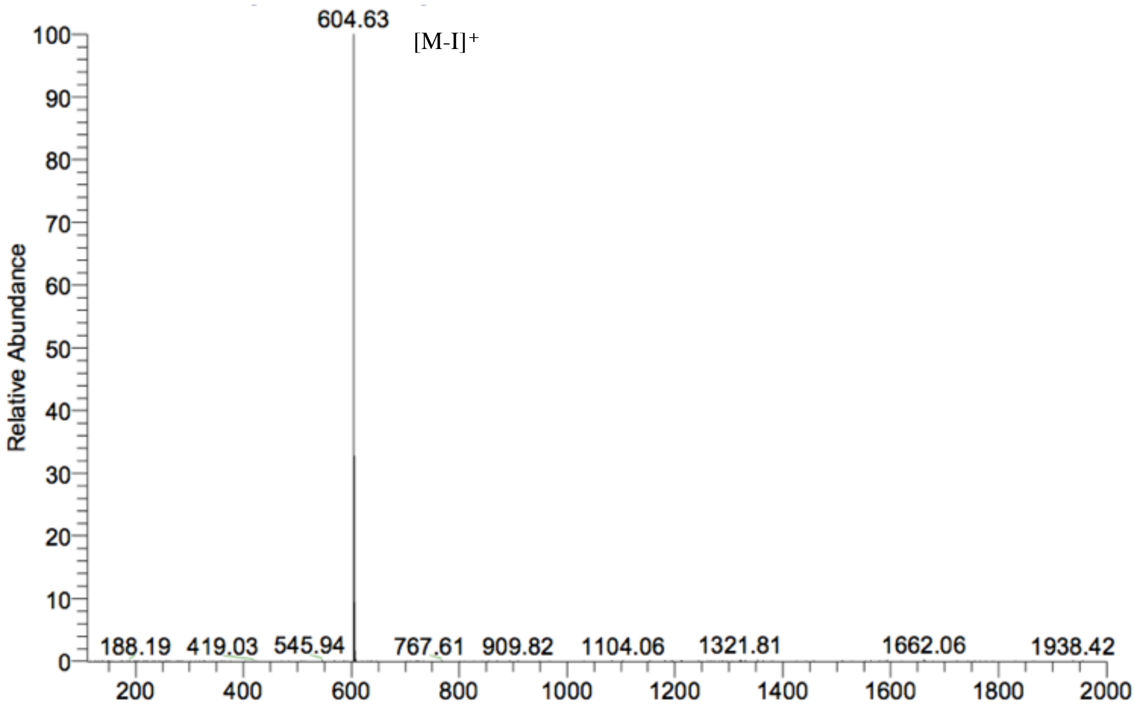

## H-NMR

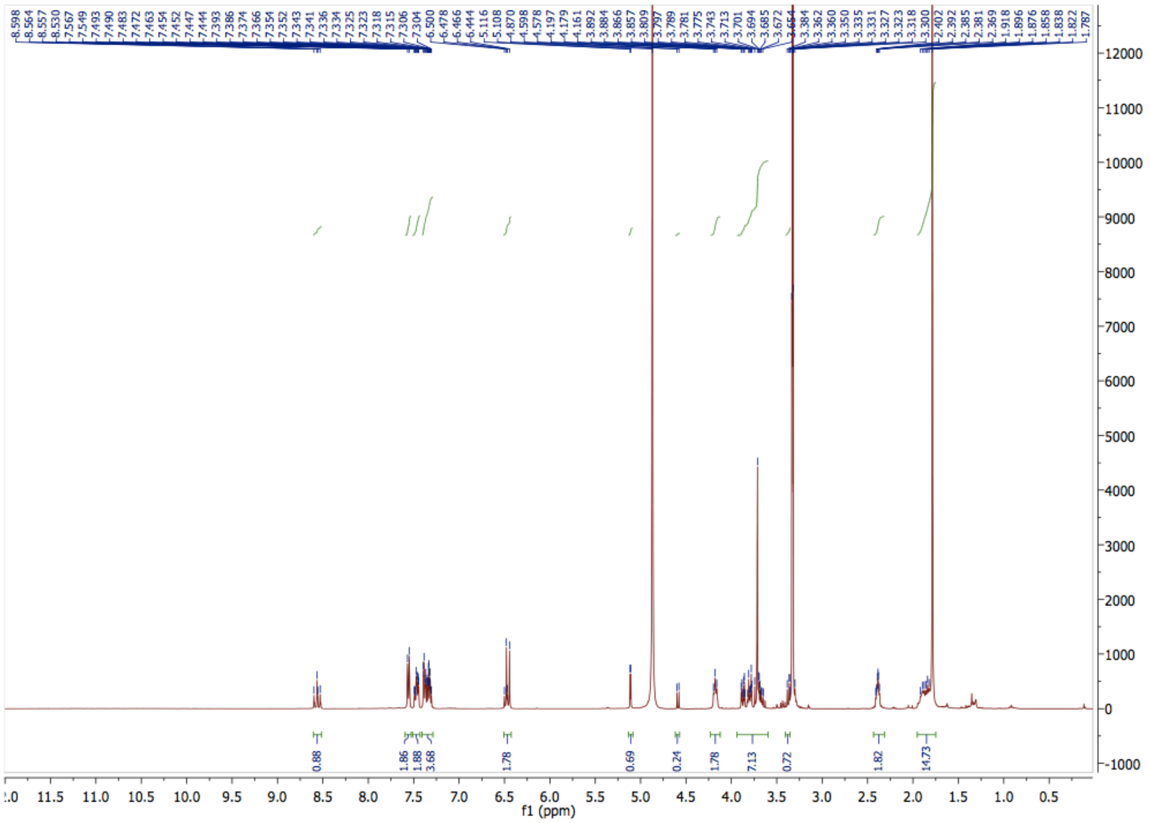

## $C^{13}$-NMR

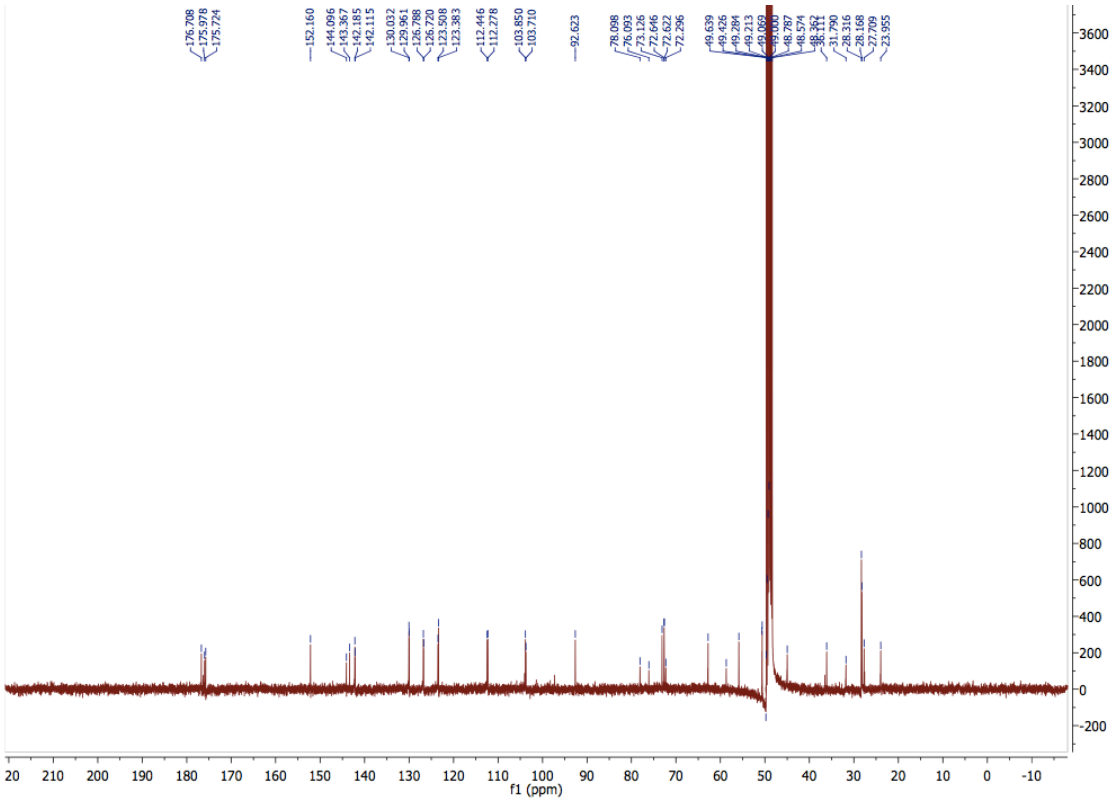

**_N_-CY3-Glucosamine-azide (S5)**

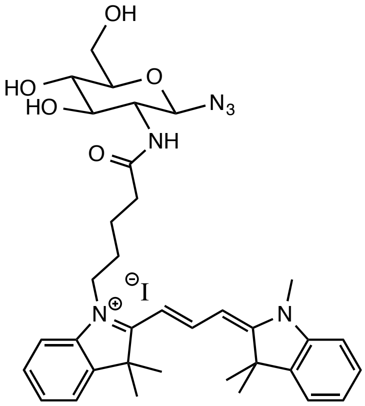


**S5**

## LC-MS

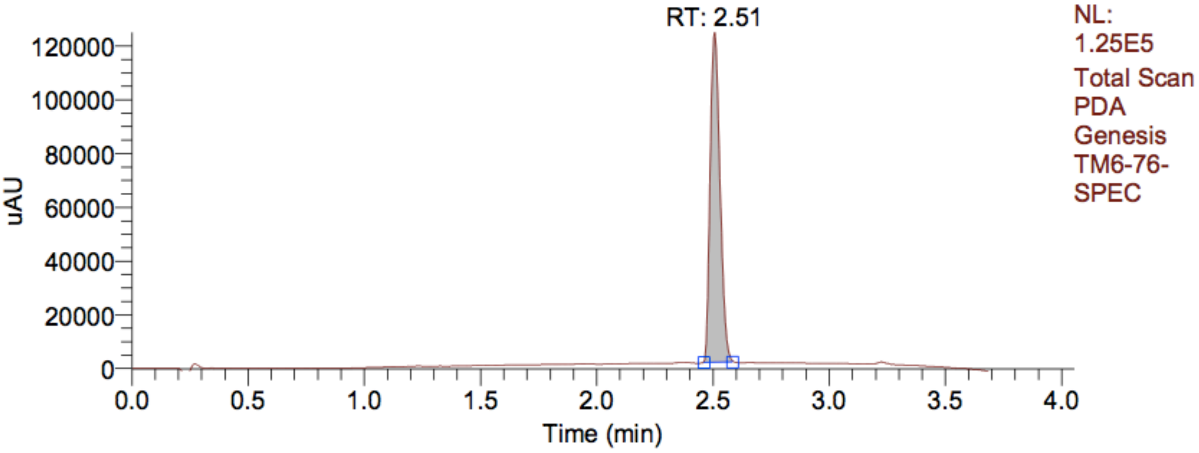


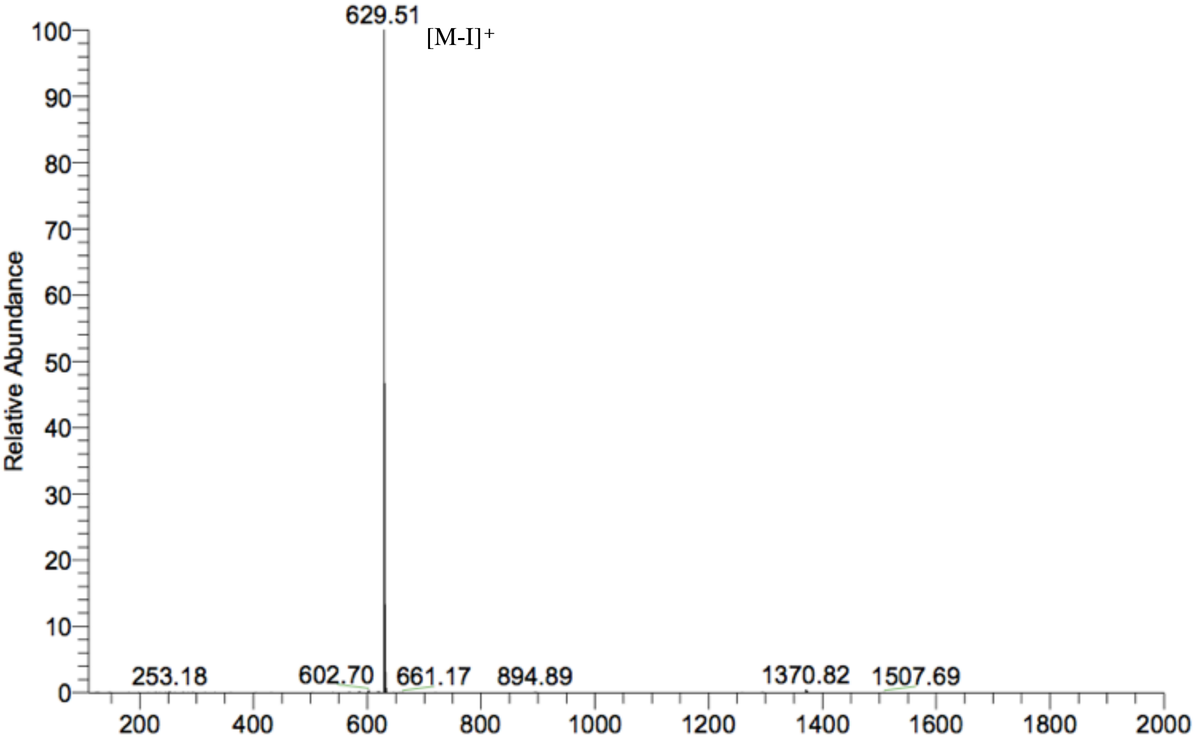

## H-NMR

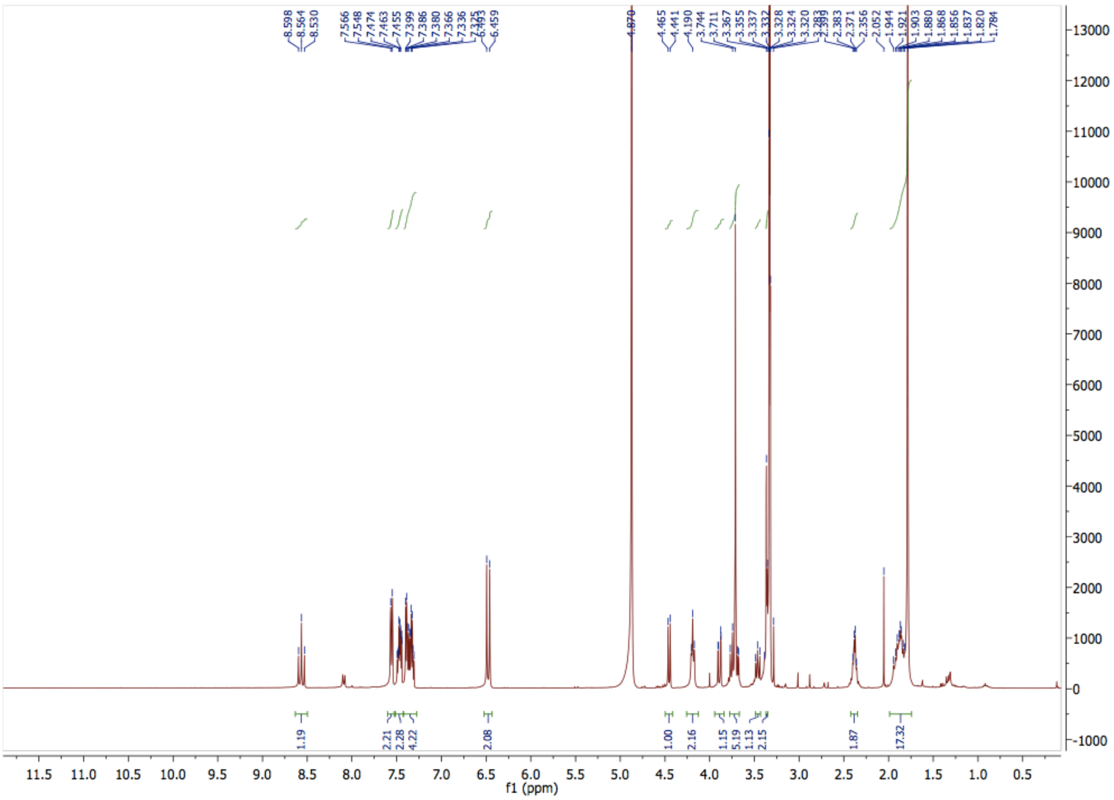

## $C^{13}$-NMR

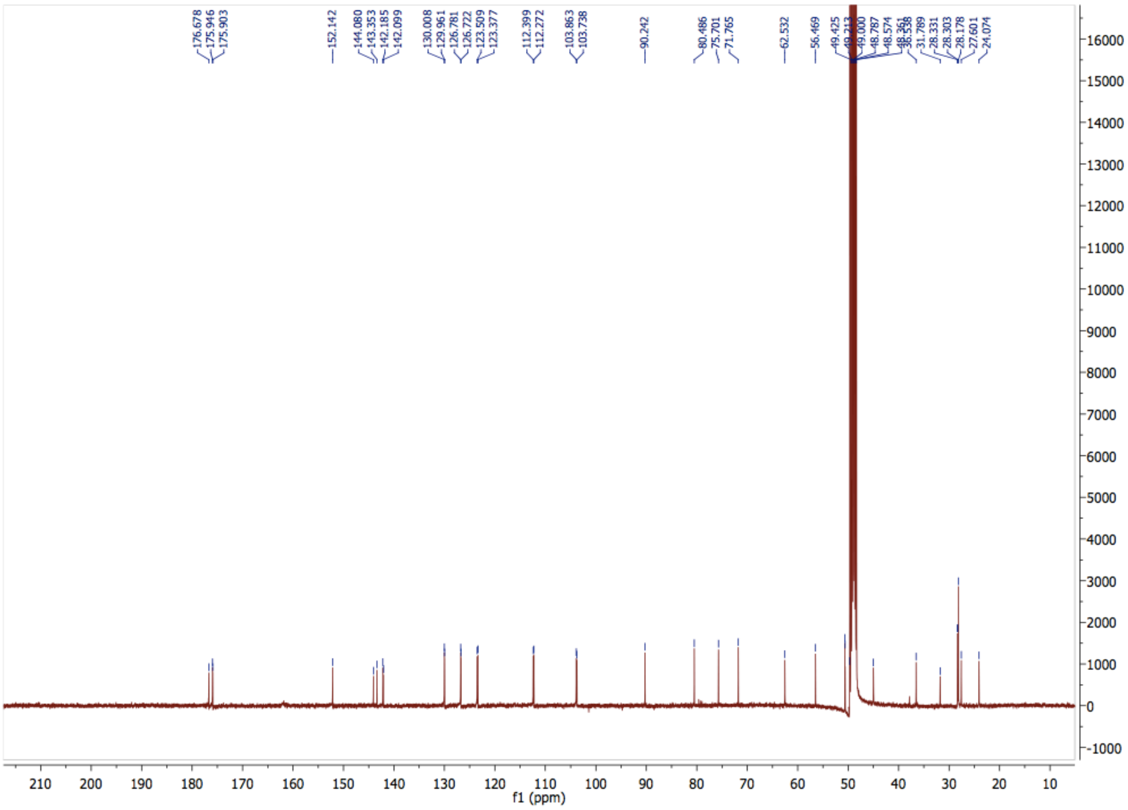

**3-GluNAc-Cy3**

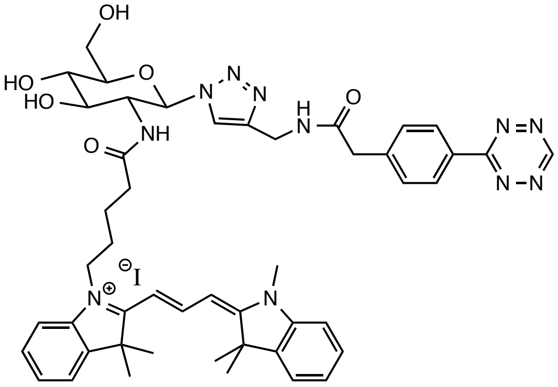


## LC-MS

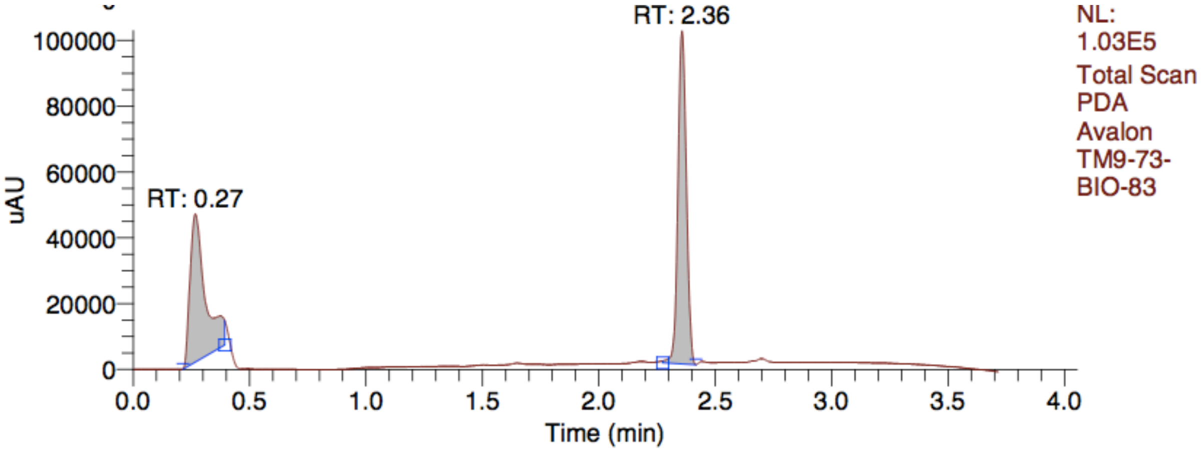


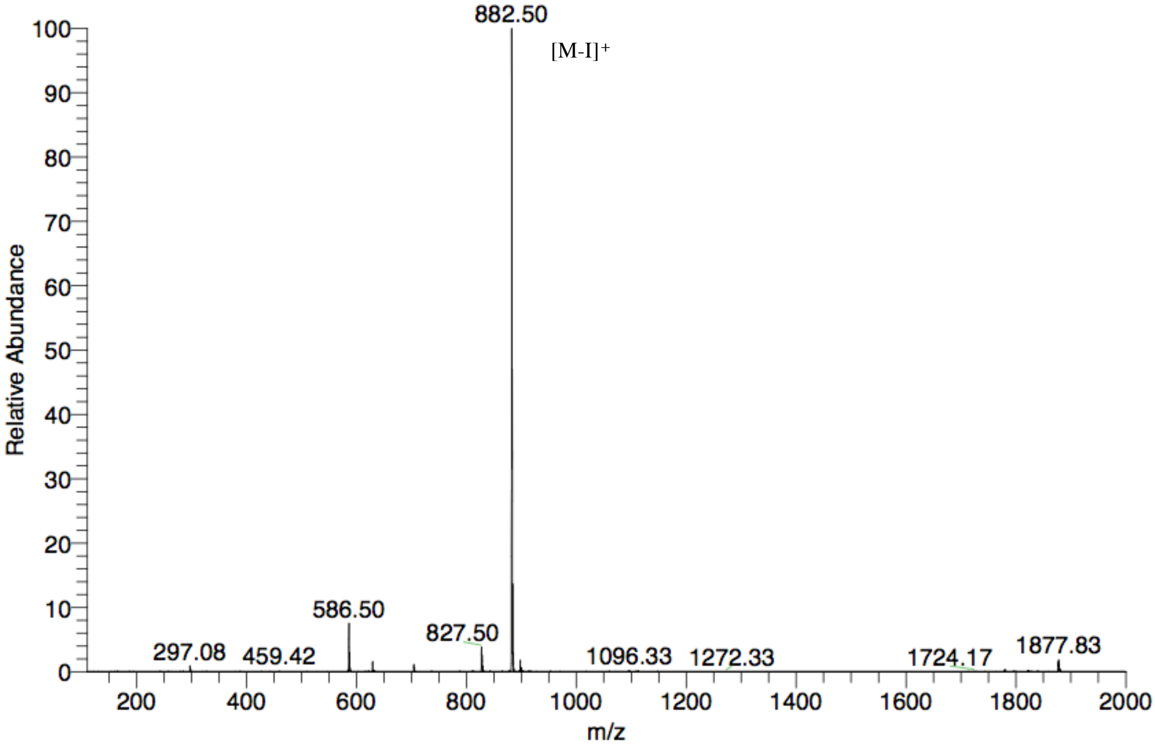

## H-NMR

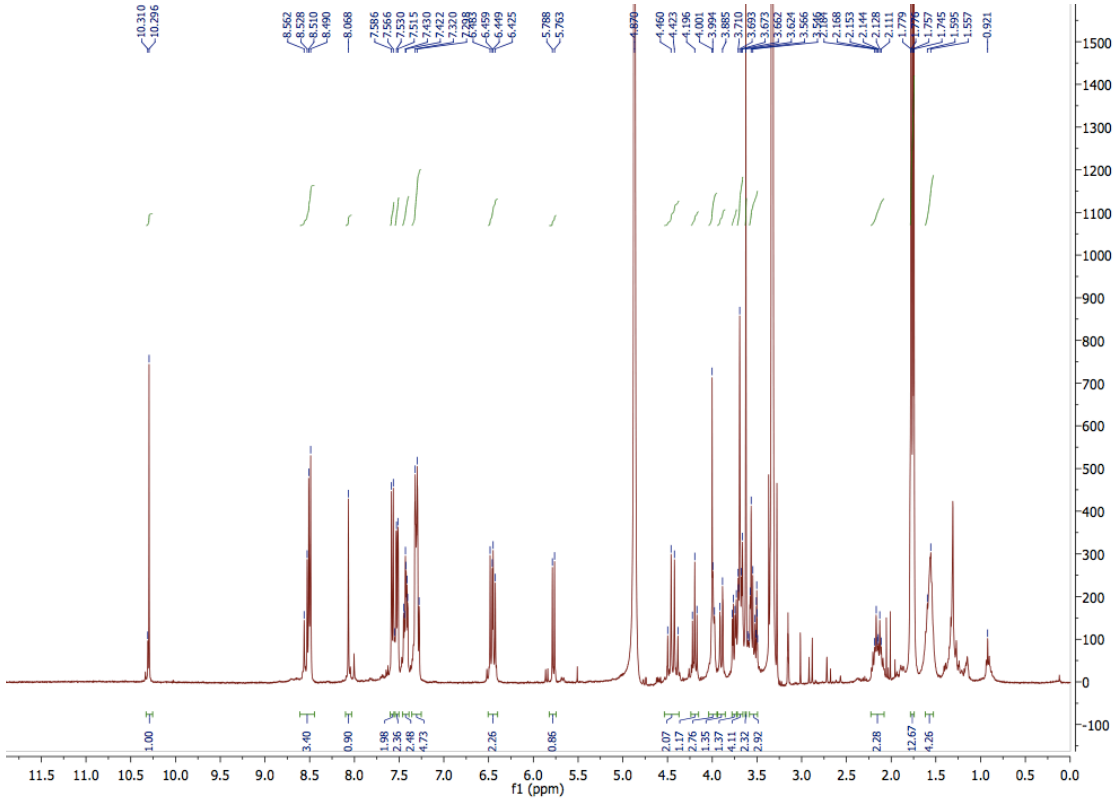

## C13-NMR

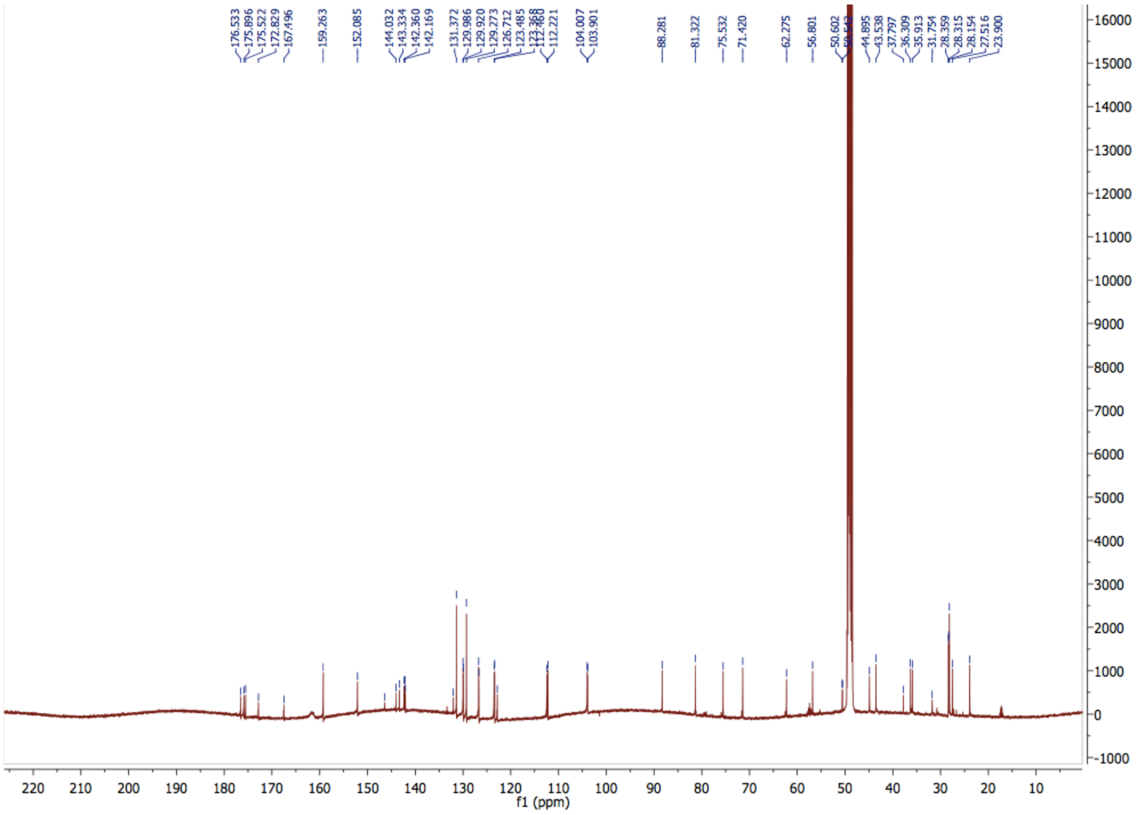

## 13. References

[1] J. Yang, M. R. Karver, W. Li, S. Sahu and N. K. Devaraj, *Angew. Chem. Int. Ed.* **2012**, *51,* 5222-5225.

[2] T. Tanaka, H. Nagai, M. Noguchi, A. Kobayashi and S. Shoda, *Chem. Commun*. **2009,** 3378-3379.

[3] V. Percec, P. Leowanawat, H. J. Sun, O. Kulikov, C. D. Nusbaum, T. M. Tran, A. Bertin, D. A. Wilson, M. Peterca, S. Zhang, N. P. Kamat, K. Vargo, D. Moock, E. D. Johnston, D. A. Hammer, D. J. Pochan, Y. Chen, Y. M. Chabre, T. C. Shiao, M. B. Brlek, S. Andre, R. Roy, H. J. Gabius, P. A. Heiney, *J. Am. Chem. Soc.* **2013**, *135*, 9055-9077.

[4] A. V. Gudmundsdottir, M. Nitz, *Org. Lett*. **2008**, *16*, 3461-3463.

[5] J. A. Villanueva, J. L. Vilas, I, Moreno, J. M. Laza, M. Rodriguez, L. M. Leon, *J. Macromol. Sci.* **2011**, *3*, 211-218.

[6] M. A. Hillmyer, W. R. Laredo, R. H. Grubbs, *Macromolecules* **1995**, *28*, 6311-6316.

[7] K. J. Shea, J. S. Kim, *J. Am. Chem. Soc*. **1992**, *114*, 3044-3051.

[8] K. Lang, L. Davis, S. Wallace, M. Mahesh, D. J. Cox, M. L. Blackman, J. M. Fox, J. W. Chin, *J. Am. Chem. Soc.* **2012**, *134*, 10317-10320; K. Lang, L. Davis, J. T. Kolbus, C. Chou, A. Deiters, J. W. Chin, *Nat. Chem*. **2012**. *4*, 298-304.
